# Absence of conserved immune signalling pathways and increased pathogen susceptibility associated to photosymbiosis in acoels

**DOI:** 10.1186/s12915-026-02506-w

**Published:** 2026-01-23

**Authors:** Francesca Pinton, Nadezhda N. Rimskaya-Korsakova, Katja Felbel, Elisabeth Grimmer, Andreas Hejnol

**Affiliations:** Institute of Zoology and Evolutionary Research, Friedrich Schiller University Jena, Erbertstraße 1, Jena, 07743 Germany

**Keywords:** Immunity, Photosymbiosis, Acoela, Bleaching, *Vibrio*, Pattern recognition receptors, Complement system, Toll pathway, Phagocytosis

## Abstract

**Background:**

Host immunity plays an important role in coral symbiosis with dinoflagellates. Photosymbiosis (the association between hosts and photosynthetic endosymbionts) has evolved multiple times within animals, e.g. within acoels, which are soft-bodied marine invertebrates whose immunity remains so far undescribed.

**Results:**

Our predicted proteome searches show that acoels lack major signal transduction pathways usually involved in animal immunity. Their loss in acoels predates the occurrence of photosymbiosis in this clade. Immune challenges with the coral pathogen and bleaching agent, *Vibrio coralliilyticus*, increase acoel mortality and decrease symbiont abundance in adults of the photosymbiotic acoel *Convolutriloba macropyga*. Mortality in aposymbiotic *C. macropyga* juveniles or aposymbiotic species *Hofstenia miamia* is not affected. Ultrastructural studies of immune-challenged animals by transmission electron microscopy show damages at the cellular and organelle level, as well as a degradation of potential pathogens by the host. In situ hybridisation and differential gene expression analysis point to some areas of interaction between pattern recognition receptors and microbes, as well as to the involvement of acoel-specific or uncharacterised genes.

**Conclusions:**

Based on our findings, photosymbiosis evolution in acoels could have been favoured by the loss of immune signalling pathways. Photosymbiosis in acoels seems to increase susceptibility to pathogen exposure and is disrupted by pathogens. Our data also suggests phagocytosis of pathogens and the possibility of a novel molecular immune response specific to acoels.

**Supplementary Information:**

The online version contains supplementary material available at 10.1186/s12915-026-02506-w.

## Background

Photosymbiosis is the association between a host and endosymbionts capable of photosynthesis [1]. It is usually considered a mutualism, with the host providing protection and inorganic compounds, while receiving photosynthates from the endosymbionts [1–4]. The best studied example of photosymbiosis in animals is the one between cnidarians—corals or sea anemones—and dinoflagellates. This association is crucial for the coral reef ecosystem and increasingly endangered by climate change [5–10]. Increasing water temperatures, ocean acidification, and other stressors bring to a disruption of the coral-dinoflagellate photosymbiosis (i.e., dysbiosis) [11–17], with loss of dinoflagellate pigments or loss of the dinoflagellates themselves. Symbiont loss can happen by degradation, expulsion by the host, detachment, or death of the animal cell hosting them [18–20]. Dysbiosis in corals is associated with the appearance of white spots (bleaching), increased mortality, and susceptibility to diseases [21–23]. Bacterial pathogens can also cause coral bleaching, as well as tissue lysis [21, 24–26]. Research on the immune system of cnidarians has yielded important insights on the establishment, maintenance, and disruption of cnidarian photosymbiosis [1, 19, 27], as well as on the evolution of innate immunity [28–31].

Cnidarians are not the only animals hosting photosynthetic endosymbionts. Photosymbiosis is a widespread phenomenon in animals and has evolved several times, involving a great variety of photosynthetic partners [1, 3, 4]. Addressing photosymbiosis and its link to immunity in an evolutionary context is crucial to understand its underlying mechanisms and to make accurate predictions. To this end, we need to expand the pool of photosymbiotic systems studied [1, 32].

Acoela (Xenacoelomorpha) are flat soft-bodied bilaterians, mostly found in marine habitats [33–35]. Some acoel species rely exclusively on photosymbiotic endosymbionts for nutrition (e.g., *Symsagittifera roscoffensis*), others regularly feed but are still dependent on their symbionts (e.g., *Convolutriloba macropyga*), and some others do not establish photosymbiotic relationships at all (e.g., *Hofstenia miamia*) [36, 37]. Multiple photosymbiotic acoel species inhabit tropical reefs, alongside corals [38]; some are even considered parasitic to corals [39–41]. An increase in temperature causes symbiosis disruption and mortality in *Convolutriloba* species [37, 42]. Water acidification, on the contrary, does not cause mortality in the acoel *S. roscoffensis*, and it only leads to bleaching by symbiont expulsion if extremely high [43]. Acoel responses to pathogens, including bleaching-inducing pathogens, remain uncharacterised. Furthermore, photosymbiosis interaction with the immune system has yet to be understood in this clade.

Here, we investigate the relationship between photosymbiosis and the immune system in acoels. First, our findings on xenacoelomorph immune gene repertoire and photosymbiosis presence are presented in an evolutionary context. Then, we use in vivo immune challenges to characterise acoel responses to the cnidarian pathogen and bleaching agent *Vibrio coralliilyticus*. We find an increased mortality in photosymbiotic acoels, but not in non-photosymbiotic ones. In photosymbiotic acoels, tissue damage can be observed, as well as symbiosis disruption. Degradation of bacteria by phagocytosis in the digestive system can be observed. Our investigation of molecular responses to pathogens suggests the possibility of completely novel immune mechanisms in acoels.

## Results

### Photosymbiosis likely evolved twice within Acoela

**Fig. 1:**
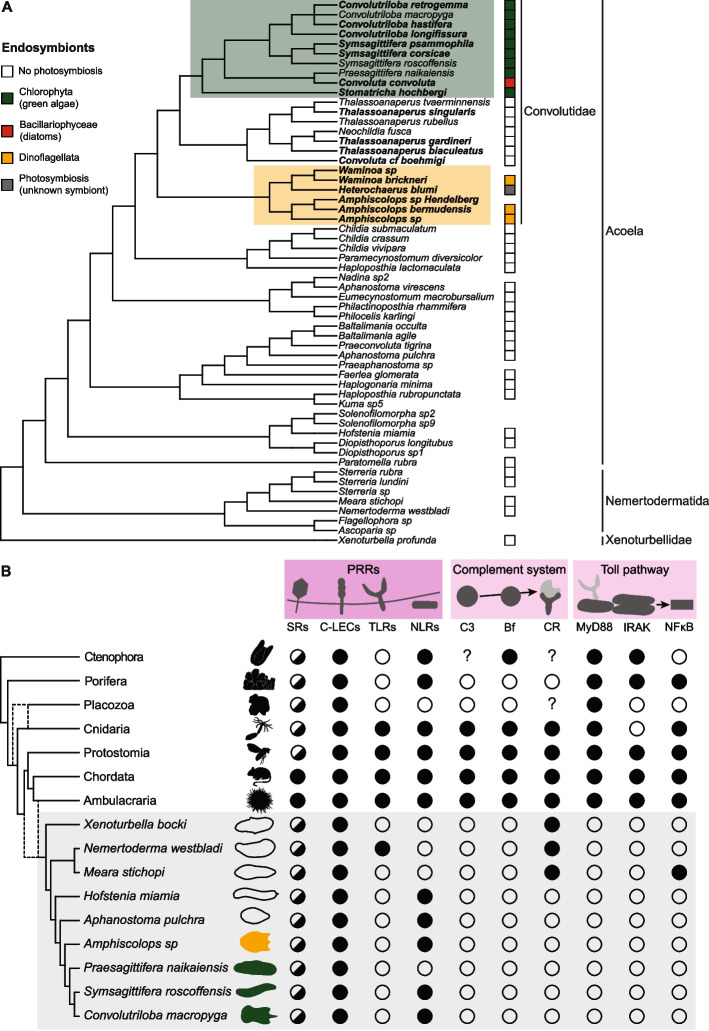
Photosymbiosis and immune genes in Xenacoelomorpha. **A** Photosynthetic endosymbiont presence and type mapped on the phylogenetic tree of Xenacoelomorpha. Tree after Abalde and Jondelius [102], relationships between species in bold after Jondelius et al. [103]. No square means no data available. Presence and type of photosynthetic endosymbionts for each species can be found in Table S1, together with references for each observation. **B** Immune genes in Xenacoelomorpha and other animal groups: black = present; white = absent; half circle = only some types present; question mark = no data. Metazoan phylogeny after Dunn et al., Laumer et al., Schultz et al., Najle et al., Cannon et al., Philippe et al. [104–109]. The dashed lines show the two possible positions of a clade. Xenacoelomorpha highlighted in grey. Some silhouettes are from Phylopic (www.phylopic.org – credits to Andreas Hejnol, Soledad Miranda-Rottermann, Noah Schlottman, Michelle Site, Marina Vingiani, Jake Warner). Sources for gene presence/absence in non-xenacoelomorph metazoans: Kamm et al. [110] for all genes in Placozoa; Orús-Alcalde et al. [111] for TLRs; Song et al. [112] for Toll pathway in Porifera, Cnidaria, Protostomia, Chordata; Orús-Alcalde et al. [29] for Toll pathway and complement system in Porifera, Cnidaria, Protostomia, Chordata, Ambulacraria; Rathinam et al. [31] for NLRs in Porifera, factor B in Ctenophora, SRs, C-lectins, NLR in Ambulacraria; Zelensky and Gready [113] for C-lectins in Porifera, Cnidaria, Protostomia, Chordata; Neubauer et al. [114] for SRs in Cnidaria and Chordata; Pancer et al. [115] for SRs in Porifera; Canton et al., Melo Clavijo et al. [116, 117] for SRs in Protostomia; Zhu et al. [118] for NLRs in Ctenophora, Porifera, Cnidaria, Protstomia, Ambulacraria, Chordata; Koutsouveli et al. [119] for PRRs, MyD88 in Ctenophora; Traylor-Knowles et al. [120] for IRAK and NFkB in Ctenophora. In case of conflicting information for Ctenophora [119, 120], we preferred gene presence/absence inferred from domain searches and genomic data for completeness and comparability

To investigate the coevolution of photosymbiosis and the immune system in acoels, we started by mapping the presence and type of photosynthetic endosymbionts on the phylogenetic tree of acoels and their closest relatives (Fig. 1A, Additional File3: Table S1 [37, 38, 42, 44–101]). We followed the most robust Xenacoelomorpha phylogeny to date [102]. Since it does not feature many species of Convolutidae—the acoel clade containing all photosymbiotic species—, we followed Jondelius et al. [103] for phylogenetic relationships within Convolutidae. Photosynthetic endosymbionts can be green algae (Chlorophyta), dinoflagellates (Dinoflagellata), or diatoms (Bacillariophyceae) [36]. All photosymbiotic acoels belong to Convolutidae and cluster compactly in two groups: (1) species with green algae as endosymbionts and *Convoluta convoluta*—the only species with diatom symbionts; (2) species in symbiosis with dinoflagellates. A third Convolutidae clade, sister to (1), only contains non-photosymbiotic species.

These three clades are well supported in the original studies, with Ultrafast bootstrap/SH-like approximate likelihood ratio test > 95 [102] or posterior probability > 0.90 [103]. This supports the claim that photosymbiosis evolved twice in acoels [38], always within Convolutidae, but once with dinoflagellates and once with green algae or diatoms (clades highlighted in Fig. 1A).

### Pattern recognition receptors are mostly conserved in acoels, while innate immune signalling pathways are absent

To characterise the acoel immune system, we first searched for innate immune genes in available Xenacoelomorpha genomes and transcriptomes (Fig. 1B). We focused on genes that are conserved in metazoans and involved in pattern recognition and signal transduction, especially the ones important for cnidarian-dinoflagellate symbiosis [1, 19, 27, 121]. Since immune genes are often under rapid directional selection [122, 123] and acoels are fast evolving [102], we searched for predicted proteins based on the presence of conserved domains in the expected order (Additional File 1: Fig. S1), and, when relevant, based on their phylogenetic relationships (Additional File 1: Fig. S2–5).

Pattern recognition receptors (PRRs) are responsible for the first interaction with microbes [124]. We searched for domain patterns corresponding to scavenger receptors (SRs), C-type lectins, Toll-like receptors (TLRs), and NOD-like receptors (NLRs) (Additional File 1: Fig. S1A). SRs are characterised by a class-specific domain structure and not by phylogenetic relatedness [114, 116, 117, 125–127]. No predicted protein in the xenacoelomorph species investigated shows domain combinations characteristic of SR class A. SR class B are found in all investigated species, although *Nemertoderma westbladi* sequences have only one transmembrane region instead of 2 (Additional File 1: Fig. S1A). SR class E are found in all investigated species and SR class I only in the xenoturbellid *Xenoturbella bocki* and in the acoels *Hofstenia miamia* and *Aphanostoma pulchra*. C-type lectins are found in all xenacoelomorph species investigated [29, 128]. Toll-like receptors (TLRs) were described as lost in Xenacoelomorpha [111]. However, we find their characteristic domains [111, 129] in the nemertodermatid *N. westbladi*, which was not included in the previous study. NOD-like receptor (NLR) features [118] are present in all acoels investigated apart from *Praesagittifera naikaiensis*, but are absent from non-acoel Xenacoelomorpha. We find sequences from all species, however, containing NACHT domains without Leucin-rich repeats.

As signalling pathways, we examined the Toll pathway and the complement system. Three activation pathways for the complement system are known, all converging to the cleavage, and consequent activation, of C3 [29, 130, 131]. We therefore focused on C3, along with components of the alternative pathway, the most robustly conserved across metazoans [29, 132–134] (Additional File 1: Fig. S1B). We find xenacoelomorph predicted proteins containing α2-macroglobulin domains, but not the other domains of metazoans’ C3 [29, 128, 135–138] (Additional File 1: Fig. S1B); besides, they are more closely related to other genes of the alpha-2-macroglobulin family than to C3 (Additional File 1: Fig. S2) [138]. Factor B domain combination is not present in predicted proteins from the investigated xenacoelomorph species [29, 136, 139–142]. Sequences with the domain pattern of complement receptors 1 and 2 [29, 139, 143] are found only in the non-acoel xenacoelomorphs *X. bocki*, *N. westbladi*, and *Meara stichopi*, and they cluster together with metazoan CR1/2 (Additional File 1: Fig. S1B, S3). The Toll pathway comprises the receptors TLRs, three signalling mediators—MyD88, Tube/IRAK4, Pelle/IRAK1—and the transcription factor NFκB [29, 144–149] (Additional File 1: Fig. S1C). We find predicted proteins with both MyD88 domains—TIR and Death Domain [29, 148, 150]—only in the non-acoel xenacoelomorphs *X. bocki*, *M. stichopi*, and *N. westbladi*. However, when running a phylogenetic analysis, these sequences do not cluster with other metazoans’ MyD88, but with human TIRAP, though with low support values (Additional File 1: Fig. S4). The domains characterising IRAK4/Tube and IRAK1/Pelle—Interleukin-1 receptor-associated kinases (IRAKs)—are not featured together in any predicted proteins from the investigated xenacoelomorphs [29, 146]. While we find sequences from all species containing Rel/nuclear factor-κB (NFκB) domains [29, 151, 152], according to the phylogenetic analysis only *M. stichopi* sequences can be considered NFκB (Additional File 1: Fig. S5). *Xenoturbella* and nemertodermatids also contain sequences more closely related to Rel proteins, and all the investigated species possess sequences clustering within the NFAT (nuclear factor of activated T-cells) family (Additional File 1: Fig. S5) [152].

Summarising, PRRs are mostly conserved in acoels, with the exception of TLRs. Signalling pathway components of the innate immune system (toll pathway and complement system) are partially absent in non-acoel xenacoelomorphs and completely absent in acoels.

### Mortality upon immune challenge increases in photosymbiotic acoels, not in aposymbiotic ones

We then investigated the link between photosymbiosis and immunity with in vivo immune challenges of photosymbiotic and non-photosymbiotic (i.e. aposymbiotic) acoels. Our hosts of choice are *Convolutriloba macropyga* photosymbiotic adults and aposymbiotic juveniles [37], as well as the aposymbiotic species *Hofstenia miamia* [153] (distance to *C. macropyga*: 1.2 substitution per site [102])*.* No pathogens of acoels are yet known, so we selected the cosmopolite and generalist Gram-negative bacterium *Vibrio coralliilyticus* as immune agent [154]. *V. coralliilyticus* is distributed worldwide [155] and it is pathogenic to a variety of marine animals, such as corals, sea anemones, fish, bivalves, crustaceans, and sea urchins [154, 156–163], as well as to fruit flies in laboratory conditions [164, 165]. Moreover, exposure to *V. coralliilyticus* increases mortality in the dinoflagellate *Symbiodinium* [165] and disruption of its symbiosis with corals [161, 166–170]—although not with sea anemones [163].

**Fig. 2:**
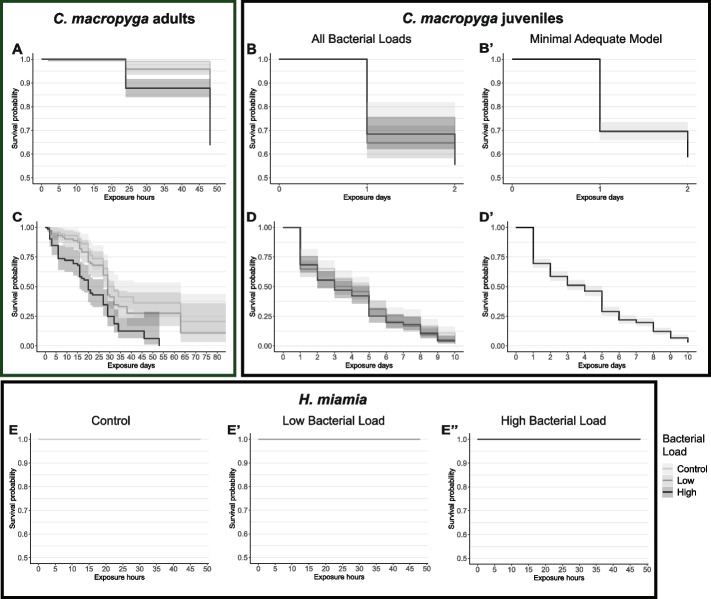
Survival curves of acoels upon *Vibrio coralliilyticus* exposure with 95% confidence intervals. Data were fitted to mixed effects Cox proportional hazard models, full statistics in Additional File 4: Table S2. Two days of bacterial exposures: **A** *C. macropyga* adults (minimal adequate model Surv(last.obs, censored) ~ Bacterial.Load + (1 | batch), *χ*^2^ = 59.742, *p* = 1.065e-13, number of replicates = 4, *n* = 863); **B**–**B’** *C. macropyga* juveniles (minimal adequate model Surv(last.obs,censored) ~ 1, number of replicates = 4, *n* = 569); **E**–**E’** *H. miamia* adults (number of replicates = 5, *n* = 154). Long-term exposures: **C** *C. macropyga* adults (minimal adequate model Surv(last.obs, censored) Bacterial.Load + (1 | batch), *χ*^2^ = 8.646, *p* = 6.02e-07, number of replicates = 3, *n* = 216); **D**, **D’** *C. macropyga* juveniles (minimal adequate model Surv(last.obs,censored) ~ 1, number of replicates = 4, *n* = 569). For *C. macropyga* juveniles, graphs representing the survival for all bacterial loads (**B**, **D**) are added alongside the ones showing the minimal adequate model (**B’**, **D’**). In **B’** and **D’**, the colour of the curve is not meaningful, since survival is independent of bacterial load. Note that the *y* axis lower limit is 0.5 for the 2-day graphs

The animals were exposed for 2 days to a low or high bacterial load—10^5^ and 10^6^ CFUs (colony forming units), respectively. *C. macropyga* adult mortality is affected by bacterial dose and by batch (Fig. 2A, Additional File 4: Table S2). Mortality is significantly higher for high-bacterial-load samples than for controls or low-bacterial-load samples (HR = 4.65 ± 0.21, Bonferroni-adjusted *p* < 0.0001 for both post hoc contrasts). Mortality is also higher for low-bacterial-load samples than for controls, although with lower hazard and lower significance (HR = 2.23 ± 0.23, Bonferroni-adjusted *p* = 0.0012). To confirm the active role of *V. coralliilyticus* in causing *C. macropyga* mortality, we also performed immune challenges with heat-inactivated bacteria. A high dose of heat-inactivated bacteria does not increase mortality compared to controls (Additional File 1: Fig. S6A). We also tested susceptibility upon exposure to *Priestia megaterium*, a Gram-positive bacterium also found in diseased corals [171, 172]: mortality increase is even starker at a high bacterial load, yet none at a low bacterial load (Additional File 1: Fig S6B, Additional File 4: Table S2).

The mortality of *C. macropyga* juveniles is not affected by bacterial load nor by batch (Fig. 2B–B’). Immune-challenged *H. miamia* survived regardless of bacterial load, except one animal in the high dose sample (Fig. 2E–E’’). Besides, all individuals (*n* = 25) exposed to an even higher dose of 2.5∙10^7^ CFUs also survived. In some wells, despite unaffected animal morphology, spheres of tissue could be observed in solution, and in one individual, a sphere appeared from the posterior end of the body and another one was expelled through the mouth (Additional File 2: Video S1).

We also carried out longer immune challenge assays in *C. macropyga* adults and juveniles, to check for a delayed effect on survival. They were monitored for 10 days (juveniles), 1 month (2 batches of adults), or 3 months (1 batch of adults). In a similar way to the 2-day assays, the bacterial load affected survival for adults and not for juveniles (Fig. 2C, D’).

To summarise, survival in aposymbiotic *H. miamia* and *C. macropyga* juveniles is seemingly not impacted by exposure to *V. coralliilyticus*, while mortality increases in symbiotic *C. macropyga* adults.

### Bacterial distribution within immune-challenged *Convolutriloba macropyga*

**Fig. 3:**
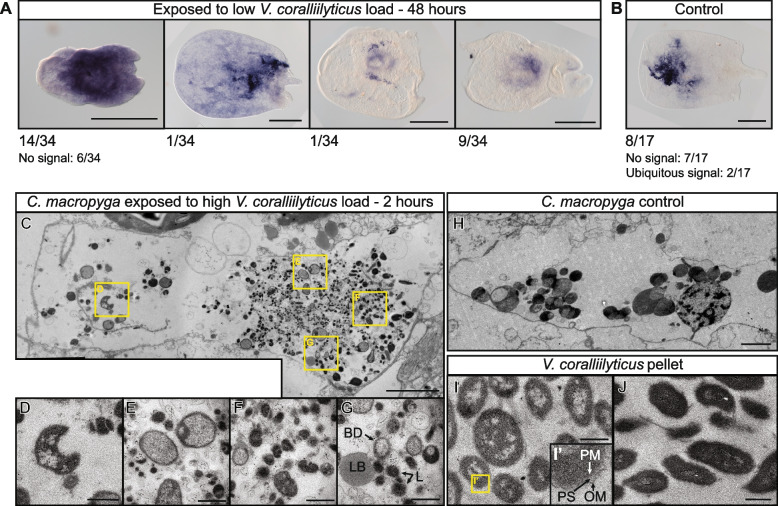
Presence of *Vibrio* in immune challenged *Convolutriloba macropyga*. RNA in situ hybridisation against *V. coralliilyticus* 16S in *C. macropyga* exposed for 48 h to (**A**) a low load of *V. coralliilyticus* and (**B**) resuspended marine broth (control). Numbers indicate the ratio of individuals with the pattern shown above; dorsal view, anterior is facing left. Transmission electron microscopy images of phagocytosis in the digestive parenchyma in *C. macropyga*, cross-section at the level of the mouth: **C**–**G** exposed to a high load of *V. coralliilyticus* for 2 h, containing degraded bacteria; **H** 2-h exposure control, showing physiological phagocytosis; **I**, **J** cultured and pelleted *V. coralliilyticus*. Yellow square magnifications: **D** abnormal bacterial shape and absence of periplasmic space and outer membrane typical of gram-negative bacteria; **E** abnormal pale granulated cytoplasm of the pathogens; **F** numerous osmiophilic lysosomes; **G** bacterial debris (empty cell walls) (BD), lipid or lipofuscin body (LB), lysosomes (L) [174, 175]; **I’** hallmarks of gram-negative bacteria: outer membrane (OM), a well-defined periplasmic space (PS), plasma membrane (PM) [176]. Scale bars are 0.5 mm in (A,B), 3 µm in (C,H), and 0.5 µm in (D-G,I,J)

To confirm infection of *C. macropyga* upon exposure, we performed in situ hybridisation against *Vibrio coralliilyticus* 16S rRNA on individuals exposed 2 days to a low bacterial dose (Fig. 3A). A variety of patterns can be observed, from individuals showing no signal at all (6 out of 34) to almost ubiquitous signal (14 out of 34), with multiple instances of signal localised in the digestive system area. In half of the control samples (8 out of 17), bacterial 16S rRNA is also observed around the anterior region of the digestive system (Fig. 3B). While in most of the other samples no signal can be found, in two of them bacterial RNA is present in the whole sample. In individuals exposed to *V. coralliilyticus* for shorter times, we see similar patterns, with signal for *V. coralliilyticus* 16S both in exposed individuals and in controls (Additional File 1: Fig. S7). It can also be found in correspondence to the anterior nerve cords in some samples, and in asexual reproductive buds. To confirm the presence of 16S rRNA from *V. coralliilyticus* in *C. macropyga*, we checked for its amplification by PCR from *C. macropyga* cDNA (Additional File 1: Fig. S8). The probe itself does not yield any hits when BLASTed against *C. macropyga* transcriptome, and the primers do not amplify anything in silico from *C. macropyga* transcriptome (see the “Methods” section). Therefore, it is reasonable to consider the bands as from two diverging sequences of 16S rRNA of *V. coralliilyticus* or a related bacterial species. We also checked for PCR amplification of *V. coralliilyticus* virulence factors [173]: 7 genes could not be amplified, but for one gene (WP_006961766.1), a single band is obtained from *V. coralliilyticus*, two from *C. macropyga* (Additional File 1: Fig. S8).

To detect *V. coralliilyticus* in *C. macropyga-*infected tissues and the host response, we performed transmission electron microscopy (TEM) on the brain, the body wall, and the internal parenchyma in the digestive system area (i.e., the areas showing signal for *V. coralliilyticus* 16S rRNA, Fig. 3A, B). We could not find any bacteria in individuals exposed for 2 days to a low (*n* = 2) or a high load (*n* = 2) of *V. coralliilyticus* or to control medium (*n* = 2). We therefore imaged individuals exposed for a shorter period: 1 h of exposure (*n* = 2) and 2 h of exposure (*n* = 2). Potential pathogenic bacteria at various levels of degradation were found only in the digestive system of a 2-h exposure individual (Fig. 3C–G). Signs of phagocytosis—such as lysosomes, phagolysosomes, lipid, or lipofuscin bodies (Fig. 3F,G)—are found also in the digestive parenchyma of control samples (Fig. 3H). Some structures, however, resemble free-living *V. coralliilyticus*, but shrunk and with abnormal features (compare Fig. 3D, E to I, J). Similar characteristics are observed in *V. coralliilyticus* within infected corals [166, 168], although in our samples a wider degree of variation can be seen.

### Symbiotic algae number decreases upon immune challenge in *Convolutriloba macropyga*

**Fig. 4:**
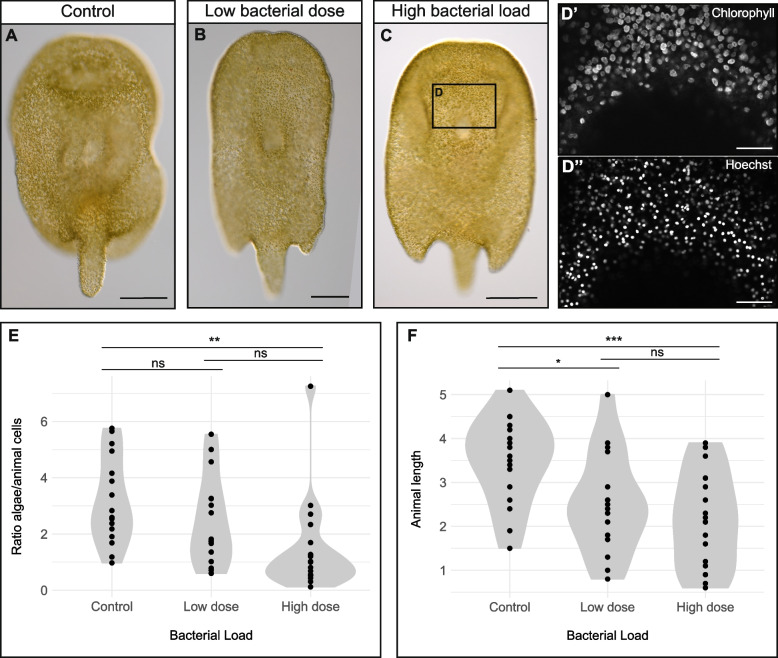
Dysbiosis in *C. macropyga* upon 2-day immune challenge with *V. coralliilyticus*. **A**–**C** DIC-images of *C. macropyga* after 2-day immune challenges with *V. coralliilyticus*; ventral view, anterior facing up. **D’**–**D’’** Chlorophyll autofluorescence and Hoechst 33342 staining in the area corresponding to **D**, as an example of the data used to produce the plots in **E**. **E** Violin plots of the ratio of algal cells to animal cells in immune-challenged animals. Minimal adequate model: Ratio.algae.hoechst ~ Bacterial.Load, number of replicates = 2, sample size = 52, *F* = 5.5069, *p* = 0.00696). Post hoc Tukey HSD tests: high-dose – control *t* = − 3.298, *p* = 0.00514; low-dose – control *t* = − 1.348, *p* = 0.37598; low-dose – high-dose *t* = − 1.861, *p* = 0.16080. Estimated difference between high-dose and control: − 1.7 ± 0.5. Detailed statistics in Additional File 4: Table S3. **F** Violin plots of animal length in immune-challenged animals. Length ~ Bacterial.Load, Anova, *F* = 7.7059, *p* = 0.001231; post hoc Tukey HSD tests: high-dose – control *t* = − 3.847, *p* < 0.001; low-dose – control *t* = − 2.622, *p* = 0.0306; high-dose – low-dose *t* = − 1.093, *p* = 0.5229. For both graphs, *p* values for Tukey HSD tests: ns, *p* > 0.05; *, *p* < 0.05; **, *p* < 0.01; ***, *p* < 0.001. Scale bars are 250 µm in **A** and **B**, 50 µm in **D’**–**D’’**

Disruption of the symbiosis between corals and algae is a known response to environmental stresses and infection by some bacteria, including *Vibrio coralliilyticus* [177]*.* To investigate this in *C. macropyga* immune-challenged with *V. coralliilyticus*, we first looked for whitening of the whole animal or parts of it, as observed in corals [166, 168, 178]. No white areas can be distinguished at 2 days of exposure in fixed and mounted individuals (Fig. 4A–C). At 14 days of exposure, when bleaching is visible in *V. coralliilyticus*-infected corals [166, 168], still no pigmentation changes can be detected in *C. macropyga* (Additional File 1: Fig. S9). We then quantified potential symbiont loss at the tissue level: algal chlorophyll autofluorescence and stained animal cell nuclei were imaged by confocal microscopy in a relatively flat area between the eyespots and the mouth (Fig. 4D–D’’), then automatically detected and counted. At 2 days of exposure, the ratio between algal cells and animal cells is affected by bacterial load (Fig. 4E, Additional File 4: Table S3). Post hoc Tukey HSD tests (Fig. 4E) show that individuals exposed to a high dose of *V. coralliilyticus* have less algae per animal cell than controls, while comparisons of low-dose samples to the other two conditions are not significant. While the algae per animal cell ratio is not affected by animal size (Additional File 4: Table S3), exposure to *V. coralliilyticus* has an effect on animal size itself (Fig. 4F). Both a high dose and a low dose of *V. coralliilyticus* decrease animal size, measured as length, though the effect is starker and more robustly significant for the high dose (Fig. 4F).

### Damaged tissue in immune-challenged *Convolutriloba macropyga*

**Fig. 5:**
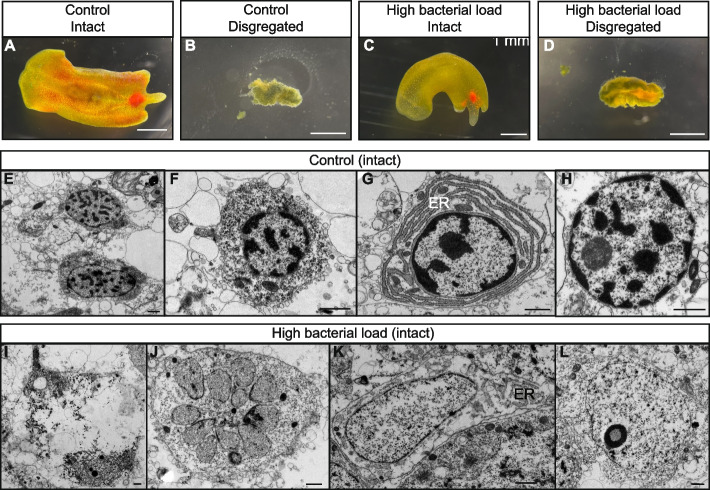
Damage in *C. macropyga* at 2 days of exposure to *V. coralliilyticus*, imaged through a stereo microscope (**A**–**D**), or at ultrastructural level with TEM (**E**–**L**). Features of parenchymal cells in control samples: **E**, **F** granular cytoplasm and nuclei with heterochromatin, **G** endoplasmic reticulum (ER), **H** nucleus with visible nucleolus. Samples exposed for 2 days to a high load of *V. coralliilyticus*: **I** tissue degradation and degenerate organelles; **J** fragmented nucleus; **K** lysis of chromatin and swollen ER; **L** damaged nucleolus. Scale bars are 1 mm in **A**–**D**, 1 µm in **E**–**L**

Immune-challenged *Convolutriloba macropyga* were considered dead when not moving and showing loss of anatomical integrity. They shrink and present structural deterioration, with pieces coming apart (Fig. 5D). The same, however, can be observed for the death of control individuals (Fig. 5B) and is therefore not specific to *Vibrio* pathogenesis.

We searched for changes at the ultrastructural level with transmission electron microscopy in immune-challenged individuals that looked intact and motile. Tissue and cell damage is observed in *C. macropyga* exposed for 2 days to a high bacterial dose (Fig. 5I–L,* n* = 2), when compared to controls (Fig. 5E–H, *n* = 2). Tissue degradation and cell debris are present in the parenchyma (Fig. 5I) [cf. 168]. At a subcellular level, one can see swelling of the endoplasmic reticulum (Fig. 5K) [cf. 179], and segregation of nucleolus components (Fig. 5L) [cf. Fig. 3.22 in 180]. Fragmented nuclei are also present (Fig. 5J), although the lack of chromatin condensation and membrane blebbing rules out apoptosis [181, 182].

### Asexual reproduction is not affected by *Vibrio* exposure

Reproduction contributes to individual fitness alongside survival, and the impact of infections on it can be antipodal [183]. In fact, on one hand, pathogens have been shown to decrease fecundity, likely due to energy constraints [184–186]. On the other hand, organisms sometimes allocate more energy to reproduction when their survival is threatened, a strategy known as terminal investment in reproduction or fecundity compensation [183, 187–191]. We therefore measured fecundity by monitoring the number of asexual progeny released per day upon 2-day immune challenges. Asexual progeny numbers are not affected by bacterial load. They are however affected by presence of an asexual bud prior to bacterial exposure and, in the first day of exposure, by batch (generalised linear mixed model, minimal adequate model for day 1: progeny.released ~ bud + (1 | batch), *χ*^2^ = 24.684, *p* = 6.752e-07, number of replicates = 4, sample size = 813; day 2: progeny.released ~ bud, *χ*^2^ = 18.512, *p* = 1.688e-05, number of replicates = 4, sample size = 669; detailed statistics in Additional File 4: Table S4). Therefore, no changes in reproduction occur upon *V. coralliilyticus* exposure, neither an increase nor a decrease.

### PRRs are expressed around the digestive system, nervous system, and in reproductive structures

**Fig. 6:**
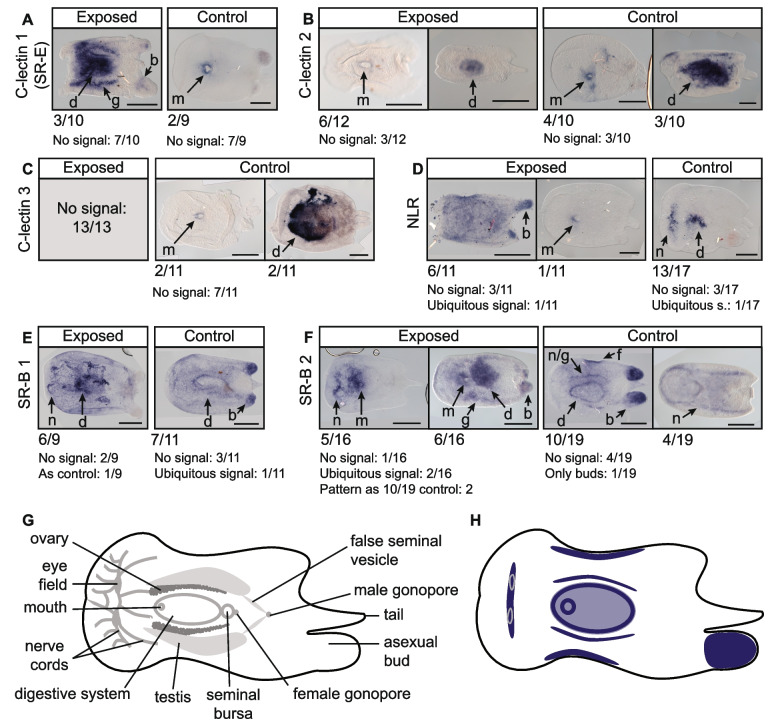
Expression patterns of *Convolutriloba macropyga* PRRs upon immune challenge. **A**–**F** RNA in situ hybridisation against *C. macropyga* PRRs in immune-challenged *C. macropyga* adults (exposed for 48 h to low *V. coralliilyticus* load or control medium). Numbers indicate the ratio of individuals with the pattern above; dorsal view, anterior facing left; scale bars are 0.5 mm. b: bud, d: digestive system, f: flank, g: gonads, m: mouth, n: nervous system, n/g: nervous system or gonads. **G** Morphology of *C. macropyga* after Shannon and Achatz [37]. **H** Schematic of PRR expression domains

To characterise acoel molecular response to infection, we first focused on *C. macropyga* pattern recognition receptors (PRRs) identified as detailed above. Expression patterns for each PRR gene vary widely between individuals, both after exposure to a low dose of *V. coralliilyticus* and in controls (Fig. 6). They are not expressed in some samples at all, and they are expressed in the whole animal in some other samples. The remaining cases can be described as follows. C-lectin gene expression localises around the mouth opening or more broadly in the digestive system for control samples (Fig. 6A–C). Upon bacterial exposure, the changes in expression vary by gene, with one C-lectin widening its expression domain (Fig. 6A, also corresponding to scavenger receptor E domain structure, Additional File 1: Fig. S1), another showing no change (Fig. 6B), and a third not being expressed at all in the exposed samples (Fig. 6C). The NOD-like receptor gene (NLR, Fig. 6D) is expressed in the anterior part of the digestive system and in the nervous system in controls. It either widens or narrows its expression pattern in immune-challenged animals. Scavenger receptors type B (SR-B, Fig. 6E–F) are expressed throughout the body, with increased intensity around the digestive system in controls. Other two domains of expression on the sides of the digestive system could correspond to two nerve cords or to the female gonads. The pattern is less clear-cut for exposed individuals and there seems to be stronger expression in the anterior region of the digestive system, as well as in the nervous system. Even if a clear pattern cannot be derived for all PRRs, we can identify some areas of increased immune receptor presence in acoels (Fig. 6H). Asexual reproduction buds at the posterior end of the animal body, when present, express the immune receptors. Other putative hot spots of immune recognition are the areas around the mouth and around the digestive system, the anterior nervous system, the female gonads—when present—and a faint domain on the flanks of the animals, which, to our knowledge, does not correspond to any described morphological structure in acoels.

### Many genes differentially expressed upon immune challenge are acoel-specific or not characterised

**Fig. 7:**
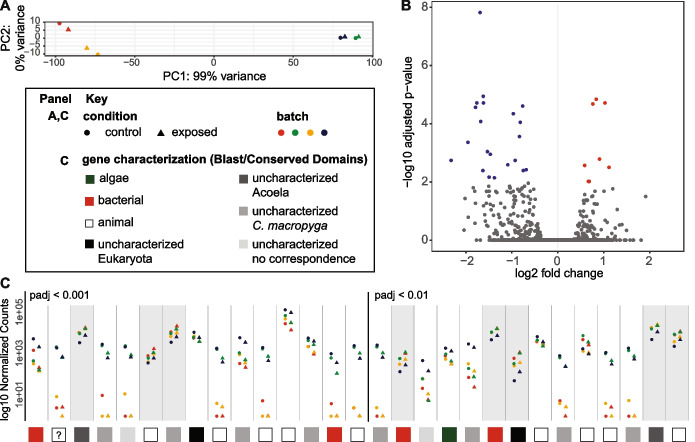
Differentially expressed genes upon immune challenge. **A** Principal component analysis of transformed count data. **B** Volcano plot of differentially expressed genes (DEGs) between control and exposed samples. Downregulated genes in exposed samples are represented by blue dots, upregulated by red dots, and genes below significance threshold (padj < 0.01) by grey dots. **C** Normalised counts for significantly DEGs by batch, ordered by increasing adjusted *p*-value. Genes with grey background are upregulated, with white background downregulated. As detailed in the key, data point shapes and colours are common to **A** and **C** and squares at the bottom of **C** show the results of gene characterisation by BLAST and Conserved Domain Search, detailed in Additional File 5: Table S5. The question mark identifies the sequence corresponding to *Xenia* sp. RxLR effector protein

Given the lack of canonical immune signalling pathways, we looked for genes involved in the response to *Vibrio* by comparing gene expression levels in whole *C. macropyga* adults challenged with a low *V. coralliilyticus* load to controls at 2 days of exposure. In principal component (PC) analysis of count data after variance stabilising transformation, only one PC explains 99% of the variance and the samples cluster by date of infection (batch) and not by condition, i.e. control or exposed (Fig. 7A). Thus, most of the differences between samples are due to batch, not to bacterial exposure status. Twenty-nine genes are differentially expressed upon exposure to *V. coralliilyticus* (8 upregulated, 21 downregulated; FDR-adjusted *p* < 0.01; Fig. 7B,C, Additional File 5: Table S5). When searched against the NCBI non-redundant protein sequence database, half of them (14/29) retrieve uncharacterised proteins or no hits at all. Of those, two match uncharacterised proteins in multiple eukaryotic species, two have no correspondence in xenacoelomorph predicted proteomes, 8 only in *C. macropyga*, and two in other acoels too—but not in non-acoel xenacoelomorphs (Fig. 7C, Additional File 5: Table S5). Four differentially expressed genes match bacterial sequences or conserved domains, though none of them corresponds to *Vibrio* species: hits for the two downregulated sequences are mostly uncharacterised bacterial sequences, while the two upregulated sequences seem related to virulence factors (one as WXG100 family [192]; one with domains corresponding to MAC/Perforin, Hint, and YeeP GTPase [193]). Hits for one downregulated sequence are secreted RxLR effector protein 161-like for the soft coral Xenia sp. Carnegie-2017 (automatically predicted); though these are animal sequences, RxLR effector proteins are usually avirulence factors from pathogenic oomycetes [194]. One sequence matches only an unnamed protein from the green alga *Closterium* sp. and could therefore correspond to an algal sequence. Hits for all other sequences are metazoan genes: one upregulated serine dehydratase, one upregulated transient potential cation channel, 3 downregulated serine proteases, one downregulated dynein regulatory complex subunit, one downregulated adhesion protein, one downregulated carbonic anhydrase, and one downregulated C-lectin domain-containing protein—which does not align to the ones retrieved in our initial gene search. While the latter is the only canonical immune-related gene, serine proteases can also be involved in immunity [195].

## Discussion

### Photosymbiosis in Xenacoelomorpha evolved after the loss of metazoan-conserved immune signalling pathways

We analysed the literature on the presence and type of photosymbionts in xenacoelomorphs (Additional File 3: Table S1), mapped them on the phylogeny (Fig. 1A), and consequently confirmed that photosymbiosis has likely evolved at least twice in acoels, always within Convolutidae [38]. One group includes our focus species *Convolutriloba macropyga* and the better known *Symsagittifera roscoffensis*; photosymbionts in this group are always green algae, with the exception of *Convoluta convoluta*, which bears diatoms as endosymbionts [44, Additional File 3: Table S1]. In the other group, comprising *Waminoa* and *Amphiscolops*, the endosymbionts are dinoflagellates; the symbiotic status or the nature of symbionts in some acoel species included in our analysis is unknown (Fig. 1A), but given that all other known *Waminoa* or *Amphiscolops* species harbour dinoflagellates (Additional File 3: Table S1), it is safe to assume they harbour dinoflagellates as symbionts, too. Interestingly, some *Amphiscolops* species can simultaneously host dinoflagellates and green algae [38, 45] (Additional File 3: Table S1). In Japan, Riewluang and Wakeman [196] recently found a novel group of photosymbiotic acoels outside of Convolutidae, as a sister group to Mecynostomidae. These species were not included in our sources for phylogeny [102, 103] and are therefore not shown in our analysis, but a third independent occurrence of photosymbiosis remains possible.

Among the immune genes usually conserved in metazoans, there are some pattern recognition receptors (PRRs) and genes belonging to signalling pathways, such as the complement system and the Toll pathway [29] (Fig. 1B). We searched for predicted proteins with a domain structure corresponding to known PRRs in Xenacoelomorpha and found C-lectins, as well as scavenger receptors of class B and E. Toll-like receptors (TLRs) are considered lost in xenacoelomorphs [111]; we only recover them in the nemertodermatid *Nemertoderma westbladi*, a species not included in previous analyses [111]. A more in-depth investigation would be required to understand if *N. westbladi* sequences are homologous to other metazoan TLRs or if they have evolved de novo, for example from TIR-only or LRR-only containing proteins. NOD-like receptors (NLRs) are present in all investigated acoels, but *Praesagittifera naikaiensis*; they could not be found in *Xenoturbella* and nemertodermatids.

As for genes belonging to the complement system, they are progressively lost in xenacoelomorphs and completely missing from acoels. In fact, C3—the pathway central component—and factor B are absent from xenacoelomorphs and complement receptors 1/2 are only present in non-acoel xenacoelomorphs (Fig. 1B). The other signalling pathway we investigated, the Toll pathway, also seems completely lost in acoels and only traces can be found in other xenacoelomorphs (Fig. 1B). The activators of the pathway, TLRs, are only recovered in one of the two nemertodermatid species investigated, as discussed above. The transcription factor usually activated by the signalling cascade, NFκB, is only convincingly detected in the other nemertodermatid species, *Meara stichopi*. Predicted proteins with MyD88 domain structure (TIR and DD) are only found in non-acoel xenacoelomorphs. Upon phylogenetic analyses, these sequences could be more closely related to human TIRAP (TIR-domain containing adaptor protein) than to other metazoan MyD88s (Additional File 1: Fig. S4). TIRAP is also involved in the Toll signalling pathway, but is thought to be present only in chordates [197, 198]. Therefore, if a MyD88-like adaptor protein exists in xenacoelomorphs, it cannot be found in acoels, and it diverged consistently from other metazoans’ MyD88. Our data thus suggests that a canonical metazoan Toll signalling pathway is lost in acoels and possibly in other xenacoelomorphs, too.

**Fig. 8:**
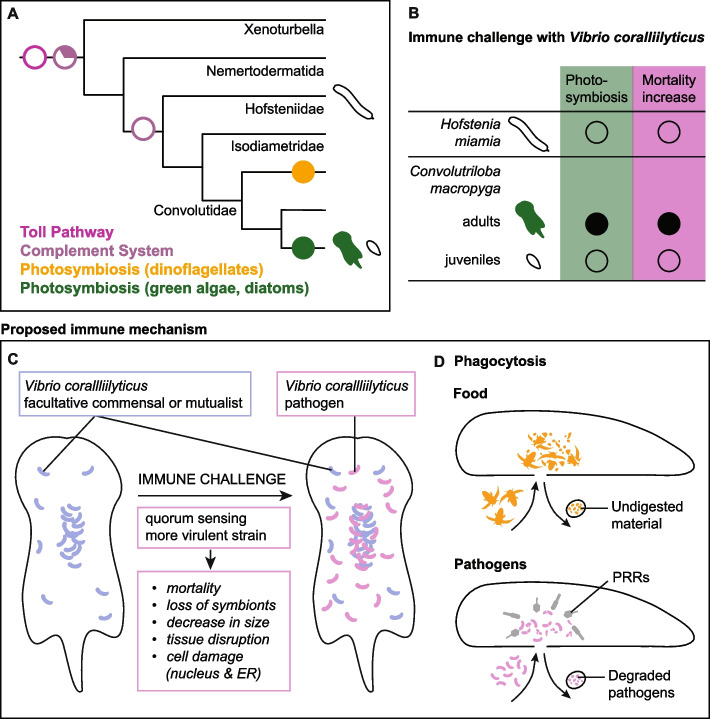
Summary of findings and working model. **A** Evolutionary scenario of immunity and photosymbiosis in Xenacoelomorpha: loss of immune signalling pathways preceded the occurrence of photosymbiosis. Full circles show gain of photosymbiosis, and empty or partially empty circles show loss of Toll and complement pathways. **B** Correlation of photosymbiosis in acoels and increased mortality upon immune challenge. Full circles show presence of algal symbionts or mortality increase, and empty circles show their absence. **C** Immune challenges of *Convolutriloba macropyga* with *Vibrio coralliilyticus*: proposed transition from commensal or mutualist to pathogen; findings of this study in italic. **D** Hypothesis of a common route for food digestion and pathogen clearance in acoels, based on our findings

The PRRs we investigated are known or suspected to interact with photosymbiotic endosymbionts in cnidarians [1, 19, 27], and in gastropod molluscs [117, 199]. Their conservation in acoels, with the exception of TLRs, prompts future investigations into their role in the interaction between acoels and their photosynthetic endosymbionts, too. The complement system and toll pathway are negatively associated with symbiosis in cnidarians: they are either downregulated in symbiotic animals or tissues, or upregulated during bleaching and loss of symbionts [27, 121, 141, 200, 201]. The loss of these signalling pathways precedes the evolution of photosymbiosis in acoels (Fig. 8A). It could have favoured the occurrence of photosymbiosis, especially considering that the endosymbionts are not confined to a specific tissue, as in cnidarians, but dispersed throughout the animal’s body instead [37]. Given that Acoela—and Convolutidae in particular—are fast-evolving clades [102, 202], major genomic changes outside of the immune gene repertoire are to be expected. Further genomic specialisations linked to photosymbiosis will likely be uncovered by future research [1].

### Coral-bleaching agent *Vibrio coralliilyticus* causes mortality and dysbiosis in photosymbiotic acoels

In corals, *Vibrio coralliilyticus* is an opportunistic pathogen, associated to bleaching, tissue lysis, and mortality [25, 166, 168, 178]. It also causes mortality in a variety of marine animals, from bivalves and crustaceans to sea urchin and fish [156, 158–160, 162]. Our data shows that *V. coralliilyticus* has the potential to be virulent to photosymbiotic acoels, too: (i) Mortality in *Convolutriloba macropyga* adults increases upon exposure to *V. coralliilyticus* (Fig. 2A, C, Fig. 8B); (ii) *V. coralliilyticus* 16S rRNA is present in immune challenged animals, but also in controls (Fig. 3A, B, Additional File 1: Fig. S8); (ii) heat-inactivated bacteria do not decrease survival (Additional File 1: Fig. S6A), confirming an active role of bacteria in causing mortality. Tissue degradation (Fig. 5) also resembles the effects of *V. coralliilyticus* on corals [154, 166, 168] and sea anemones [163]. The presence of *Vibrio* rRNA in some control samples suggests that these bacteria are mutualists or commensals commonly present in *C. macropyga*. However, *Vibrio* rRNA was not present in all individuals and we have not observed any alive bacteria by transmission electron microscopy (TEM); a possible explanation is a transient presence of *V. coralliilyticus* in the normal microbiome of *C. macropyga* and its routine degradation by the host, which would also explain why *Vibrio* rRNA varies in presence and localization between individuals [203, 204]. Moreover, it should be noted that TEM does not allow a comprehensive study and an inconspicuous number of bacteria could simply have been missing in the sections or individuals observed. The higher virulence of experimentally added *V. coralliilyticus*—and consequential *C. macropyga* mortality—could be due to quorum sensing [205, 206] or to variation between strains [207] (Fig. 8C); the latter interpretation is supported by the difference in bands obtained by PCR amplification of *V. coralliilyticus* genes from *V. coralliilyticus* and *C. macropyga* cDNA (Additional File 1: Fig. S8).

We do not know if these laboratory observations correspond to conditions in the wild. Data on *V. coralliilyticus* geographical distribution are available [155, 207], although *C. macropyga* localization in the wild is still unknown [37]. Moreover, *V. coralliilyticus* virulence in corals is only observed at higher temperatures [155, 163, 166, 168], with mortality only happening above 25 °C or 27 °C and bleaching above 24 °C [166, 168]. As for sea anemone, mortality—but not dysbiosis—is reported in *Exaiptasia diaphana* exposed to *V. coralliilyticus* at 30 °C and not at 25 °C by one study [208] and at 22 °C by another [163]. Therefore, our results for mortality and dysbiosis in *C. macropyga*—obtained at 26 °C—could also vary with temperature.

It should be noted that, in cnidarians exposed to *V. coralliilyticus*, tissue loss is detectable only after 10–12 days and death after 15–21 days [166, 168]. In contrast, an increase in mortality of *C. macropyga* can already be detected at 2 days of bacterial exposure. Thus, these acoels could be used as an early warning system for coral diseases in tropical aquaria or coral reef monitoring, in a similar way to rosebushes for vineyards.

However, mortality does not increase when *V. coralliilyticus* exposure happens in the absence of photosymbiosis, i.e. in aposymbiotic *C. macropyga* juveniles and aposymbiotic acoel species *Hofstenia miamia* (Fig. 8B)*.* Thus, the presence of photosynthetic endosymbionts seems linked to a higher susceptibility to pathogens. This could be due to (i) a weakened immune status needed to maintain symbiosis, as suggested for corals [21] or (ii) host mortality caused by a detrimental effect of *V. coralliilyticus* on the endosymbionts rather than on the host itself; direct damages to symbiotic dinoflagellates were also observed in corals infected by *Vibrio* [209, 210].

To our knowledge, there is no study directly comparing mortality in symbiotic and aposymbiotic cnidarians upon *V. coralliilyticus* exposure. However, different strains of *Symbiodinium* endosymbionts correlate with differences in disease resistance [211], suggesting that photosymbionts can affect a host’s ability of facing infections. In the sea anemone *Exaiptasia diaphana*, survival upon infection with bacteria *Pseudomonas aeruginosa* or *Serratia marcescens* is higher in aposymbiotic individuals compared to symbiotic ones, but this effect is reversed in the presence of starvation [212]. In the scyphozoan *Cassiopea xamachana* challenged with *S. marcescens*, survival is also higher in aposymbiotic than in symbiotic polyps [213]*.* Given a similar effect of photosymbiosis on disease susceptibility in independent photosymbiotic systems, this may be due to intrinsic features of photosymbiosis.

Since we compared *V. coralliilyticus* effects on mortality in different species or ontogenetic stages, these factors could be more relevant than the presence of photosymbiosis. For example, a meta-analysis in bilaterians with separate sexes showed that increased mortality upon immune challenges is associated with adult, but not juvenile stages [214]. An alternative explanation for *H. miamia* higher survival upon immune challenge could be its stronger regeneration capacity: although whole body regeneration in *H. miamia* and *Convolutriloba* species has been described as comparable [215], damaged *Convolutriloba* species release a toxin that can cause the death of the animal itself and surrounding individuals [37, 40, 41]. Since signs of tissue disruption upon bacterial exposure are seen both in *H. miamia* and *C. macropyga* (Fig. 5, Additional File 2: Video S1), *H. miamia* could be more efficient at repairing damage than *C. macropyga*.

The reduced number of algal symbionts per animal cell suggests that photosymbiosis in* C. macropyga* breaks down upon *V. coralliilyticus* exposure (Fig. 4, Additional File 4: Tables S3). While coral dysbiosis is usually clearly recognisable by white areas, in the scyphozoan *Cassiopea andromeda*, elevated sea temperatures cause ‘invisible bleaching’, a decrease in symbiont density and chlorophyll activity without any noticeable pigmentation change [216]. A similar phenomenon could also be at play in sea anemones challenged with *V. coralliilyticus*, where no bleaching is visible [163]. The shorter length of *C. macropyga* exposed to *V. coralliilyticus* could be linked to a loss of nutrient apport from the symbionts. However, in *Convolutriloba retrogemma* exposed to elevated temperatures, a decrease in symbiont numbers was observed prior to mortality, without any changes in animal length [42]. Therefore, symbiont loss and mortality can happen without a size decrease in *Convolutriloba* species. This suggests that either responses to heat stress and immune challenges differ, or that shrinking in immune challenged individuals is not due to symbiosis disruption.

### Immune responses in acoels: novel molecular mechanisms and a potential common route for food and pathogens

So far, there have been no studies on acoel immune responses to pathogens. Given the lack of usually highly conserved immune pathway genes (Fig. 8A), non-canonical immune mechanisms may play a role. We initially checked for a behavioural response to infections: terminal investment in reproduction. An increase in reproduction, in lieu of a costly immune response, could be linked to a reduced immune gene repertoire and immune response in pea aphids [217–219]. However, progeny release is unaffected in immune-challenged *Convolutriloba macropyga*, so we rejected the hypothesis of terminal investment.

Transmission electron microscopy data in immune-challenged *C. macropyga* shows a degradation of the pathogens in the digestive parenchyma (Fig. 3C–G), where phagocytosis is used for food digestion [220]. In situ hybridisation also shows *Vibrio* rRNA and host PRRs (pattern recognition receptor) expression inside or around the digestive system in most cases (Fig. 3A,B, Additional File 1: Fig. S7, Fig. 6). The expulsion of a tissue ball by immune-challenged *H. miamia* (Additional File 2: Video S1) suggests the disposal of digested pathogens through the mouth. However, we did not observe a similar behaviour in *C. macropyga*, which could be linked to its less efficient response to *Vibrio* or to technical limitations*.* Lacking an anus, acoels routinely eliminate undigested food through the mouth [220]. Based on our data, we suggest a similar route for food uptake and immune responses (Fig. 8D): pathogens likely enter the animal through the mouth; they are detected by PRRs in the digestive system, where they are destroyed by phagocytosis; finally, the remains are disposed through the mouth. Whether the same cells are responsible for food and pathogen phagocytosis is unclear. Further research could not only confirm this working hypothesis and better characterise immune responses in acoels, but also help understanding the evolutionary origin of phagocytic cells in animals [221].

A molecular response to pathogen exposure seems inconspicuous. First, in situ hybridisation shows that the PRRs identified in the predicted proteomes are expressed by the animal, but their expression domains vary greatly both in controls and in animals exposed to *Vibrio* (Fig. 6). Despite a great individual variability, we identified some regions that are more likely to be involved in the first stage of immune responses in acoels (Fig. 6H). The mouth and digestive system are likely the areas of first encounter and elimination of microbes, as discussed above; the nervous system and reproductive structures (gonads and asexual buds) would be essential areas to protect from pathogens. PRR expression in the digestive and nervous system has also been described for nemertean, nematode, and crustacean C-lectins [29, 222, 223], crustacean and mammalian SRs type B [222, 224, 225], and leech and mammalian NLRs [226–228]. *C. macropyga* flank regions expressing PRRs could correspond to a previously undescribed structure important for responses to microbes. Given the high variability in *V. coralliilyticus* 16S rRNA in situ hybridisation, the expression domains of PRRs could also be linked to the presence of bacteria, but we did not manage to perform double in situ hybridisation to test this. Secondly, our transcriptomic analysis indicates downregulation of one putative C-lectin, but no differential expression of other PRRs upon immune challenge (Additional File 5: Table S5). PRRs are downregulated early in corals exposed to *V. coralliilyticus* upon thermal loading [229], while they peak early in nemerteans exposed to *Vibrio diazotrophicus* [29]; if the scenario is similar in *C. macropyga*, they could be back to control levels after 2 days of exposure, when our experiments were performed.

Moreover, in transcriptomic analysis*,* sample similarity correlates with batch rather than exposure status (Fig. 7A). This denotes a lack of substantial changes in gene expression upon *Vibrio* exposure, suggesting that molecular immune responses may be minor or highly variable, at least at 48 h of exposure.

Upon differential gene expression analysis (Fig. 7C, Additional File 5: Table S5), we find a small number of differentially expressed genes (DEGs) compared to *V. coralliilyticus* immune challenges in cnidarians [168, 229, 230] and sea urchins [231]. Given the decrease in DEG number over exposure time [229], our results may be linked to the longer exposure time (48 h) compared to other studies [168, 229–231]. The DEGs recovered are therefore unlikely to be involved in the acute short-term response to pathogens; they are rather linked to a continuous exposure and a medium-term immune status change.

Among the characterised DEGs, we observe significant up- and downregulation of sequences belonging to non-*Vibrio* bacteria. This suggests an interaction between a potential pathogen and the bacterial community within *C. macropyga*, as extensively studied in human and model organisms [232, 233]. The upregulated host transcripts we could characterise are serine dehydratase and transient receptor potential cation channel M. Serine dehydratase is an enzyme converting serine into pyruvate [234], the starting substrate for Krebs cycle [235]. Its upregulation could be linked to an increased energetic need following the reduction in nutrient-supplying endosymbionts. Carbonic anhydrase, which catalyses the conversion between CO_2_ and HCO_3_⁻, is important for photosynthesis and for photosymbiosis in anthozoans [236]. Its downregulation upon immune challenge could also be due to symbiosis breakdown. Transient receptor potential cation channels are involved in thermosensation and other sensory functions [237, 238] and are expressed in putative sensory neurons in the acoel *Aphanostoma pulchra* [239]. Among the downregulated genes, we find serine proteases: protein-cleaving enzymes with a variety of functions, which have been associated with several immune responses in insects and humans [195, 240–242]. Downregulation of dynein regulatory complex subunit, a gene involved in ciliary function [243], and of adhesion molecules—related to trophinin and mucins [244], nidogens [245], and adhesion plaque protein [246]—could be linked to changes in cell motility. To summarise, changes in host gene expression levels seem to relate to potential immune responses, sensory functions, cell motility or adhesion, and the response to symbiosis breakdown. However, many of the genes differentially expressed upon immune challenge are not orthologues of any characterised gene and do not present known conserved domains (Fig. 7C, Additional File 5: Table S5). Acoel-specific genes, as well as orthologues of still uncharacterised animal genes, seem to play a role in responses to bacterial exposure in acoels. Considering the unknown identity of these genes, the small significance of molecular responses, and the lack of changes in reproduction, novel immune strategies may be employed by acoels. These warrant further research, as well as other conserved immune mechanisms not addressed in this study, such as radical oxygen species, phenoloxidase, and melanization [31].

## Conclusions

Our study finds that Acoela, and to some degree the whole Xenacoelomorpha clade, lost immune pathway genes commonly conserved in animals. This loss preceded—and potentially favoured—the evolution of photosymbiosis, which happened at least twice independently in this clade. We show that exposure to the bacterium *Vibrio coralliilyticus* does not affect non-photosymbiotic acoels, but it increases mortality in the photosymbiotic acoel *Convolutriloba macropyga*. It also decreases the ratio between symbiotic algae and animal cells, however without visible bleaching. Phagocytosis in the digestive system seems to be involved in pathogen clearance. At a molecular level, we detected minimal changes in immune receptor expression patterns upon *Vibrio* exposure and most of the differentially expressed genes do not correspond to known animal genes, nor contain known conserved domains. This suggests the possibility of still unknown immune mechanisms in acoels. Acoels are therefore a promising model for uncovering new immune mechanisms and for investigating the coevolution of photosymbiosis and the immune system in comparison to cnidarians.

## Methods

### Immune gene searches

Immune genes were searched in predicted proteomes based on the presence of relevant domains and their order (Additional File 1: Fig. S1). The predicted proteomes were the following: *Xenoturbella bocki* (transcriptome SRX1343818 [247]), *Meara stichopi* (transcriptome SRX1343814 [248]), *Nemertoderma westbladi* (genome [249]), *Hofstenia miamia* (genome [250]), *Aphanostoma pulchra* (transcriptome SRX1343817 as *Isodiametra pulchra *[251]), *Amphiscolops* sp. (genome [252]), *Praesagittifera naikaiensis* (genome [46]), *Symsagittifera roscoffensis* (genome [253]), and *Convolutriloba macropyga* (genome [252] and transcriptome SRX1343815 [254]). Domains were identified according to the literature (see the “Results” section), as well as through searches of established protein sequences in the NCBI Conserved Domain Database, CDD [255–257]. Alignments for the domains were downloaded from CDD and HMMER v.3.3 [www.hmmer.org, 258] was used to build Hidden Markov Model profiles (*hmmbuild*), which were used to search the predicted proteomes (*hmmsearch*). The retrieved xenacoelomorph sequences were submitted to NCBI CD Batch Search (https://www.ncbi.nlm.nih.gov/Structure/bwrpsb/bwrpsb.cgi [256]) and a sequence was kept only if it contained the domains of interests (in the correct order if relevant—see the “Results” section). PRR sequences were considered present or absent based only on the domain structure, whereas for signalling pathway sequences we also performed phylogenetic analysis: the domain sequences identified by *hmmsearch* were aligned with MAFFT v7.526 [259, 260], AMAS was used to concatenate alignments [261], and iqtree2 v2.4.0 [262] to find the best partition and model [263, 264], as well as to build maximum likelihood trees, using UltraFast Bootstrap with 1000 replicates as support measure [265].

### Animals and bacteria

*Convolutriloba macropyga* Shannon & Achatz, 2007 [37] were kept in artificial sea water with salinity 36 ‰ (Classic Sea Salt by Tropic Marin, Dr. Biener GmbH, Wartenberg, Germany) at 26 °C with a cycle of 10 h darkness–14 h light, and they were fed twice a week with brine shrimp *Artemia*. Aposymbiotic *C. macropyga* juveniles were collected by isolating freshly laid egg clusters in clean artificial sea water to ensure no contact with potential symbionts. *Hofstenia miamia* Corrêa, 1960 [153] were kept in filtered artificial sea water salinity 36 ‰ (Red Sea Salt by Red Sea Fish Pharm LTD, Eilat, Israel) at room temperature in the dark, and they were fed twice a week with brine shrimp *Artemia* sp.

*Vibrio coralliilyticus* and *Priestia megaterium* were obtained from the Jena Microbial Research Collection of the Leibniz Institute for Natural Product Research and Infection Biology, Leibniz-HKI, Jena, Germany (Strain STH00823 and STI11342, respectively). They were both grown overnight at 30 ± 2 °C with 100 rpm shaking prior to use in immune challenges. The growth media were Difco Marine Broth 2216 (Becton, Dickinson, and Company, USA) for *V. coralliilyticus* and nutrient broth (DSMZ medium 1 [266]) for *P. megaterium*. A calibration curve was computed prior to immune challenge experiments as previously described [267] to correlate concentration in colony forming unit (CFU)/ml to optical density at 600 nm (OD600).

### Immune challenges

Immune challenges were performed on *C. macropyga* adults and juveniles (0–4 days after hatching) and *H. miamia* adults. Bacteria OD600 was measured on the spectrophotometer NanoPhotometer (Implen GmbH, Munich, Germany) and bacteria concentration was calculated as 6.6∙10^8^ CFUs/ml∙OD600 for V. coralliily*ticus* and as 1.9∙10^6^ CFUs/ml∙OD600 for *P. megaterium*, according to the calibration curves. Bacteria were collected by centrifugation (1000 g 5 min), resuspended in filtered artificial sea water (fASW), and serially diluted. Growing medium was also centrifuged, resuspended, and diluted in the same way, as a control medium for mortality caused by media components. For immune challenges with heat-killed bacteria, the bacteria were incubated 5 min at 95 °C with 200 rpm shaking after resuspension in fASW. Absence of movement in bacteria, as a proxy for activity, was confirmed by DIC microscopy (Axioscope 4, Carl Zeiss Microscopy GmbH, Jena, Germany – ×200 magnification). Animals were washed at least 3 times with fASW and isolated in 1 ml of fASW in individual wells of a 96-well plates (to avoid exposure to the toxins released upon death [37]). The appropriate volume (25–35 µl, constant within a batch) of resuspended growth medium or serially diluted bacteria—alive or heat-inactivated—was added to each well, to obtain a bacterial load of 0, 10^5^, or 10^6^ CFUs (control, low, and high load, respectively). The bacterial loads were chosen based on pilot experiments, as the ones causing a mortality higher than the control, but not 100% (Additional File 1: Fig. S10, *n* = 168, 24 per bacterial load). The plates were kept at animal culture conditions, but without feeding and medium exchange. They were monitored at 0, 1, 2, 4, 6, 24, and 48 h for the 2-day assays. *C. macropyga* juveniles were monitored every day for 10 days. For long-term immune challenges of *C. macropyga* adults, the animals were monitored every 2–5 days for 1 month (2 batches) or 3 months (1 batch). At each time point, animals were considered dead if not moving and showing tissue degradation, and the number of fully detached asexual progeny was counted for *C. macropyga* adults. All further processing of the animals (fixation for in situ hybridisation or electron microscopy, RNA extraction) was done on intact, alive animals. Pictures were taken with an IPhone 14 Pro (Apple Inc., Cupertino, CA, USA) mounted on a Leica M125 C (Leica Microsystems, Wetzlar, Germany).

### Dysbiosis quantification

Immune-challenged *C. macropyga* adults were relaxed for 5 min in a 1:1 mixture of fASW and 7% MgCl_2_ and then fixed at room temperature for 30 min in 4% paraformaldehyde diluted in fASW. They were washed 5× in phosphate saline buffer (PBS), incubated for 30 min in 2.5 µg/ml Hoechst 33,342 in PBS, then washed twice in PBS and mounted in 70% glycerol in PBS. Whole animals were imaged with an Axiocam 503 colour on an Axioscope 5 equipped with Differential Interference Contrast (DIC) (Carl Zeiss Microscopy GmbH, Jena, Germany). Chlorophyll B autofluorescence and Hoechst 33342 signal were imaged in a flat area between the mouth and the eyes with a LSM 980 confocal laser scanning microscope (Carl Zeiss Microscopy GmbH, Jena, Germany). The images were processed with a pipeline in JIPipe v5.2.0 [268]: for each channel, noise was reduced through a median filter, then brightness enhanced by dividing greyscale values by global maximum; for the algae autofluorescence channel, a difference of Gaussian was used as feature enhancer; then, algae and animal nuclei were binarized automatically with a global thresholding method (Otsu for the Hoechst channel and Huang for the algae autofluorescence channel); after a morphological hole filling for the algae and distance transform watershed for both channels, the region of interests (animal nuclei and algae) were detected and counted. The length of each individual was measured on preview images for each slide with Zeiss ZEN lite microscopy software v3.11, and its orientation (dorsal, ventral, lateral-dorsal) was determined based on DIC pictures.

### PCR amplification, gene cloning, and probe synthesis

Primers were designed with Primer Blast [269] for *C. macropyga* PRR genes identified, as well as for *V. coralliilyticus* 16S rRNA (NR_028014) and virulence factors (WP_006957348.1, WP_006959328.1, WP_006959836.1, WP_006960006.1, WP_006961218.1, WP_006961766.1, WP_006962231.1, WP_006962597.1, WP_141650583.1) [173] (Additional File 6: Table S6). For 16S rRNA primers, we selected a region that did not align to other non-*Vibrio* bacterial sequences retrieved with a BLASTn search [270]. Lack of aspecific amplification was checked with an in silico PCR tool [271] against *C. macropyga* transcriptome. Lack of aspecific hybridisation was checked in silico by BLASTing the amplicon against *C. macropyga* transcriptome within SequenceServer v2.2.0 (blastn, evalue 1e-05, sc-match 2, sc-mismatch −3, gap-open 5, gap-extend 2) [272, 273].

Fragments of the genes were amplified by PCR from cDNA libraries obtained with SuperScript™ III First-Strand Synthesis System (Thermo Fisher Scientific, Waltham, MA, USA) and run on a 1% agarose gel in TAE buffer (40 mM Tris, 20 mM acetic acid, 1 mM EDTA). For cloning, they were ligated into pGEM-T Easy vectors (Promega, Madison, WI, USA) and transformed into competent *Escherichia coli* cells according to the manufacturer’s instructions. Plasmids with an insert of the expected size were extracted with Plasmid Mini-Prep Kit (Jena Bioscience, Jena, Germany) and the inserts were sequenced at Eurofins Genomics Europe Shared Services GmbH (Ebersberg, Germany). Labelled antisense RNA probes were transcribed from linearized DNA using digoxigenin-11-UTP (Roche, Basel, Switzerland) with the MEGAscript T7 or SP6 kit (Thermo Fisher Scientific, Waltham, MA, USA). Probes for some scavenger receptor and C-lectin genes could not be obtained.

### Whole-mount in situ hybridisation

Immune challenged *C. macropyga* adults were relaxed and fixed as explained above. The samples were washed 5 times in PTw (1× phosphate saline buffer with 0.1% Tween-20 detergent) and stored at −20 °C in methanol until further processing. Whole-mount in situ hybridisation was carried out as previously described [274] with the following modifications: probe concentration was 1 ng/µl and hybridisation temperature 65 °C. Samples were then washed with ethanol and rehydrated in an increasing series of PTw in ethanol, before mounting them in 70% glycerol in PTw. They were imaged using an Axiocam 503 colour on an Axioscope 5 with DIC (Carl Zeiss Microscopy GmbH, Jena, Germany). As a negative control, the protocol was performed without adding any probe (Additional File 1: Fig. S7).

### Transmission electron microscopy

Immune-challenged *C. macropyga* adults were relaxed in 1:1 solution of 7% MgCl_2_ and fASW and then fixed with a mixture of 0.4% paraformaldehyde and 2.5% glutaraldehyde in 50 mM cacodylate buffer containing 23 mg/ml NaCl and 2.5 g/ml MgCl_2_. After washing in 50 mM cacodylate buffer with 23 mg/ml NaCl, the samples were post-fixed in 1% OsO_4_ in 0.1 M cacodylate buffer, washed in distilled water, and dehydrated in an increasing series of ethanol concentrations and isopropanol. They were then infiltrated in a mixture of isopropanol and Spurr resin, before embedding them in pure Spurr resin for 1 to 2 days at 60°C. *V. coralliilyticus* from liquid cultures were resuspended in fASW and subsequently processed as described above, being pelleted by centrifugation at every step (1000 g, 5 min). After post-fixation, they were rehydrated to bidistilled water, pelleted by centrifugation (13 000 g for 30 min), and embedded in 2% agarose, before proceeding to dehydration, infiltration, and embedding.

Ultrathin sections (70 nm) were prepared with a Leica EM UC 7 ultramicrotome (Leica Microsystems, Wetzlar, Germany) from two samples per condition (control, low dose, high dose) collected at 48 h of exposure (he), one sectioned longitudinally and one transversely. In addition, transverse sections were obtained from one control sample at 1 he, two high-bacterial-load sample at 1 he, and two high-bacterial-load sample at 2 he. Ultrathin sections were mounted on slot grids and contrasted with UranyLess EM Stain (Electron Microscopy Sciences, Hatfield, PA, USA) and 3% lead citrate; the incubation was 3 min and 2 min, respectively. They were examined with a Tecnai 12 transmission electron microscope (FEI Deutschland GmbH, Dreieich, Germany), equipped with a digital camera (TEMCAM FX416, TVIPS, Gauting, Germany).

### Transcriptomics and differential gene expression analysis

Bulk RNA from 2-day immune-challenged *C. macropyga* adults (controls and low *V. coralliilyticus* load, 4 biological replicates of 5–10 individuals each) was extracted using TRIzol (Invitrogen, Thermo Fisher Scientific, Waltham, MA, USA) and 1-bromo-3-chloropropane (fisher scientific, Thermo Fisher Scientific, Waltham, MA, USA). The NEBNext Ultra II directional mRNA kit (New England Biolabs, Ipswich, MA, USA) was used to prepare libraries and sequencing on Illumina NovaSeq6000 platform resulted in 567.3 million total reads (average read length 151 bp), available at NCBI (BioProject PRJNA1332086 [275]). A transcriptome was assembled de novo with Trinity v2.15.1 [276] after quality control with fastQC (https://www.bioinformatics.babraham.ac.uk/projects/fastqc/) and trimming with fastp [277], including additional reads from *C. macropyga* juveniles processed as above (5 biological replicates, 340.4 million reads of 151 bp average length) [275]. Salmon v0.13.1 [278] was used to index the transcriptome and quantify transcript levels. Transcript level estimates, imported into R v4.3.0 [279] with the *tximport* package (design = replicate + condition, to account for paired samples) [280, 281], were analysed with the *DESeq2* package [281]: principal component analysis after variance stabilising transformation was performed with *plotPCA* and differential gene expression analysis with *DESeq*. Significantly differentially expressed genes (FDR-adjusted *p*-value < 0.01) were characterised by blastx search (https://blast.ncbi.nlm.nih.gov) [273] against NCBI non-redundant protein sequences database (if default parameters did not yield any hits, compositional adjustment was set to ‘no adjustment’ and filter low complexity regions to ‘unchecked’), as well as by Conserved Domain search (https://www.ncbi.nlm.nih.gov/Structure/cdd/wrpsb.cgi) [255, 257, 282]. If no hits or only uncharacterised proteins were retrieved, the sequences were searched against xenacoelomorph-predicted proteomes used for immune gene searches with blastx v12.6.0+ [283].

### Statistical analyses and figures

Statistical analyses were carried out with R statistical software (v 4.3.0) [279]. Code and input data are available on GitHub [284]. Graphs and phylogenetic trees were produced in R v4.3.0 (Packages: *ggplot2* [285], ggtree [286], and *treeio* [287]). Figures were assembled with Adobe Illustrator. If necessary, contrast and brightness were adjusted on the whole picture with Adobe Photoshop or Fiji [288].

Survival data over time was fitted to a Cox proportional hazards mixed-effect model, with bacterial load and initial damage (accidentally caused during manipulation) as fixed explanatory variables and batch and individuals as random explanatory variables for the intercept (Additional File 4: Table S2, *coxme* package [289]). The maximal model was simplified by sequentially eliminating all the non-significant terms and interactions, while keeping each simplified model only if equivalent to the previous more complex model (anova, *α* = 0.05), until a minimal adequate model was found [290, 291]. The significance of fixed explanatory variables was tested with a type II Anova (*car* package [292]). When relevant, post hoc pairwise comparisons of the estimated marginal means between samples with different bacterial loads were conducted, with *p* value adjusted with the Bonferroni method (*emmeans* package [293]). Survival curves were plotted with ggsurvplot (*survminer* package [294]), considering fixed effects only.

For dysbiosis quantification, the ratio between the number of algae and animal nuclei was calculated for each sample from the JIPipe pipeline output. It was fitted to a linear model with bacterial load, sample length, and sample orientation (dorsal, ventral or lateral-dorsal) as interacting explanatory variables (Additional File 4: Table S3). The model was simplified as described above. Post hoc Tukey HSD contrasts were carried out to compare bacterial loads [295]. Animal length was fitted to a linear model (Length ~ Bacterial.Load) and the significance of bacterial load was tested with a type II Anova, followed by post hoc Tukey HSD tests (*glht* function from the *multcomp* package [295]).

Asexual offspring production was fitted to a generalised linear mixed model (*glmmTMB* package [296]), with bacterial load and presence of a bud as fixed effect explanatory variables and batch as random explanatory variable for the intercept (Additional File 4: Table S4). Data for day 1 and day 2 of exposure were analysed separately to avoid temporal pseudoreplication due to repeated measurements [290]. Different family functions were compared using Akaike’s information criterion (AIC) to select the more appropriate one [290, 297, 298]. Simplification of the maximal model was carried out as described for survival data.

## Supplementary Information

Additional File 1: Fig. S1-S10.

Additional File 2: Video S1. Hofstenia miamia expelling a sphere of tissue upon immune challenge.

Additional File 3: Table S1. Presence and type of photosynthetic endosymbionts.

Additional File 4: Table S2-S4. Survival, dysbiosis, and reproduction statistics.

Additional File 5: Table S5. Differentially expressed genes between* C. macropyga* adults immune challenged with a low *V. coralliilyticus* load and controls at 2 days of exposure.

Additional File 6: Table S6. Primers for PCR amplification.

Additional File 7: Data S1 – Xenacoelomorpha SRs class B.

Additional File 8: Data S2 – Xenacoelomorpha SRs class E.

Additional File 9: Data S3 – Xenacoelomorpha SRs class I.

Additional File 10: Data S4 – Xenacoelomorpha C-type lectins.

Additional File 11: Data S5 – Xenacoelomorpha TLRs.

Additional File 12: Data S6 – Xenacoelomorpha NLRs.

Additional File 13: Data S7 – Xenacoelomorpha candidate C3.

Additional File 14: Data S8 – Metazoa C3 and outgroups.

Additional File 15: Data S9 – Xenacoelomorpha candidate CR1/2.

Additional File 16: Data S10 – Metazoa CR1/2 and outgroups.

Additional File 17: Data S11 – Xenacoelomorpha candidate MyD88.

Additional File 18: Data S12 – Metazoa MyD88 and outgroups.

Additional File 19: Data S13 – Xenacoelomorpha candidate NFkB.

Additional File 20: Data S14 – Metazoa NFkB and outgroups.

Additional File 21: Data S15 – Significantly differentially expressed genes.

## Data Availability

All relevant data generated or analysed during this study are either included in this published article and its supplementary information files or available at NCBI databases under the mentioned accession numbers. The transcriptomic data generated are available at NCBI SRA database (BioProject PRJNA1332086, SRAs: SRX30605405, SRX30605406, SRX306054110, SRX30605411, SRX30605412, SRX30605413, SRX30605414, SRX30605415). Custom scripts and their input data for gene search and statistical analysis are available at GitHub https://github.com/francescapinton/acoel-immune-signalling-pathways-immune-challenges (10.5281/zenodo.17535040).

## Abbreviations

A2M: α2-macroglobulin domain
AM-R: alpha-macroglobulin binding domain
ANATO: anaphylatoxin domain
b: bud
BD: bacterial debris
Bf: factor B
C-LEC: C-type lectin
C345C: C-terminal domain specific to C3, C4, and C5
CCP: complement control protein domain
CD36: cluster of differentiation 36
CFU: colony-forming unit
CLECT: C-type lectin domain
CR: complement receptor
d: digestive system
DD: Death Domain
DEG: differentially expressed gene
DIC: differential interference contrast
EDTA: Ethylenediaminetetraacetic acid
ER: endoplasmic reticulum
f: flank
fASW: filtered artificial sea water
FDR: False Discovery Rate
g: gonads
HR: hazard ratio
IRAK: Interleukin-1 receptor-associated kinases
L: lysosome
LB: lipofuscin body
LRR: Leucine-rich repeat
m: mouth
MG: macroglobulin domain
MyD88: Myeloid differentiation factor 88
n: nervous system
n/g: nervous system or gonads
NFAT: Nuclear Factor of Activated T-cells
NFκB: Nuclear Factor-κB
NLR: NOD-like receptor
OM: outer membrane
PC: principal component
PCR: polymerase chain reaction
PM: plasma membrane
PRR: pathogen recognition receptor
PS: periplasmic space
PTw: Phosphate Saline Buffer with Tween-20
RHD_bind: Rel homology DNA-binding domain
RHD_dimer: Rel homology dimerization domain (immunoglobulin-plexin-transcription domain)
SR: scavenger receptor
SRCR: scavenger receptor cysteine rich domain
TAE: Tris, acetic acid, EDTA
TEM: transmission electron microscopy
TIR: Toll/IL-1 receptor domain
TLR: Toll-like receptor
TM: transmembrane region
TrypSP: trypsin-like serine protease domain
Vc: Vibrio coralliilyticus
vWA: von Willebrand factor type A domain

## References

[1] Melo Clavijo J, Donath A, Serôdio J, Christa G. Polymorphic adaptations in metazoans to establish and maintain photosymbioses: evolution of photosymbiosis. Biol Rev. 2018;93(4):2006–20. 10.1111/brv.12430. 29808579 10.1111/brv.12430

[2] Cowen R. The Role of Algal Symbiosis in Reefs through Time. Palaios. 1988;3(2):221. 10.2307/3514532.

[3] Venn AA, Loram JE, Douglas AE. Photosynthetic symbioses in animals. J Exp Bot. 2008;59(5):1069–80. 10.1093/jxb/erm328. 18267943 10.1093/jxb/erm328

[4] Yonge CM. Origin and nature of the association between invertebrates and unicellular algæ. Nature. 1934;134(3375):12–5. 10.1038/134012a0.

[5] Harvell CD, Mitchell CE, Ward JR, Altizer S, Dobson AP, Ostfeld RS, et al. Climate warming and disease risks for terrestrial and marine biota. Science. 2002;296(5576):2158–62. 10.1126/science.1063699. 12077394 10.1126/science.1063699

[6] Hughes TP, Baird AH, Bellwood DR, Card M, Connolly SR, Folke C, et al. Climate change, human impacts, and the resilience of coral reefs. Science. 2003;301(5635):929–33. 10.1126/science.1085046. 12920289 10.1126/science.1085046

[7] Roth MS. The engine of the reef: photobiology of the coral-algal symbiosis. Front Microbiol. 2014;5(422). 10.3389/fmicb.2014.00422. 10.3389/fmicb.2014.00422 PMC4141621 25202301

[8] Stanley GD, Lipps JH. Photosymbiosis: The Driving Force for Reef Success and Failure. Paleontol Soc Pap. 2011;17:33–59. 10.1017/S1089332600002436.

[9] Van Woesik R, Shlesinger T, Grottoli AG, Toonen RJ, Vega Thurber R, Warner ME, et al. Coral‐bleaching responses to climate change across biological scales. Glob Change Biol. 2022;28(14):4229–50. 10.1111/gcb.16192. 10.1111/gcb.16192 PMC9545801 35475552

[10] Weis VM. Cell biology of coral symbiosis: foundational study can inform solutions to the coral reef crisis. Integr Comp Biol. 2019;59(4):845–55. 10.1093/icb/icz067. 31150064 10.1093/icb/icz067

[11] Anthony KRN, Kline DI, Diaz-Pulido G, Dove S, Hoegh-Guldberg O. Ocean acidification causes bleaching and productivity loss in coral reef builders. Proc Natl Acad Sci U S A. 2008;105(45):17442–6. 10.1073/pnas.0804478105. 18988740 10.1073/pnas.0804478105 PMC2580748

[12] Bhagooli R, Hidaka M. Photoinhibition, bleaching susceptibility and mortality in two scleractinian corals, *Platygyra ryukyuensis* and *Stylophora pistillata*, in response to thermal and light stresses. Comp Biochem Physiol A Mol Integr Physiol. 2004;137(3):547–55. 10.1016/j.cbpb.2003.11.008. 15123191 10.1016/j.cbpb.2003.11.008

[13] Brown BE. Coral bleaching: causes and consequences. Coral Reefs. 1997;16:S129-38. 10.1007/s003380050249.

[14] Hoegh-Guldberg O, Smith GJ. The effect of sudden changes in temperature, light and salinity on the population density and export of zooxanthellae from the reef corals *Stylophora pistillata* Esper and *Seriatopora hystrix* Dana. J Exp Mar Biol Ecol. 1989;129(3):279–303. 10.1016/0022-0981(89)90109-3.

[15] Jokiel PL, Coles SL. Response of Hawaiian and other Indo-Pacific reef corals to elevated temperature. Coral Reefs. 1990;8(4):155–62. 10.1007/BF00265006.

[16] Lesser MP. Oxidative stress causes coral bleaching during exposure to elevated temperatures. Coral Reefs. 1997;16(3):187–92. 10.1007/s003380050073.

[17] Lesser MP, Stochaj WR, Tapley DW, Shick JM. Bleaching in coral reef anthozoans: effects of irradiance, ultraviolet radiation, and temperature on the activities of protective enzymes against active oxygen. Coral Reefs. 1990;8(4):225–32. 10.1007/BF00265015.

[18] Weis VM. Cellular mechanisms of Cnidarian bleaching: stress causes the collapse of symbiosis. J Exp Biol. 2008;211(19):3059–66. 10.1242/jeb.009597. 18805804 10.1242/jeb.009597

[19] Davy SK, Allemand D, Weis VM. Cell biology of Cnidarian-Dinoflagellate symbiosis. Microbiol Mol Biol Rev. 2012;76(2):229–61. 10.1128/MMBR.05014-11. 22688813 10.1128/MMBR.05014-11 PMC3372257

[20] Bieri T, Onishi M, Xiang T, Grossman AR, Pringle JR. Relative contributions of various cellular mechanisms to loss of algae during cnidarian bleaching. PLoS One. 2016;11(4):e0152693. 10.1371/journal.pone.0152693. 27119147 10.1371/journal.pone.0152693 PMC4847765

[21] Merselis DG, Lirman D, Rodriguez-Lanetty M. Symbiotic immuno-suppression: is disease susceptibility the price of bleaching resistance? PeerJ. 2018;17(6):e4494. 10.7717/peerj.4494. 10.7717/peerj.4494 PMC5909685 29682405

[22] Muller EM, Rogers CS, Spitzack AS, Van Woesik R. Bleaching increases likelihood of disease on *Acropora palmata* (Lamarck) in Hawksnest Bay, St John, US Virgin Islands. Coral Reefs. 2008;27(1):191–5. 10.1007/s00338-007-0310-2.

[23] Muller EM, Bartels E, Baums IB. Bleaching causes loss of disease resistance within the threatened coral species *Acropora cervicornis*. Elife. 2018;7:e35066. 10.7554/eLife.35066. 30203745 10.7554/eLife.35066 PMC6133546

[24] Kushmaro A, Loya Y, Fine M, Rosenberg E. Bacterial infection and coral bleaching. Nature. 1996;380(6573):396–396. 10.1038/380396a0.

[25] Rosenberg E, Koren O, Reshef L, Efrony R, Zilber-Rosenberg I. The role of microorganisms in coral health, disease and evolution. Nat Rev Microbiol. 2007;5(5):355–62. 10.1038/nrmicro1635. 17384666 10.1038/nrmicro1635

[26] Rosenberg E, Kushmaro A, Kramarsky-Winter E, Banin E, Yossi L. The role of microorganisms in coral bleaching. ISME J. 2009;3(2):139–46. 10.1038/ismej.2008.104. 19005495 10.1038/ismej.2008.104

[27] Mansfield KM, Gilmore TD. Innate immunity and cnidarian-Symbiodiniaceae mutualism. Dev Comp Immunol. 2019;90:199–209. 10.1016/j.dci.2018.09.020. 30268783 10.1016/j.dci.2018.09.020

[28] Iwama RE, Moran Y. Origins and diversification of animal innate immune responses against viral infections. Nat Ecol Evol. 2023;7(2):182–93. 10.1038/s41559-022-01951-4. 36635343 10.1038/s41559-022-01951-4

[29] Orús-Alcalde A, Børve A, Hejnol A. The localization of Toll and Imd pathway and complement system components and their response to *Vibrio* infection in the nemertean *Lineus ruber*. BMC Biol. 2023;21(1):7. 10.1186/s12915-022-01482-1. 36635688 10.1186/s12915-022-01482-1 PMC9835746

[30] Palmer CV, Traylor-Knowles N. Towards an integrated network of coral immune mechanisms. Proc R Soc Lond B Biol Sci. 2012;279(1745):4106–14. 10.1098/rspb.2012.1477. 10.1098/rspb.2012.1477 PMC3441085 22896649

[31] Rathinam RB, Acharya A, Robina AJ, Banu H, Tripathi G. The immune system of marine invertebrates: earliest adaptation of animals. Comp Immunol Rep. 2024;7:200163. 10.1016/j.cirep.2024.200163.

[32] McKenna V, Archibald JM, Beinart R, Dawson MN, Hentschel U, Keeling PJ, et al. The aquatic symbiosis genomics project: probing the evolution of symbiosis across the tree of life. Wellcome Open Res. 2021;6:254. 10.12688/wellcomeopenres.17222.1. 40438199 10.12688/wellcomeopenres.17222.2 PMC12117321

[33] Achatz JG, Chiodin M, Salvenmoser W, Tyler S, Martinez P. The Acoela: on their kind and kinships, especially with nemertodermatids and xenoturbellids (Bilateria *incertae sedis*). Org Divers Evol. 2013;13(2):267–86. 10.1007/s13127-012-0112-4. 24098090 10.1007/s13127-012-0112-4 PMC3789126

[34] Bourlat SJ, Hejnol A. Acoels. Curr Biol. 2009;19(7):R279-80. 10.1016/j.cub.2009.02.045. 19368867 10.1016/j.cub.2009.02.045

[35] Haszprunar G. Review of data for a morphological look on Xenacoelomorpha (Bilateria *incertae sedis*). Org Divers Evol. 2016;16(2):363–89. 10.1007/s13127-015-0249-z.

[36] McCoy AM, Balzer I. Algal Symbiosis in Flatworms. In: Seckbach J, editor. Symbiosis. Dordrecht: Kluwer Academic Publishers; 2001. p. 559–74. 10.1007/0-306-48173-1_35.

[37] Shannon T, Achatz JG. *Convolutriloba macropyga* sp. nov., an uncommonly fecund acoel (Acoelomorpha) discovered in tropical aquaria. Zootaxa. 2007;1525(1):1–17. 10.11646/zootaxa.1525.1.1.

[38] Achatz JG, Hooge M, Wallberg A, Jondelius U, Tyler S. Systematic revision of acoels with 9+0 sperm ultrastructure (Convolutida) and the influence of sexual conflict on morphology. J Zool Syst Evol Res. 2010;48(1):9–32. 10.1111/j.1439-0469.2009.00555.x.

[39] Cooper C, Clode PL, Thomson DP, Stat M. A flatworm from the genus *Waminoa* (Acoela: Convolutidae) associated with bleached corals in Western Australia. Zoolog Sci. 2015;32(5):465–73. 10.2108/zs140245. 26428725 10.2108/zs140245

[40] Delbeek JC, Sprung J. The reef aquarium. Coconut Grove, Florida: Ricordea Publishing; 1994.

[41] Lynford AH: Feature Article: Evaluation of Chemical Eradication Methods of Acoels (Acoelomorpha) From Marine Aquaria. 2023. https://coralrx.com/advanced-aquarist-evaluation-of-coral-rx/. Accessed 15 Apr 2025.

[42] Querido MC, Güth AZ, Garrido AG, Zilberberg C, Cardoso LP, Sumida PYG, et al. The photosymbiotic acoel *Convolutriloba retrogemma* (Xenacoelomorpha) is sensitive to thermal stress. J Exp Mar Biol Ecol. 2025;582:152079. 10.1016/j.jembe.2024.152079.

[43] Dupont S, Moya A, Bailly X. Stable photosymbiotic relationship under CO2-induced acidification in the acoel worm *Symsagittifera Roscoffensis*. PLoS One. 2012;7(1):e29568. 10.1371/journal.pone.0029568. 22253736 10.1371/journal.pone.0029568 PMC3253794

[44] Ax P, Apelt G. Die, “zooxanthellen” von convoluta convoluta (turbellaria acoela) entstehen aus diatomeen: Erster nachweis einer endosymbiose zwischen tieren und kieselalgen. Naturwissenschaften. 1965;52:444–6.

[45] Achatz JG. Convolutidae (Acoela) from the Andaman Sea. Zootaxa. 2008. 10.5281/ZENODO.274392.

[46] Arimoto A, Hikosaka-Katayama T, Hikosaka A, Tagawa K, Inoue T, Ueki T, et al. A draft nuclear-genome assembly of the acoel flatworm *Praesagittifera naikaiensis*. Gigascience. 2019. 10.1093/gigascience/giz023. 30953569 10.1093/gigascience/giz023 PMC6451197

[47] Boyle JE, Smith DC. Biochemical interactions between the symbionts of *Convoluta roscoffensis*. Proc R Soc Lond B Biol Sci. 1975;189:121–35.

[48] Gschwentner R, Ladurner P, Salvenmoser W, Rieger R, Tyler S. Fine structure and evolutionary significance of sagittocysts of *Convolutriloba longifissura* (Acoela, Platyhelminthes). Invertebr Biol. 1999;118:332. 10.2307/3227005.

[49] Luther A. Studien über Acöle Turbellarien aus dem finnischen Meerbusen. Acta Soc Fauna Flora Fenn. 1912;36:1–60.

[50] The Stylet. https://acoela.myspecies.info/en/gallery. Accessed 13 Jun 2025.

[51] Egger B, Steinke D, Tarui H, De Mulder K, Arendt D, Borgonie G, et al. To be or not to be a flatworm: the acoel controversy. PLoS One. 2009;4:e5502. 10.1371/journal.pone.0005502. 19430533 10.1371/journal.pone.0005502 PMC2676513

[52] Tekle YI, Raikova OI, Jondelius U. A new viviparous acoel *Childia vivipara* sp. nov. with observations on the developing embryos, sperm ultrastructure, body wall and stylet musculatures. Acta Zool. 2006;87:121–30. 10.1111/j.1463-6395.2006.00225.x.

[53] Nilsson KS, Wallberg A, Jondelius U. New species of acoela from the mediterranean, the red sea, and the south pacific. Zootaxa. 2011;2867:1–31.

[54] Ahyong S, Boyko CB, Bernot J, Brandão SN, Daly M, De Grave S, et al. World register of marine species (WoRMS). 2025.

[55] Barneah O, Brickner I, Hooge M, Weis VM, Benayahu Y. First evidence of maternal transmission of algal endosymbionts at an oocyte stage in a triploblastic host, with observations on reproduction in *Waminoa brickneri* (Acoelomorpha). Invertebr Biol. 2007;126:113–9. 10.1111/j.1744-7410.2007.00082.x.

[56] Winsor L. Marine turbellaria (Acoela) from north Queensland. Mem Queensl Mus. 1990;28:785–800.

[57] Corrêa DD. Two new marine turbellaria from florida. Bull Mar Sci. 1960;10:208–16.

[58] Børve A, Hejnol A. Development and juvenile anatomy of the nemertodermatid *Meara stichopi* (Bock) Westblad 1949 (Acoelomorpha). Front Zool. 2014;11:50. 10.1186/1742-9994-11-50. 25024737 10.1186/1742-9994-11-50 PMC4094782

[59] Martinek I, Raikova O, Jondelius U. The muscular system of *Meara stichopi* and *Nemertoderma westbladi* (Nemertodermatida). Zoomorphology. 2012;132:239–52. 10.1007/s00435-013-0191-6.

[60] Taylor DL. On the symbiosis between *Amphidinium klebsi*i [Dinophyceae] and *Amphiscolops langerhansi* [Turbellaria: Acoela]. J Mar Biol Assoc U K. 1971;51:301–13. 10.1017/S0025315400031799.

[61] Hooge M, Tyler S. New tools for resolving phylogenies: a systematic revision of the Convolutidae (Acoelomorpha, Acoela). J Zool Syst Evol Res. 2005;43:100–13.

[62] Achatz JG, Hooge MD. Convolutidae (Acoela) from Tanzania. Zootaxa. 2006;1362:1–21. 10.5281/ZENODO.174702.

[63] Asai M, Miyazawa H, Yanase R, Inaba K, Nakano H. A New Species of Acoela Possessing a Middorsal Appendage with a Possible Sensory Function. Zoolog Sci. 2022;39. 10.2108/zs210058. 10.2108/zs210058 35107002

[64] Marcus EDB-R. On turbellaria and polygordius from the Brazilian coast. Bol Fac Filos Cienc E Let Universidade Sao Paulo Zool. 1955;20:19. 10.11606/issn.2526-3382.bffclzoologia.1955.120211.

[65] Hanson ED. *Convoluta sutcliffei*, a new species of Acoelous Turbellaria. Trans Am Microsc Soc. 1961;80:423. 10.2307/3223601.

[66] Achatz JG, Gschwentner R, Rieger R. Symsagittifera smaragdina Achatz, Gschwentner & Rieger, 2005, sp. nov. Zootaxa. 2005;1085:33–45. 10.5281/zenodo.170467.

[67] Dittmann IL, Zauchner T, Nevard LM, Telford MJ, Egger B. SALMFamide2 and serotonin immunoreactivity in the nervous system of some acoels (Xenacoelomorpha). J Morphol. 2018;279:589–97. 10.1002/jmor.20794. 29388261 10.1002/jmor.20794 PMC5947262

[68] Achatz JG, Hooge MD, Tyler S. Convolutidae (Acoela) from Belize. Zootaxa. 2007;1479. 10.11646/zootaxa.1479.1.4.

[69] Hyman LH. Acoel and polyclad turbellaria from bermuda and the sargassum. Bull Bingham Oceanogr Collect. 1939;7:1–26, 9.

[70] von Graff L. Acoela, Rhabdocoela und Alioeocoela des Ostens der Vereinigten Staaten von Amerika. Z Wiss Zool. 1911;99:321–428.

[71] Brauner K. *Monochoerus chuni, Monochoerus boehmigi* und *Convoluta dubia*. Drei neue Turbellaria Acoela aus den Fangergebnissen der deutschen Tiefsee-Expedition des Jahres 1898. Zool Anz. 1920;52:31–7.

[72] Hooge MD. Two new families, three new genera, and four new species of acoel flatworms (Acoela, Platyelminthes) from Queensland, Australia. Cah Biol Mar. 2003;44:275–98.

[73] Hooge MD, Smith JPS. New acoels (Acoela, Acoelomorpha) from North Carolina. Zootaxa. 2004;442:1. 10.11646/zootaxa.442.1.1.

[74] Hooge MD, Rocha CEF. Acoela (Acoelomorpha) from the northern beaches of the state of São Paulo, Brazil, and a systematic revision of the family Otocelididae. Zootaxa. 2006. 10.5281/ZENODO.174287.

[75] Beltagi SM. *Anaperus trifurcatus* nov. sp.(archoophora: Anaperidae): a new species of acoelan turbellaria from the red sea. Mar Scienes-Ceased Lssuerg. 1983;17:1–2.

[76] von Graff L. Monographie der turbellarien 1. Rhabdocoelida. Wilhelm Engelmann Leipz. 1882.

[77] Dörjes J. Die Acoela (Turbellaria) der Deutschen Nordseeküste und ein neues System der Ordnung. J Zool Syst Evol Res. 1986;6:56–452. 10.1111/j.1439-0469.1968.tb00431.x.

[78] Marcus E. Turbellaria Brasileiros. Bol Fac Ciênc Letr Univ São Paulo Zool. 1950;15:5–192.

[79] Steinböck O. Marine turbellaria. Levin and Munksgaard; 1938.

[80] Hooge MD, Tyler S. Acoels (platyhelminthes, acoela) from the Atlantic coast of North America. Meiofauna Mar. 2003;12:7–36.

[81] Schultze M. Beiträge zur naturgeschichte der turbellarien. Greifswald. 1851. 10.5962/bhl.title.9163.

[82] Tyler S, Schilling S, Hooge M, Bush LF. Turbellarian Taxonomic Database. 2006. https://turbellaria.umaine.edu/.

[83] Mamkaev YuV. Étude Morphologique d’*Actinoposthia beklemischevi *n. sp. (Turbellaria Acoela). Cah Biol Mar. 1965;6:23–50. 10.21411/CBM.A.1A85EFDE.

[84] Hooge MD, Tyler S. Body-wall musculature of *Praeconvoluta tornuva* n. sp. (Acoela, Platyhelminthes) and the use of muscle patterns in taxonomy. Invertebr Biol. 1999;118:8. 10.2307/3226907.

[85] Kozloff EN. New species of acoel turbellarians from the Pacific coast. Biol Bull. 1965;129:151–66. 10.2307/1539774.

[86] Hooge MD, Tyler S. Acoela (acoelomorpha) from bocas del toro, panama. Zootaxa. 2008.

[87] Tekle YI. A new *Haploposthia* species (Acoela) from the Swedish west coast. Sarsia. 2004;89:85–90. 10.1080/00364820410003441.

[88] Westblad E. Studien ueber skandinavische turbellaria acoela. III. Ark For Zool. 1945;36A:1–56.

[89] Hooge MD, Tyler S. Interstitial acoels (platyhelminthes: Acoela) from bermuda. Proc Biol Soc Wash. 2001;114:414–26.

[90] der An Lan H. Ergebnisse einer von E. Reisinger und O. Steinböck mit hilfe des raskorested fonds durchgeführten reise in grönland 1926. 7. Acoela I. Vidensk Medd Dansk Naturh Foren. 1936;99:289–329.

[91] Crezée M. Monograph of the solenofilomorphidae (Turbellaria: Acoela). Internationale Revue der gesamten Hydrobiologie und Hydrographie. 1975;60:769–845. 10.1002/iroh.19750600604.

[92] Papi F. Sopra un nuovo Turbellario arcooforo di particulare significato filetico e sulla posizione della fam. Hofsteniidae Nel Sist Dei Turbellari Pubbl Staz Zool Napoli. 1957;30:132–48.

[93] Westblad E. Studien ueber skandinavische turbellaria acoela. IV. Ark For Zool. 1946;38A:1–56.

[94] Westblad E. Studien ueber skandinavische turbellaria acoela. I. Ark För Zool. 1940;33A:1–48.

[95] Faubel A. Die acoela (turbellaria) eines sandstrandes der nordseeinsel sylt. Mainz: Akademie d. Wiss. ud Literatur; 1974.

[96] Westblad E. Studien ueber skandinavische turbellaria acoela. II. Ark För Zool. 1942;33A:1–48.

[97] Kånneby T, Bernvi DC, Jondelius U. Distribution, delimitation and description of species of *Archaphanostoma* (Acoela). Zool Scr. 2015;44:218–31. 10.1111/zsc.12092.

[98] Jensen OS. Turbellaria ad litora Norvegiae occidentalia. Turbellarier ved Norges vestkyst. Bergen, J. W. Eides bogtrykkeri; 1878.

[99] Rouse GW, Wilson NG, Carvajal JI, Vrijenhoek RC. New deep-sea species of *Xenoturbella* and the position of Xenacoelomorpha. Nature. 2016;530:94–7. 10.1038/nature16545. 26842060 10.1038/nature16545

[100] Israelsson O. New light on the enigmatic *Xenoturbella* (phylum uncertain): ontogeny and phylogeny. Proc R Soc Lond B Biol Sci. 1999;266:835–41. 10.1098/rspb.1999.0713.

[101] Meyer-Wachsmuth I, Curini Galletti M, Jondelius U. Hyper-cryptic marine meiofauna: species complexes in Nemertodermatida. PLoS ONE. 2014;9:e107688. 10.1371/journal.pone.0107688. 25225981 10.1371/journal.pone.0107688 PMC4166464

[102] Abalde S, Jondelius U. A phylogenomic backbone for Acoelomorpha inferred from transcriptomic data. Syst Biol. 2024;74(1):70–85. 10.1093/sysbio/syae057. 10.1093/sysbio/syae057 PMC11809588 39451056

[103] Jondelius U, Wallberg A, Hooge M, Raikova OI. How the worm got its pharynx: phylogeny, classification and Bayesian assessment of character evolution in Acoela. Syst Biol. 2011;60(6):845–71. 10.1093/sysbio/syr073. 21828080 10.1093/sysbio/syr073

[104] Dunn CW, Giribet G, Edgecombe GD, Hejnol A. Animal phylogeny and its evolutionary implications. Annu Rev Ecol Evol Syst. 2014;45(1):371–95. 10.1146/annurev-ecolsys-120213-091627.

[105] Laumer CE, Gruber-Vodicka H, Hadfield MG, Pearse VB, Riesgo A, Marioni JC, et al. Support for a clade of Placozoa and Cnidaria in genes with minimal compositional bias. eLife. 2018;7:e36278. 10.7554/eLife.36278. 30373720 10.7554/eLife.36278 PMC6277202

[106] Schultz DT, Haddock SHD, Bredeson JV, Green RE, Simakov O, Rokhsar DS. Ancient gene linkages support ctenophores as sister to other animals. Nature. 2023;618(7963):110–7. 10.1038/s41586-023-05936-6. 37198475 10.1038/s41586-023-05936-6 PMC10232365

[107] Najle SR, Grau-Bové X, Elek A, Navarrete C, Cianferoni D, Chiva C, et al. Stepwise emergence of the neuronal gene expression program in early animal evolution. Cell. 2023;186:4676-4693.e29. 10.1016/j.cell.2023.08.027. 37729907 10.1016/j.cell.2023.08.027 PMC10580291

[108] Cannon JT, Vellutini BC, Smith J, Ronquist F, Jondelius U, Hejnol A. Xenacoelomorpha is the sister group to Nephrozoa. Nature. 2016;530:89–93. 10.1038/nature16520. 26842059 10.1038/nature16520

[109] Philippe H, Brinkmann H, Copley RR, Moroz LL, Nakano H, Poustka AJ, et al. Acoelomorph flatworms are deuterostomes related to Xenoturbella. Nature. 2011;470:255–8. 10.1038/nature09676. 21307940 10.1038/nature09676 PMC4025995

[110] Kamm K, Schierwater B, DeSalle R. Innate immunity in the simplest animals – placozoans. BMC Genomics. 2019;20(1):5. 10.1186/s12864-018-5377-3. 30611207 10.1186/s12864-018-5377-3 PMC6321704

[111] Orús-Alcalde A, Lu T-M, Børve A, Hejnol A. The evolution of the metazoan toll receptor family and its expression during protostome development. BMC Ecol Evol. 2021;21(1):208. 10.1186/s12862-021-01927-1. 34809567 10.1186/s12862-021-01927-1 PMC8609888

[112] Song X, Jin P, Qin S, Chen L, Ma F. The evolution and origin of animal toll-like receptor signaling pathway revealed by network-level molecular evolutionary analyses. PLoS One. 2012;7:e51657. 10.1371/journal.pone.0051657. 23236523 10.1371/journal.pone.0051657 PMC3517549

[113] Zelensky AN, Gready JE. The c-type lectin-like domain superfamily. FEBS J. 2005;272(24):6179–217. 10.1111/j.1742-4658.2005.05031.x. 16336259 10.1111/j.1742-4658.2005.05031.x

[114] Neubauer EF, Poole AZ, Weis VM, Davy SK. The scavenger receptor repertoire in six cnidarian species and its putative role in cnidarian-dinoflagellate symbiosis. PeerJ. 2016;4:e2692. 10.7717/peerj.2692. 27896028 10.7717/peerj.2692 PMC5119243

[115] Pancer Z, Münkner J, Müller I, Müller WEG. A novel member of an ancient superfamily: sponge (*Geodia cydonium*, Porifera) putative protein that features scavenger receptor cysteine-rich repeats. Gene. 1997;193(2):211–8. 10.1016/S0378-1119(97)00135-2. 9256079 10.1016/s0378-1119(97)00135-2

[116] Canton J, Neculai D, Grinstein S. Scavenger receptors in homeostasis and immunity. Nat Rev Immunol. 2013;13(9):621–34. 10.1038/nri3515. 23928573 10.1038/nri3515

[117] Melo Clavijo J, Frankenbach S, Fidalgo C, Serôdio J, Donath A, Preisfeld A, et al. Identification of scavenger receptors and thrombospondin‐type‐1 repeat proteins potentially relevant for plastid recognition in *Sacoglossa*. Ecol Evol. 2020;10(21):12348–63. 10.1002/ece3.6865. 33209293 10.1002/ece3.6865 PMC7663992

[118] Zhu X, Mu K, Wan Y, Zhang L. Evolutionary history of the NLR gene families across lophotrochozoans. Gene. 2022;843:146807. 10.1016/j.gene.2022.146807. 35964873 10.1016/j.gene.2022.146807

[119] Koutsouveli V, Torres-Oliva M, Bayer T, Fuß J, Grossschmidt N, Marulanda-Gomez AM, et al. The chromosome-level genome of the ctenophore* Mnemiopsis leidyi* A. Agassiz, 1865 reveals a unique immune gene repertoire. Genome Biol Evol. 2025;17:evaf006. 10.1093/gbe/evaf006. 39834228 10.1093/gbe/evaf006 PMC11797021

[120] Traylor-Knowles N, Vandepas LE, Browne WE. Still enigmatic: innate immunity in the ctenophore *Mnemiopsis leidyi*. Integr Comp Biol. 2019;59(4):811–8. 10.1093/icb/icz116. 31251332 10.1093/icb/icz116

[121] Jacobovitz MR, Rupp S, Voss PA, Maegele I, Gornik SG, Guse A. Dinoflagellate symbionts escape vomocytosis by host cell immune suppression. Nat Microbiol. 2021;6(6):769–82. 10.1038/s41564-021-00897-w. 33927382 10.1038/s41564-021-00897-w PMC7611106

[122] Lazzaro BP, Clark AG. Rapid evolution of innate immune response genes. In: Singh RS, Xu J, Kulathinal RJ, editors. Rapidly evolving genes and genetic systems. Oxford: Oxford University Press; 2012.

[123] Rast JP, Messier-Solek C. Marine invertebrate genome sequences and our evolving understanding of animal immunity. Biol Bull. 2008;214(3):274–83. 10.2307/25470669. 18574104 10.2307/25470669

[124] Pradeu T, Thomma BPHJ, Girardin SE, Lemaitre B. The conceptual foundations of innate immunity: taking stock 30 years later. Immunity. 2024;57(4):613–31. 10.1016/j.immuni.2024.03.007. 38599162 10.1016/j.immuni.2024.03.007

[125] Li R, Qu J, Li H, Zhang Q. Genome-wide identification and analysis of scavenger receptors and their expression profiling in response to *Edwardsiella tarda* infection in Japanese flounder (*Paralichthys olivaceus*). Dev Comp Immunol. 2022;132:104397. 10.1016/j.dci.2022.104397. 35307477 10.1016/j.dci.2022.104397

[126] PrabhuDas MR, Baldwin CL, Bollyky PL, Bowdish DME, Drickamer K, Febbraio M, et al. A consensus definitive classification of scavenger receptors and their roles in health and disease. J Immunol. 2017;198(10):3775–89. 10.4049/jimmunol.1700373. 28483986 10.4049/jimmunol.1700373 PMC5671342

[127] Sattler S, Ghadially H, Hofer E. Evolution of the c-type lectin-like receptor genes of the DECTIN-1 cluster in the NK gene complex. Sci World J. 2012;2012:1–11. 10.1100/2012/931386. 10.1100/2012/931386 PMC3322459 22550468

[128] Gorbushin AM. Derivatives of the lectin complement pathway in Lophotrochozoa. Dev Comp Immunol. 2019;94:35–58. 10.1016/j.dci.2019.01.010. 30682446 10.1016/j.dci.2019.01.010

[129] Davidson CR, Best NM, Francis JW, Cooper EL, Wood TC. Toll-like receptor genes (TLRs) from *Capitella capitata* and *Helobdella robusta* (Annelida). Dev Comp Immunol. 2008;32(6):608–12. 10.1016/j.dci.2007.11.004. 18164761 10.1016/j.dci.2007.11.004

[130] Dunkelberger JR, Song W-C. Complement and its role in innate and adaptive immune responses. Cell Res. 2010;20(1):34–50. 10.1038/cr.2009.139. 20010915 10.1038/cr.2009.139

[131] Frank MM. Complement disorders and hereditary angioedema. J Allergy Clin Immunol. 2010;125(2):S262-71. 10.1016/j.jaci.2009.10.063. 20176263 10.1016/j.jaci.2009.10.063

[132] Fujita T. Evolution of the lectin-complement pathway and its role in innate immunity. Nat Rev Immunol. 2002;2(5):346–53. 10.1038/nri800. 12033740 10.1038/nri800

[133] Nonaka M, Kimura A. Genomic view of the evolution of the complement system. Immunogenetics. 2006;58(9):701–13. 10.1007/s00251-006-0142-1. 16896831 10.1007/s00251-006-0142-1 PMC2480602

[134] Zhao B-R, Wang X-X, Liu P-P, Wang X-W. Complement-related proteins in crustacean immunity. Dev Comp Immunol. 2022;139:104577. 10.1016/j.dci.2022.104577. 36265592 10.1016/j.dci.2022.104577

[135] Miller DJ, Hemmrich G, Ball EE, Hayward DC, Khalturin K, Funayama N, et al. The innate immune repertoire in Cnidaria - ancestral complexity and stochastic gene loss. Genome Biol. 2007;8(4):R59. 10.1186/gb-2007-8-4-r59. 17437634 10.1186/gb-2007-8-4-r59 PMC1896004

[136] Nonaka M. The complement C3 protein family in invertebrates. Invertebr Surviv J. 2011;8(1):21–32.

[137] Peng M, Li Z, Cardoso JCR, Niu D, Liu X, Dong Z, et al. Domain-Dependent evolution explains functional homology of Protostome and Deuterostome complement C3-Like proteins. Front Immunol. 2022;13:840861. 10.3389/fimmu.2022.840861. 35359984 10.3389/fimmu.2022.840861 PMC8960428

[138] Sekiguchi R, Fujito NT, Nonaka M. Evolution of the thioester-containing proteins (TEPs) of the arthropoda, revealed by molecular cloning of TEP genes from a spider, *Hasarius adansoni*. Dev Comp Immunol. 2012;36(2):483–9. 10.1016/j.dci.2011.05.003. 21663759 10.1016/j.dci.2011.05.003

[139] Gorbushin AM. Immune repertoire in the transcriptome of *Littorina littorea* reveals new trends in lophotrochozoan proto-complement evolution. Dev Comp Immunol. 2018;84:250–63. 10.1016/j.dci.2018.02.018. 29501422 10.1016/j.dci.2018.02.018

[140] Peng M, Li Z, Niu D, Liu X, Dong Z, Li J. Complement factor B/C2 in molluscs regulates agglutination and illuminates evolution of the Bf/C2 family. FASEB J. 2019;33(12):13323–33. 10.1096/fj.201901142RR. 31550175 10.1096/fj.201901142RR

[141] Poole AZ, Kitchen SA, Weis VM. The Role of Complement in Cnidarian-Dinoflagellate Symbiosis and Immune Challenge in the Sea Anemone *Aiptasia pallida*. Front Microbiol. 2016;7. 10.3389/fmicb.2016.00519. 10.3389/fmicb.2016.00519 PMC4840205 27148208

[142] Smith LC, Azumi K, Nonaka M. Complement systems in invertebrates. The ancient alternative and lectin pathways. Immunopharmacology. 1999;42(1–3):107–20. 10.1016/s0162-3109(99)00009-0. 10408372 10.1016/s0162-3109(99)00009-0

[143] Ahearn JM, Fearon DT. Structure and Function of the Complement Receptors, CR1 (CD35) and CR2 (CD21). In: Advances in Immunology. Elsevier; 1989. p. 183–219. 10.1016/S0065-2776(08)60654-9. 10.1016/s0065-2776(08)60654-9 2551147

[144] Aderem A, Ulevitch RJ. Toll-like receptors in the induction of the innate immune response. Nature. 2000;406(6797):782–7. 10.1038/35021228. 10963608 10.1038/35021228

[145] Friedman R, Hughes AL. Molecular evolution of the NF-κB signaling system. Immunogenetics. 2002;53(10–11):964–74. 10.1007/s00251-001-0399-3. 11862396 10.1007/s00251-001-0399-3

[146] Gosu V, Basith S, Durai P, Choi S. Molecular evolution and structural features of IRAK family members. PLoS One. 2012;7(11):e49771. 10.1371/journal.pone.0049771. 23166766 10.1371/journal.pone.0049771 PMC3498205

[147] Kawai T, Akira S. The role of pattern-recognition receptors in innate immunity: update on Toll-like receptors. Nat Immunol. 2010;11(5):373–84. 10.1038/ni.1863. 20404851 10.1038/ni.1863

[148] Ren Y, Xue J, Yang H, Pan B, Bu W. Comparative and evolutionary analysis of an adapter molecule MyD88 in invertebrate metazoans. Dev Comp Immunol. 2017;76:18–24. 10.1016/j.dci.2017.05.007. 28502652 10.1016/j.dci.2017.05.007

[149] Tassia MG, Whelan NV, Halanych KM. Toll-like receptor pathway evolution in deuterostomes. Proc Natl Acad Sci U S A. 2017;114(27):7055–60. 10.1073/pnas.1617722114. 28630328 10.1073/pnas.1617722114 PMC5502590

[150] Deguine J, Barton GM. MyD88: a central player in innate immune signaling. F1000Prime Rep. 2014;6:97. 10.12703/P6-97. 25580251 10.12703/P6-97 PMC4229726

[151] Ghosh S, May MJ, Kopp EB. NF-κB and Rel proteins: evolutionarily conserved mediators of immune responses. Annu Rev Immunol. 1998;16(1):225–60. 10.1146/annurev.immunol.16.1.225. 9597130 10.1146/annurev.immunol.16.1.225

[152] Leger MM, Ros-Rocher N, Najle SR, Ruiz-Trillo I. Rel/NF-κB transcription factors emerged at the onset of Opisthokonts. Genome Biol Evol. 2022;14(1):evab289. 10.1093/gbe/evab289. 34999783 10.1093/gbe/evab289 PMC8763368

[153] Corrêa DD. Two new marine turbellaria from Florida. Bull Mar Sci. 1960;10(2):208–16.

[154] Ben-Haim Y, Thompson FL, Thompson CC, Cnockaert MC, Hoste B, Swings J, et al. *Vibrio coralliilyticus* sp. nov., a temperature-dependent pathogen of the coral *Pocillopora damicornis*. Int J Syst Evol Microbiol. 2003;53(1):309–15. 10.1099/ijs.0.02402-0. 12656189 10.1099/ijs.0.02402-0

[155] Kimes NE, Grim CJ, Johnson WR, Hasan NA, Tall BD, Kothary MH, et al. Temperature regulation of virulence factors in the pathogen *Vibrio coralliilyticus*. ISME J. 2012;6(4):835–46. 10.1038/ismej.2011.154. 22158392 10.1038/ismej.2011.154 PMC3309362

[156] Austin B, Austin D, Sutherland R, Thompson F, Swings J. Pathogenicity of vibrios to rainbow trout (*Oncorhynchus mykiss*, Walbaum) and *Artemia* nauplii. Environ Microbiol. 2005;7(9):1488–95. 10.1111/j.1462-2920.2005.00847.x. 16104871 10.1111/j.1462-2920.2005.00847.x

[157] Balbi T, Auguste M, Cortese K, Montagna M, Borello A, Pruzzo C, et al. Responses of *Mytilus galloprovincialis* to challenge with the emerging marine pathogen *Vibrio coralliilyticus*. Fish Shellfish Immunol. 2019;84:352–60. 10.1016/j.fsi.2018.10.011. 30300739 10.1016/j.fsi.2018.10.011

[158] Li R, Dang H, Huang Y, Quan Z, Jiang H, Zhang W, et al. *Vibrio coralliilyticus* as an agent of red spotting disease in the sea urchin *Strongylocentrotus intermedius*. Aquac Rep. 2020;16:100244. 10.1016/j.aqrep.2019.100244.

[159] Richards GP, Watson MA, Needleman DS, Church KM, Häse CC, Griffiths MW, editor. Mortalities of eastern and Pacific oyster larvae caused by the pathogens *Vibrio coralliilyticus* and *Vibrio tubiashii*. Appl Environ Microbiol. 2015;81(1):292–7. 10.1128/AEM.02930-14. 25344234 10.1128/AEM.02930-14 PMC4272748

[160] Ushijima B, Richards GP, Watson MA, Schubiger CB, Häse CC. Factors affecting infection of corals and larval oysters by *Vibrio coralliilyticus*. PLoS One. 2018;13(6):e0199475. 10.1371/journal.pone.0199475. 29920567 10.1371/journal.pone.0199475 PMC6007914

[161] Ushijima B, Meyer JL, Thompson S, Pitts K, Marusich MF, Tittl J, et al. Disease diagnostics and potential coinfections by *Vibrio coralliilyticus* during an ongoing coral disease outbreak in Florida. Front Microbiol. 2020;26(11):569354. 10.3389/fmicb.2020.569354. 10.3389/fmicb.2020.569354 PMC7649382 33193161

[162] Xu D, Zhao Z, Zhou Z, Lin Y, Zhang X, Zhang Y, et al. Mechanistic molecular responses of the giant clam *Tridacna crocea* to *Vibrio coralliilyticus* challenge. PLoS One. 2020;15(4):e0231399. 10.1371/journal.pone.0231399. 32276269 10.1371/journal.pone.0231399 PMC7148125

[163] Zaragoza WJ, Krediet CJ, Meyer JL, Canas G, Ritchie KB, Teplitski M. Outcomes of infections of sea anemone *Aiptasia pallida* with *Vibrio* spp. pathogenic to corals. Microb Ecol. 2014;68(2):388–96. 10.1007/s00248-014-0397-2. 24619233 10.1007/s00248-014-0397-2

[164] Alves N Jr, Neto OSM, Silva BSO, De Moura RL, Francini-Filho RB, Barreira E, et al. Diversity and pathogenic potential of vibrios isolated from Abrolhos Bank corals. Environ Microbiol Rep. 2010;2(1):90–5. 10.1111/j.1758-2229.2009.00101.x. 23766002 10.1111/j.1758-2229.2009.00101.x

[165] De O, Santos E, Alves N, Dias GM, Mazotto AM, Vermelho A, et al. Genomic and proteomic analyses of the coral pathogen *Vibrio coralliilyticus* reveal a diverse virulence repertoire. ISME J. 2011;5(9):1471–83. 10.1038/ismej.2011.19. 21451583 10.1038/ismej.2011.19 PMC3160677

[166] Ben-Haim Y, Zicherman-Keren M, Rosenberg E. Temperature-regulated bleaching and lysis of the coral* Pocillopora damicornis* by the novel pathogen* Vibrio coralliilyticus*. Appl Environ Microbiol. 2003;69(7):4236–42. 10.1128/AEM.69.7.4236-4242.2003. 12839805 10.1128/AEM.69.7.4236-4242.2003 PMC165124

[167] Vidal-Dupiol J, Ladrière O, Destoumieux-Garzón D, Sautière PE, Meistertzheim AL, Tambutté E, et al. Innate immune responses of a scleractinian coral to vibriosis. J Biol Chem. 2011;286(25):22688–98. 10.1074/jbc.M110.216358. 21536670 10.1074/jbc.M110.216358 PMC3121412

[168] Vidal-Dupiol J, Ladrière O, Meistertzheim AL, Fouré L, Adjeroud M, Mitta G. Physiological responses of the scleractinian coral *Pocillopora damicornis* to bacterial stress from Vibrio coralliilyticus. J Exp Biol. 2011;214(9):1533–45. 10.1242/jeb.053165. 21490261 10.1242/jeb.053165

[169] Ushijima B, Videau P, Burger AH, Shore-Maggio A, Runyon CM, Sudek M, et al. *Vibrio coralliilyticus* strain OCN008 is an etiological agent of acute Montipora white syndrome. Appl Environ Microbiol. 2014;80(7):2102–9. 10.1128/AEM.03463-13. 24463971 10.1128/AEM.03463-13 PMC3993142

[170] Zhou Z, Zhao S, Tang J, Liu Z, Wu Y, Wang Y, et al. Altered immune landscape and disrupted coral-*Symbiodinium* symbiosis in the Scleractinian coral *Pocillopora damicornis* by *Vibrio coralliilyticus* challenge. Front Physiol. 2019;2(10):366. 10.3389/fphys.2019.00366. 10.3389/fphys.2019.00366 PMC6454040 31001143

[171] Gupta RS, Patel S, Saini N, Chen S. Robust demarcation of 17 distinct *Bacillus* species clades, proposed as novel *Bacillaceae* genera, by phylogenomics and comparative genomic analyses: description of *Robertmurraya kyonggiensis* sp. nov. and proposal for an emended genus *Bacillus* limiting it only to the members of the Subtilis and Cereus clades of species. Int J Syst Evol Microbiol. 2020;70(11):5753–98. 10.1099/ijsem.0.004475. 33112222 10.1099/ijsem.0.004475

[172] Jandang S, Bulan DE, Chavanich S, Viyakarn V, Aiemsomboon K, Somboonna N. First report of potential coral disease in the coral hatchery of Thailand. Diversity. 2021;14(1):18. 10.3390/d14010018.

[173] Mass S, Cohen H, Podicheti R, Rusch DB, Gerlic M, Ushijima B, et al. The coral pathogen *Vibrio coralliilyticus* uses a T6SS to secrete a group of novel anti-eukaryotic effectors that contribute to virulence. PLoS Biol. 2024;22(9):e3002734. 10.1371/journal.pbio.3002734. 39226241 10.1371/journal.pbio.3002734 PMC11371242

[174] Corbari L, Durand L, Cambon-Bonavita MA, Gaill F, Compère P. New digestive symbiosis in the hydrothermal vent amphipoda *Ventiella sulfuris*. CR Biol. 2012;335(2):142–54. 10.1016/j.crvi.2011.12.005. 10.1016/j.crvi.2011.12.005 22325568

[175] Huotari J, Helenius A. Endosome maturation: endosome maturation. EMBO J. 2011;30(17):3481–500. 10.1038/emboj.2011.286. 21878991 10.1038/emboj.2011.286 PMC3181477

[176] Beveridge TJ. Structures of gram-negative cell walls and their derived membrane vesicles. J Bacteriol. 1999;181(16):4725–33. 10.1128/JB.181.16.4725-4733.1999. 10438737 10.1128/jb.181.16.4725-4733.1999 PMC93954

[177] Rozenblat YBH, Rosenberg E. Temperature-regulated bleaching and tissue lysis of* Pocillopora damicornis* by the novel pathogen *Vibrio coralliilyticus*. In: Rosenberg E, Loya Y, editors. Coral Health and Disease. Berlin, Heidelberg: Springer Berlin Heidelberg; 2004. p.301-24. 10.1007/978-3-662-06414-6_17.

[178] Ben-Haim Y, Rosenberg E. A novel *Vibrio* sp. pathogen of the coral *Pocillopora damicornis*. Mar Biol. 2002;141(1):47–55.

[179] Wang Y, Osakue D, Yang E, Zhou Y, Gong H, Xia X, et al. Endoplasmic reticulum stress response of trabecular meshwork stem cells and trabecular meshwork cells and protective effects of activated PERK pathway. Invest Ophthalmol Vis Sci. 2019;60(1):265. 10.1167/iovs.18-25477. 30654386 10.1167/iovs.18-25477 PMC6340162

[180] Cheville NF. Cell pathology. 2nd ed. Ames, Iowa, USA: The Iowa State University Press; 1983.

[181] Bonanno E, Ruzittu M, Carlà EC, Montinari MR, Pagliara P, Dini L. Cell shape and organelle modification in apoptotic U937 cells. Eur J Histochem EJH. 2000;44(3):237–46. 11095095

[182] Burattini S, Falcieri E. Analysis of cell death by electron microscopy. In: McCall K, Klein C, editors. Necrosis. Totowa, NJ: Humana Press; 2013. p. 77–89. 10.1007/978-1-62703-383-1_7. 10.1007/978-1-62703-383-1_7 23733571

[183] Zélé F, Santos-Matos G, Figueiredo ART, Eira C, Pinto C, Laurentino TG, et al. Spider mites escape bacterial infection by avoiding contaminated food. Oecologia. 2019;189(1):111–22. 10.1007/s00442-018-4316-y. 30511092 10.1007/s00442-018-4316-y

[184] Parker BJ, Barribeau SM, Laughton AM, Griffin LH, Gerardo NM. Life‐history strategy determines constraints on immune function. J Anim Ecol. 2017;86(3):473–83. 10.1111/1365-2656.12657. 28211052 10.1111/1365-2656.12657

[185] Weil E, Cróquer A, Urreiztieta I. Yellow band disease compromises the reproductive output of the Caribbean reef-building coral *Montastraea faveolata* (Anthozoa, Scleractinia). Dis Aquat Organ. 2009;87(1–2):45–55. 20095240 10.3354/dao02103

[186] Zélé F, Denoyelle J, Duron O, Rivero A. Can *Wolbachia* modulate the fecundity costs of *Plasmodium* in mosquitoes? Parasitology. 2018;145(6):775–82. 10.1017/S0031182017001330. 28786370 10.1017/S0031182017001330

[187] Bonneaud C, Mazuc J, Chastel O, Westerdahl H, Sorci G. Terminal investment induced by immune challenge and fitness traits associated with major histocompatibility complex in the house sparrow. Evolution. 2004;58(12):2823–30. 15696759 10.1111/j.0014-3820.2004.tb01633.x

[188] Clutton-Brock TH. Reproductive effort and terminal investment in iteroparous animals. Am Nat. 1984;123(2):212–29.

[189] Heins DC. Fecundity compensation in the three-spined stickleback *Gasterosteus aculeatus* infected by the diphyllobothriidean cestode *Schistocephalus solidus*: fecundity compensation in sticklebacks. Biol J Linn Soc. 2012;106(4):807–19. 10.1111/j.1095-8312.2012.01907.x.

[190] Foo YZ, Lagisz M, O’Dea RE, Nakagawa S. The influence of immune challenges on the mean and variance in reproductive investment: a meta-analysis of the terminal investment hypothesis. BMC Biol. 2023;21(1):107. 10.1186/s12915-023-01603-4. 37173684 10.1186/s12915-023-01603-4 PMC10176797

[191] Williams GC. Natural selection, the costs of reproduction, and a refinement of lack’s principle. Am Nat. 1966;100(916):687–90. 10.1086/282461.

[192] Tak U, Dokland T, Niederweis M. Pore-forming Esx proteins mediate toxin secretion by *Mycobacterium tuberculosis*. Nat Commun. 2021;12(1):394. 10.1038/s41467-020-20533-1. 33452244 10.1038/s41467-020-20533-1 PMC7810871

[193] Rosado CJ, Buckle AM, Law RHP, Butcher RE, Kan WT, Bird CH, et al. A common fold mediates vertebrate defense and bacterial attack. Science. 2007;317(5844):1548–51. 10.1126/science.1144706. 17717151 10.1126/science.1144706

[194] Jiang RHY, Tripathy S, Govers F, Tyler BM. RXLR effector reservoir in two *Phytophthora* species is dominated by a single rapidly evolving superfamily with more than 700 members. Proc Natl Acad Sci USA. 2008;105(12):4874–9. 10.1073/pnas.0709303105. 18344324 10.1073/pnas.0709303105 PMC2290801

[195] Heutinck KM, Ten Berge IJM, Hack CE, Hamann J, Rowshani AT. Serine proteases of the human immune system in health and disease. Mol Immunol. 2010;47(11–12):1943–55. 10.1016/j.molimm.2010.04.020. 20537709 10.1016/j.molimm.2010.04.020

[196] Riewluang S, Wakeman KC. Biodiversity of symbiotic microalgae associated with meiofaunal marine acoels in Southern Japan. PeerJ. 2023;5(11):e16078. 10.7717/peerj.16078. 10.7717/peerj.16078 PMC10560497 37814628

[197] Roach JM, Racioppi L, Jones CD, Masci AM, Gay N. Phylogeny of Toll-like receptor signaling: adapting the innate response. PLoS One. 2013;8(1):e54156. 10.1371/journal.pone.0054156. 23326591 10.1371/journal.pone.0054156 PMC3543326

[198] Wu B, Xin B, Jin M, Wei T, Bai Z. Comparative and phylogenetic analyses of three TIR domain-containing adaptors in metazoans: implications for evolution of TLR signaling pathways. Dev Comp Immunol. 2011;35(7):764–73. 10.1016/j.dci.2011.02.009. 21362440 10.1016/j.dci.2011.02.009

[199] Melo Clavijo J, Sickinger C, Bleidißel S, Gasparoni G, Tierling S, Preisfeld A, et al. The nudibranch *Berghia stephanieae* (Valdés, 2005) is not able to initiate a functional symbiosome-like environment to maintain *Breviolum minutum* (J.E.Parkinson & LaJeunesse 2018). Front Mar Sci. 2022;9:934307. 10.3389/fmars.2022.934307.

[200] Ganot P, Moya A, Magnone V, Allemand D, Furla P, Sabourault C, et al. Adaptations to endosymbiosis in a cnidarian-dinoflagellate association: differential gene expression and specific gene duplications. PLoS Genet. 2011;7(7):e1002187. 10.1371/journal.pgen.1002187. 21811417 10.1371/journal.pgen.1002187 PMC3141003

[201] Kvennefors ECE, Leggat W, Kerr CC, Ainsworth TD, Hoegh-Guldberg O, Barnes AC. Analysis of evolutionarily conserved innate immune components in coral links immunity and symbiosis. Dev Comp Immunol. 2010;34(11):1219–29. 10.1016/j.dci.2010.06.016. 20600272 10.1016/j.dci.2010.06.016

[202] Philippe H, Poustka AJ, Chiodin M, Hoff KJ, Dessimoz C, Tomiczek B, et al. Mitigating anticipated effects of systematic errors supports sister-group relationship between Xenacoelomorpha and Ambulacraria. Curr Biol. 2019;29(11):1818-1826.e6. 10.1016/j.cub.2019.04.009. 31104936 10.1016/j.cub.2019.04.009

[203] Hammer TJ, Sanders JG, Fierer N. Not all animals need a microbiome. FEMS Microbiol Lett. 2019;366(10):fnz117. 10.1093/femsle/fnz117. 31132110 10.1093/femsle/fnz117

[204] Vigneron A, Masson F, Vallier A, Balmand S, Rey M, Vincent-Monégat C, et al. Insects recycle endosymbionts when the benefit is over. Curr Biol. 2014;24(19):2267–73. 10.1016/j.cub.2014.07.065. 25242028 10.1016/j.cub.2014.07.065

[205] Tait K, Hutchison Z, Thompson FL, Munn CB. Quorum sensing signal production and inhibition by coral-associated vibrios. Environ Microbiol Rep. 2010;2(1):145–50. 10.1111/j.1758-2229.2009.00122.x. 23766010 10.1111/j.1758-2229.2009.00122.x

[206] Certner RH, Vollmer SV. Inhibiting bacterial quorum sensing arrests coral disease development and disease-associated microbes. Environ Microbiol. 2018;20(2):645–57. 10.1111/1462-2920.13991. 29124861 10.1111/1462-2920.13991

[207] Rubio-Portillo E, Gago JF, Martínez-García M, Vezzulli L, Rosselló-Móra R, Antón J, et al. *Vibrio* communities in scleractinian corals differ according to health status and geographic location in the Mediterranean Sea. Syst Appl Microbiol. 2018;41(2):131–8. 10.1016/j.syapm.2017.11.007. 29338888 10.1016/j.syapm.2017.11.007

[208] Brown T, Rodriguez-Lanetty M. Defending against pathogens – immunological priming and its molecular basis in a sea anemone, cnidarian. Sci Rep. 2015;5(1):17425. 10.1038/srep17425. 26628080 10.1038/srep17425 PMC4667181

[209] Cervino JM, Thompson FL, Gomez-Gil B, Lorence EA, Goreau TJ, Hayes RL, et al. The *Vibrio* core group induces yellow band disease in Caribbean and Indo-Pacific reef-building corals. J Appl Microbiol. 2008;105(5):1658–71. 10.1111/j.1365-2672.2008.03871.x. 18798767 10.1111/j.1365-2672.2008.03871.x

[210] Gibbin E, Gavish A, Krueger T, Kramarsky-Winter E, Shapiro O, Guiet R, et al. *Vibrio coralliilyticus* infection triggers a behavioural response and perturbs nutritional exchange and tissue integrity in a symbiotic coral. ISME J. 2019;13(4):989–1003. 10.1038/s41396-018-0327-2. 30542077 10.1038/s41396-018-0327-2 PMC6462045

[211] Rouzé H, Lecellier G, Saulnier D, Berteaux-Lecellier V. *Symbiodinium* clades A and D differentially predispose *Acropora cytherea* to disease and *Vibrio* spp. colonization. Ecol Evol. 2016;6(2):560–72. 10.1002/ece3.1895. 26843939 10.1002/ece3.1895 PMC4729262

[212] Valadez-Ingersoll M, Aguirre Carrión PJ, Bodnar CA, Desai NA, Gilmore TD, Davies SW. Starvation differentially affects gene expression, immunity and pathogen susceptibility across symbiotic states in a model cnidarian. Proc R Soc B Biol Sci. 2017;2024(291):20231685. 10.1098/rspb.2023.1685. 10.1098/rspb.2023.1685 PMC10898965 38412969

[213] Emery MA, Beavers KM, Van Buren EW, Batiste R, Dimos B, Pellegrino MW, et al. Trade-off between photosymbiosis and innate immunity influences cnidarian’s response to pathogenic bacteria. Proc R Soc Lond B Biol Sci. 2024;291(2032):20240428. 10.1098/rspb.2024.0428. 10.1098/rspb.2024.0428 PMC11444771 39353557

[214] Nystrand M, Dowling DK. Effects of immune challenge on expression of life-history and immune trait expression in sexually reproducing metazoans—a meta-analysis. BMC Biol. 2020;18(1):135. 10.1186/s12915-020-00856-7. 33028304 10.1186/s12915-020-00856-7 PMC7541220

[215] Gehrke AR, Srivastava M. Neoblasts and the evolution of whole-body regeneration. Curr Opin Genet Dev. 2016;40:131–7. 10.1016/j.gde.2016.07.009. 27498025 10.1016/j.gde.2016.07.009

[216] Toullec G, Rädecker N, Pogoreutz C, Banc-Prandi G, Escrig S, Genoud C, et al. Host starvation and in hospite degradation of algal symbionts shape the heat stress response of the *Cassiopea*-Symbiodiniaceae symbiosis. Microbiome. 2024;12(1):42. 10.1186/s40168-023-01738-0. 38424629 10.1186/s40168-023-01738-0 PMC10902967

[217] Altincicek B, Gross J, Vilcinskas A. Wounding-mediated gene expression and accelerated viviparous reproduction of the pea aphid *Acyrthosiphon pisum*. Insect Mol Biol. 2008;17(6):711–6. 10.1111/j.1365-2583.2008.00835.x. 18823444 10.1111/j.1365-2583.2008.00835.x

[218] Barribeau SM, Sok D, Gerardo NM. Aphid reproductive investment in response to mortality risks. BMC Evol Biol. 2010;10(1):251. 10.1186/1471-2148-10-251. 20716370 10.1186/1471-2148-10-251 PMC2940815

[219] Gerardo NM, Altincicek B, Anselme C, Atamian H, Barribeau SM, de Vos M, et al. Immunity and other defenses in pea aphids, *Acyrthosiphon pisum*. Genome Biol. 2010;11(2):R21. 10.1186/gb-2010-11-2-r21. 20178569 10.1186/gb-2010-11-2-r21 PMC2872881

[220] Gavilán B, Sprecher SG, Hartenstein V, Martinez P. The digestive system of xenacoelomorphs. Cell Tissue Res. 2019;377(3):369–82. 10.1007/s00441-019-03038-2. 31093756 10.1007/s00441-019-03038-2

[221] Hartenstein V, Martinez P. Phagocytosis in cellular defense and nutrition: a food-centered approach to the evolution of macrophages. Cell Tissue Res. 2019;377(3):527–47. 10.1007/s00441-019-03096-6. 31485720 10.1007/s00441-019-03096-6 PMC6750737

[222] Patnaik BB, Baliarsingh S, Sarkar A, Hameed ASS, Lee YS, Jo YH, et al. The role of pattern recognition receptors in crustacean innate immunity. Rev Aquacult. 2024;16(1):190–233. 10.1111/raq.12829.

[223] Pees B, Yang W, Zárate-Potes A, Schulenburg H, Dierking K. High innate immune specificity through diversified C-type lectin-like domain proteins in invertebrates. J Innate Immun. 2016;8(2):129–42. 10.1159/000441475. 26580547 10.1159/000441475 PMC6738811

[224] Husemann J, Loike JD, Anankov R, Febbraio M, Silverstein SC. Scavenger receptors in neurobiology and neuropathology: their role on microglia and other cells of the nervous system. Glia. 2002;40(2):195–205. 10.1002/glia.10148. 12379907 10.1002/glia.10148

[225] Shi XZ, Yang MC, Kang XL, Li YX, Hong PP, Zhao XF, et al. Scavenger receptor B2, a type III membrane pattern recognition receptor, senses LPS and activates the IMD pathway in crustaceans. Proc Natl Acad Sci USA. 2023;120(24):e2216574120. 10.1073/pnas.2216574120. 37276415 10.1073/pnas.2216574120 PMC10268257

[226] Cuvillier-Hot V, Boidin-Wichlacz C, Slomianny C, Salzet M, Tasiemski A. Characterization and immune function of two intracellular sensors, HmTLR1 and HmNLR, in the injured CNS of an invertebrate. Dev Comp Immunol. 2011;35(2):214–26. 10.1016/j.dci.2010.09.011. 20920526 10.1016/j.dci.2010.09.011

[227] Kong X, Yuan Z, Cheng J. The function of NOD-like receptors in central nervous system diseases. J Neurosci Res. 2017;95(8):1565–73. 10.1002/jnr.24004. 28029680 10.1002/jnr.24004

[228] Tavolieri MV, Young SD, Bitzan M. A nod to gut–brain signalling: nod-like receptors are critical for gut–brain axis signalling in mice. J Physiol. 2020;598(5):907–8. 10.1113/JP279432. 31925784 10.1113/JP279432

[229] Takagi T, Yoshioka Y, Zayasu Y, Satoh N, Shinzato C. Transcriptome analyses of immune system behaviors in primary polyp of coral *Acropora digitifera* exposed to the bacterial pathogen *Vibrio coralliilyticus* under thermal loading. Mar Biotechnol. 2020;22(6):748–59. 10.1007/s10126-020-09984-1. 10.1007/s10126-020-09984-1 32696240

[230] Roesel CL, Vollmer SV. Differential gene expression analysis of symbiotic and aposymbiotic *Exaiptasia* anemones under immune challenge with *Vibrio coralliilyticus*. Ecol Evol. 2019;9(14):8279–93. 10.1002/ece3.5403. 31380089 10.1002/ece3.5403 PMC6662555

[231] Wang Y, Wang Q, Chen L, Li B. The lysosome-phagosome pathway mediates immune regulatory mechanisms in* Mesocentrotus nudus* against* Vibrio coralliilyticus* infection. Fish Shellfish Immunol. 2023;139:108864. 10.1016/j.fsi.2023.108864. 37277051 10.1016/j.fsi.2023.108864

[232] Bäumler AJ, Sperandio V. Interactions between the microbiota and pathogenic bacteria in the gut. Nature. 2016;535(7610):85–93. 10.1038/nature18849. 27383983 10.1038/nature18849 PMC5114849

[233] Martins R, Carlos AR, Braza F, Thompson JA, Bastos-Amador P, Ramos S, et al. Disease tolerance as an inherent component of immunity. Annu Rev Immunol. 2019;37(1):405–37. 10.1146/annurev-immunol-042718-041739. 30673535 10.1146/annurev-immunol-042718-041739

[234] Snell K. Enzymes of serine metabolism in normal, developing and neoplastic rat tissues. Adv Enzyme Regul. 1984;22:325–400. 10.1016/0065-2571(84)90021-9. 6089514 10.1016/0065-2571(84)90021-9

[235] Nelson DL, Cox MM, Nelson DL. Lehninger principles of biochemistry. Sixth edition. Lehninger AL, editor. Basingstoke: Macmillan Higher Education; 2013.

[236] Bertucci A, Moya A, Tambutté S, Allemand D, Supuran CT, Zoccola D. Carbonic anhydrases in anthozoan corals—a review. Bioorg Med Chem. 2013;21(6):1437–50. 10.1016/j.bmc.2012.10.024. 23199478 10.1016/j.bmc.2012.10.024

[237] Peng G, Shi X, Kadowaki T. Evolution of TRP channels inferred by their classification in diverse animal species. Mol Phylogenet Evol. 2015;84:145–57. 10.1016/j.ympev.2014.06.016. 24981559 10.1016/j.ympev.2014.06.016

[238] Vriens J, Nilius B, Voets T. Peripheral thermosensation in mammals. Nat Rev Neurosci. 2014;15(9):573–89. 10.1038/nrn3784. 25053448 10.1038/nrn3784

[239] Duruz J, Kaltenrieder C, Ladurner P, Bruggmann R, Martìnez P, Sprecher SG, et al. Acoel single-cell transcriptomics: cell type analysis of a deep branching bilaterian. Mol Biol Evol. 2021;38(5):1888–904. 10.1093/molbev/msaa333. 33355655 10.1093/molbev/msaa333 PMC8097308

[240] Cerenius L, Kawabata S, Lee BL, Nonaka M, Söderhäll K. Proteolytic cascades and their involvement in invertebrate immunity. Trends Biochem Sci. 2010;35(10):575–83. 10.1016/j.tibs.2010.04.006. 20541942 10.1016/j.tibs.2010.04.006

[241] Gorman MJ, Paskewitz SM. Serine proteases as mediators of mosquito immune responses. Insect Biochem Mol Biol. 2001;31(3):257–62. 10.1016/S0965-1748(00)00145-4. 11167095 10.1016/s0965-1748(00)00145-4

[242] Johnston PR, Makarova O, Rolff J. Inducible defenses stay up late: temporal patterns of immune gene expression in *Tenebrio molitor*. G3 Genes|Genomes|Genetics. 2014;4(6):947–55. 10.1534/g3.113.008516. 10.1534/g3.113.008516 PMC4065263 24318927

[243] Wirschell M, Olbrich H, Werner C, Tritschler D, Bower R, Sale WS, et al. The nexin-dynein regulatory complex subunit DRC1 is essential for motile cilia function in algae and humans. Nat Genet. 2013;45(3):262–8. 10.1038/ng.2533. 23354437 10.1038/ng.2533 PMC3818796

[244] Kimber SJ, Spanswick C. Blastocyst implantation: the adhesion cascade. Semin Cell Dev Biol. 2000;11(2):77–92. 10.1006/scdb.2000.0154. 10873705 10.1006/scdb.2000.0154

[245] Ho MSP, Böse K, Mokkapati S, Nischt R, Smyth N. Nidogens—extracellular matrix linker molecules. Microsc Res Tech. 2008;71(5):387–95. 10.1002/jemt.20567. 18219668 10.1002/jemt.20567

[246] Warner SC, Waite JH. Expression of multiple forms of an adhesive plaque protein in an individual mussel, Mytilus edulis. Mar Biol. 1999;134(4):729–34. 10.1007/s002270050589.

[247] Sars Centre, University of Bergen. Transcriptome of Xenoturbella bocki, *ENA. *2016. SRX1343818

[248] Sars Centre, University of Bergen. Transcriptome of the nemertodermatid Meara stichopi, *ENA. *2016. SRX134381

[249] Abalde S, Tellgren-Roth C, Heintz J, Vinnere Pettersson O, Jondelius U. The draft genome of the microscopic *Nemertoderma westbladi* sheds light on the evolution of Acoelomorpha genomes. Front Genet. 2023;26(14):1244493. 10.3389/fgene.2023.1244493. 10.3389/fgene.2023.1244493 PMC10565955 37829276

[250] Gehrke AR, Neverett E, Luo YJ, Brandt A, Ricci L, Hulett RE, et al. Acoel genome reveals the regulatory landscape of whole-body regeneration. Science. 2019;363(6432):eaau6173. 10.1126/science.aau6173. 30872491 10.1126/science.aau6173

[251] Sars Centre, University of Bergen. Transcriptome of the acoel Isodiametra pulchra, *ENA. *2016. SRX1343817

[252] Lewin TD, Liao IJ-Y, Luo Y-J. Conservation of animal genome structure is the exception not the rule. 2024. 10.1101/2024.08.02.606322.

[253] Martinez P, Ustyantsev K, Biryukov M, Mouton S, Glasenburg L, Sprecher SG, et al. Genome assembly of the acoel flatworm *Symsagittifera roscoffensis* , a model for research on body plan evolution and photosymbiosis. G3. 2022;13:jkac336. 10.1093/g3journal/jkac336. 10.1093/g3journal/jkac336 PMC9911081 36542495

[254] Sars Centre, University of Bergen. Transcriptome of the acoel Convolutriloba macropyga, *ENA. *2016. SRX1343815

[255] Lu S, Wang J, Chitsaz F, Derbyshire MK, Geer RC, Gonzales NR, et al. CDD/SPARCLE: the conserved domain database in 2020. Nucleic Acids Res. 2020;48(D1):D265–8. 10.1093/nar/gkz991. 31777944 10.1093/nar/gkz991 PMC6943070

[256] Marchler-Bauer A, Bryant SH. CD-Search: protein domain annotations on the fly. Nucleic Acids Res. 2004;32:W327-331. 10.1093/nar/gkh454. 15215404 10.1093/nar/gkh454 PMC441592

[257] Marchler-Bauer A, Lu S, Anderson JB, Chitsaz F, Derbyshire MK, DeWeese-Scott C, et al. CDD: a Conserved Domain Database for the functional annotation of proteins. Nucleic Acids Res. 2011;39(Database issue):D225-229. 10.1093/nar/gkq1189. 21109532 10.1093/nar/gkq1189 PMC3013737

[258] Potter SC, Luciani A, Eddy SR, Park Y, Lopez R, Finn RD. HMMER web server: 2018 update. Nucleic Acids Res. 2018;46(W1):W200-4. 10.1093/nar/gky448. 29905871 10.1093/nar/gky448 PMC6030962

[259] Katoh K. MAFFT: a novel method for rapid multiple sequence alignment based on fast Fourier transform. Nucleic Acids Res. 2002;30(14):3059–66. 10.1093/nar/gkf436. 12136088 10.1093/nar/gkf436 PMC135756

[260] Katoh K, Standley DM. MAFFT multiple sequence alignment software version 7: improvements in performance and usability. Mol Biol Evol. 2013;30(4):772–80. 10.1093/molbev/mst010. 23329690 10.1093/molbev/mst010 PMC3603318

[261] Borowiec ML. AMAS: a fast tool for alignment manipulation and computing of summary statistics. PeerJ. 2016;4(28):e1660. 10.7717/peerj.1660. 26835189 10.7717/peerj.1660 PMC4734057

[262] Minh BQ, Schmidt HA, Chernomor O, Schrempf D, Woodhams MD, Von Haeseler A, et al. Iq-tree 2: new models and efficient methods for phylogenetic inference in the genomic era. Mol Biol Evol. 2020;37:1530–4. 10.1093/molbev/msaa015. 32011700 10.1093/molbev/msaa015 PMC7182206

[263] Chernomor O, Von Haeseler A, Minh BQ. Terrace aware data structure for phylogenomic inference from supermatrices. Syst Biol. 2016;65(6):997–1008. 10.1093/sysbio/syw037. 27121966 10.1093/sysbio/syw037 PMC5066062

[264] Kalyaanamoorthy S, Minh BQ, Wong TKF, Von Haeseler A, Jermiin LS. Modelfinder: fast model selection for accurate phylogenetic estimates. Nat Methods. 2017;14(6):587–9. 10.1038/nmeth.4285. 28481363 10.1038/nmeth.4285 PMC5453245

[265] Hoang DT, Chernomor O, Von Haeseler A, Minh BQ, Vinh LS. UFBoot2: improving the ultrafast bootstrap approximation. Mol Biol Evol. 2018;35(2):518–22. 10.1093/molbev/msx281. 29077904 10.1093/molbev/msx281 PMC5850222

[266] DSMZ: 1. Nutrient Agar. 2022. https://mediadive.dsmz.de/medium/1. Accessed 2 Jan 2025.

[267] Wahid MH, Eroglu E, LaVars SM, Newton K, Gibson CT, Stroeher UH, et al. Microencapsulation of bacterial strains in graphene oxide nano-sheets using vortex fluidics. RSC Adv. 2015;5(47):37424–30.

[268] Gerst R, Cseresnyés Z, Figge MT. JIPipe: visual batch processing for ImageJ. Nat Methods. 2023;20(2):168–9. 10.1038/s41592-022-01744-4. 36627450 10.1038/s41592-022-01744-4

[269] Ye J, Coulouris G, Zaretskaya I, Cutcutache I, Rozen S, Madden TL. Primer-BLAST: a tool to design target-specific primers for polymerase chain reaction. BMC Bioinformatics. 2012;13(1):134. 10.1186/1471-2105-13-134. 22708584 10.1186/1471-2105-13-134 PMC3412702

[270] Eu LC, Ong KC, Hiu J, Vadivelu J, Nathan S, Wong KT. In situ hybridization to detect and identify *Burkholderia pseudomallei* in human melioidosis. Mod Pathol. 2014;27(5):657–64. 10.1038/modpathol.2013.184. 24186135 10.1038/modpathol.2013.184

[271] Ozer EA. in_silico_pcr. GitHub; 2017. Available from: https://github.com/egonozer/in_silico_pcr.git

[272] Priyam A, Woodcroft BJ, Rai V, Moghul I, Munagala A, Ter F, et al. Sequenceserver: a modern graphical user interface for custom BLAST databases. Mol Biol Evol. 2019;36(12):2922–4. 10.1093/molbev/msz185. 31411700 10.1093/molbev/msz185 PMC6878946

[273] Altschul SF, Gish W, Miller W, Myers EW, Lipman DJ. Basic local alignment search tool. J Mol Biol. 1990;215(3):403–10. 10.1016/S0022-2836(05)80360-2. 2231712 10.1016/S0022-2836(05)80360-2

[274] Hejnol A. In situ protocol for embryos and juveniles of Convolutriloba longifissura. Protoc Exch. 2008. 10.1038/nprot.2008.201.

[275] Friedrich Schiller University Jena. Transcriptomic analysis of Convolutriloba macropyga immune challenged adults, control adults, and juveniles, *ENA. *2025. PRJNA1332086

[276] Grabherr MG, Haas BJ, Yassour M, Levin JZ, Thompson DA, Amit I, et al. Full-length transcriptome assembly from RNA-Seq data without a reference genome. Nat Biotechnol. 2011;29(7):644–52. 10.1038/nbt.1883. 21572440 10.1038/nbt.1883 PMC3571712

[277] Chen S, Zhou Y, Chen Y, Gu J. Fastp: an ultra-fast all-in-one FASTQ preprocessor. Bioinformatics. 2018;34(17):i884-90. 10.1093/bioinformatics/bty560. 30423086 10.1093/bioinformatics/bty560 PMC6129281

[278] Patro R, Duggal G, Love MI, Irizarry RA, Kingsford C. Salmon provides fast and bias-aware quantification of transcript expression. Nat Methods. 2017;14(4):417–9. 10.1038/nmeth.4197. 28263959 10.1038/nmeth.4197 PMC5600148

[279] R Core Team. R: a language and environment for statistical computing. Vienna, Austria: R Foundation for Statistical Computing; 2023.

[280] Love MI, Soneson C, Patro R. Swimming downstream: statistical analysis of differential transcript usage following Salmon quantification. F1000Res. 2018;7:952. 10.12688/f1000research.15398.3. 30356428 10.12688/f1000research.15398.1 PMC6178912

[281] Soneson C, Love MI, Robinson MD. Differential analyses for RNA-seq: transcript-level estimates improve gene-level inferences. F1000Res. 2015;4:1521. 10.12688/f1000research.7563.1. 26925227 10.12688/f1000research.7563.1 PMC4712774

[282] Wang J, Chitsaz F, Derbyshire MK, Gonzales NR, Gwadz M, Lu S, et al. The conserved domain database in 2023. Nucleic Acids Res. 2023;51(D1):D384–8. 10.1093/nar/gkac1096. 36477806 10.1093/nar/gkac1096 PMC9825596

[283] Bethesda (MD): National Center for Biotechnology Information (US). BLAST® Command Line Applications User Manual. 2008. Available from: https://www.ncbi.nlm.nih.gov/books/NBK279670/

[284] Pinton F. acoel-immune-signalling-pathways-immune-challenges, *GitHub. *2025. 10.5281/zenodo.17535040.

[285] Wickham H. ggplot2: elegant graphics for data analysis. Second edition. Cham: Springer international publishing; 2016.

[286] Yu G, Smith DK, Zhu H, Guan Y, Lam TT, McInerny G. Ggtree : an r package for visualization and annotation of phylogenetic trees with their covariates and other associated data. Methods Ecol Evol. 2017;8(1):28–36. 10.1111/2041-210X.12628.

[287] Wang LG, Lam TTY, Xu S, Dai Z, Zhou L, Feng T, et al. Treeio: an R package for phylogenetic tree input and output with richly annotated and associated data. Mol Biol Evol. 2020;37(2):599–603. 10.1093/molbev/msz240. 10.1093/molbev/msz240 PMC6993851 31633786

[288] Schindelin J, Arganda-Carreras I, Frise E, Kaynig V, Longair M, Pietzsch T, et al. Fiji: an open-source platform for biological-image analysis. Nat Methods. 2012;9(7):676–82. 10.1038/nmeth.2019. 22743772 10.1038/nmeth.2019 PMC3855844

[289] Therneau TM. coxme: mixed effects cox models. 2024. Available from: https://CRAN.R-project.org/package=coxme

[290] Crawley MJ. The R book. 2nd ed. Chichester, West Sussex, UK: Wiley; 2013.

[291] Phillips N. YaRrr! The pirate’s guide to R. 2024. Available from: https://bookdown.org/ndphillips/YaRrr/

[292] Fox J, Weisberg S. An R companion to applied regression. 3rd ed. Thousand Oaks CA: Sage; 2019. Available from: https://www.john-fox.ca/Companion/

[293] Lenth RV. emmeans: estimated marginal means, aka least-squares means. 2025. Available from: https://rvlenth.github.io/emmeans/

[294] Kassambara A, Kosinski M, Biecek P. survminer: drawing survival curves using ‘ggplot2’. 2024. Available from: https://github.com/kassambara/survminer

[295] Hothorn T, Bretz F, Westfall P. Simultaneous inference in general parametric models. Biom J. 2008;50(3):346–63. 18481363 10.1002/bimj.200810425

[296] Brooks ME, Kristensen K, Benthem KJ, van, Magnusson A, Berg C W, Nielsen A, et al. glmmTMB balances speed and flexibility among packages for zero-inflated generalized linear mixed modeling. R J. 2017;9(2):378. 10.32614/RJ-2017-066.

[297] Akaike H. Information Theory and an Extension of the Maximum Likelihood Principle. In: Parzen E, Tanabe K, Kitagawa G, editors. Selected Papers of Hirotugu Akaike. New York, NY: Springer New York; 1998. p. 199–213. 10.1007/978-1-4612-1694-0_15.

[298] Burnham KP, Anderson DR. Multimodel inference: understanding AIC and BIC in model selection. Sociol Methods Res. 2004;33(2):261–304. 10.1177/0049124104268644.

