# Supplementary material for: Absence of conserved immune signalling pathways and increased pathogen susceptibility associated to photosymbiosis in acoels
Source: BMC Biol. 2026 Jan 23;24:25. doi: 10.1186/s12915-026-02506-w (PMC12849515; doi:10.1186/s12915-026-02506-w)
Supplement: Supplementary file 1 — Additional File 1: Fig. S1-S10. [file 12915_2026_2506_MOESM1_ESM.pdf]

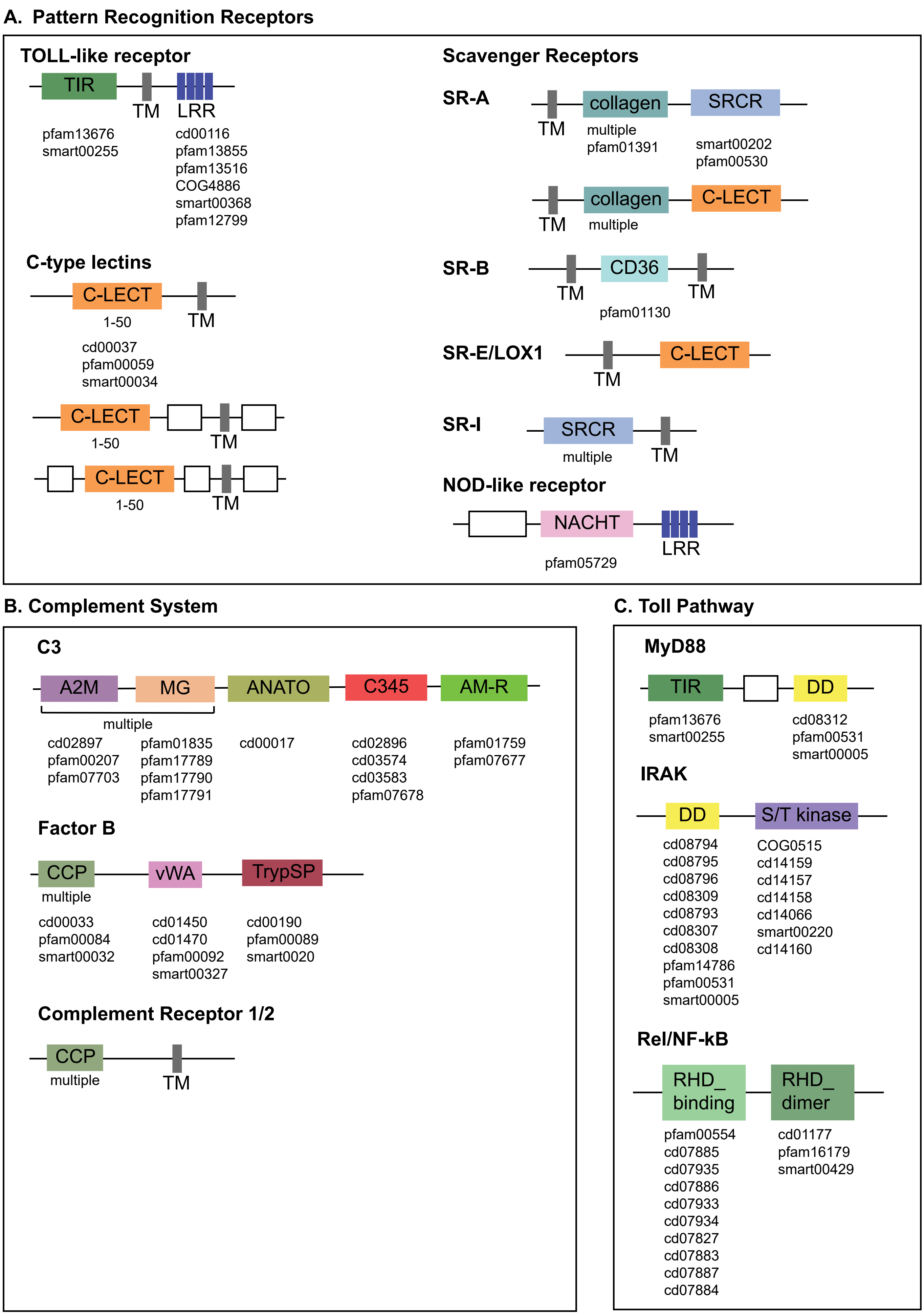
A. Pattern Recognition Receptors
TOLL-like receptor
TIR
TM
LRR
pfam13676
smart00255
cd00116
pfam13855
pfam13516
COG4886
smart00368
pfam12799
C-type lectins
C-LECT
1-50
TM
cd00037
pfam00059
smart00034
C-LECT
1-50
TM
C-LECT
1-50
TM
Scavenger Receptors
SR-A
TM
collagen
SRCR
multiple
pfam01391
smart00202
pfam00530
TM
collagen
C-LECT
multiple
SR-B
TM
CD36
TM
pfam01130
SR-E/LOX1
TM
C-LECT
SR-I
SRCR
multiple
TM
NOD-like receptor
NACHT
LRR
pfam05729
B. Complement System
C3
A2M
MG
ANATO
C345
AM-R
multiple
cd02897
pfam00207
pfam07703
pfam01835
pfam17789
pfam17790
pfam17791
cd00017
cd02896
cd03574
cd03583
pfam07678
pfam01759
pfam07677
Factor B
CCP
vWA
TrypSP
multiple
cd00033
pfam00084
smart00032
cd01450
cd01470
pfam00092
smart00327
cd00190
pfam00089
smart0020
Complement Receptor 1/2
CCP
multiple
TM
C. Toll Pathway
MyD88
TIR
DD
pfam13676
smart00255
cd08312
pfam00531
smart00005
IRAK
DD
S/T kinase
cd08794
cd08795
cd08796
cd08309
cd08793
cd08307
cd08308
pfam14786
pfam00531
smart00005
COG0515
cd14159
cd14157
cd14158
cd14066
smart00220
cd14160
Rel/NF-kB
RHD_ binding
RHD_ dimer
pfam00554
cd07885
cd07935
cd07886
cd07933
cd07934
cd07827
cd07883
cd07887
cd07884
cd01177
pfam16179
smart00429

Fig. S1 – Gene domain structures searched in xenacoelomorph predicted proteomes

Schematic domain structure of the genes searched in xenacoelomorph genomes and transcriptomes: **A** Pattern Recognition Receptors, **B** Toll Pathway, **C** Complement system. Under each domain name, the number of times they can be repeated (if higher than one) and the accession numbers from NCBI Conserved Domain Database used to build the hmm profiles and to filter results.

A2M = α2-macroglobulin domain; AM-R = alpha-macroglobulin binding domain; ANATO = anaphylatoxin domain; C345C = C-terminal domain specific to C3, C4, and C5; CCP = complement control protein domain; CD36 = cluster of differentiation 36; CLECT = C-type lectin domain; DD = Death Domain; IRAK = Interleukin-1 receptor-associated kinases; LRR = Leucine-rich repeat; MG = macroglobulin domain; MyD88 = Myeloid differentiation factor 88; NF-κB = Nuclear Factor-κB; RHD_bind = Rel homology DNA-binding domain; RHD_dimer = Rel homology dimerization domain (immunoglobulin-plexin-transcription domain); SR = Scavenger Receptor; SRCR = scavenger receptor cysteine rich domain; TIR = Toll/IL-1 receptor domain; TM = transmembrane region; TrypSP = trypsin-like serine protease domain; vWA = von Willebrand factor type A domain

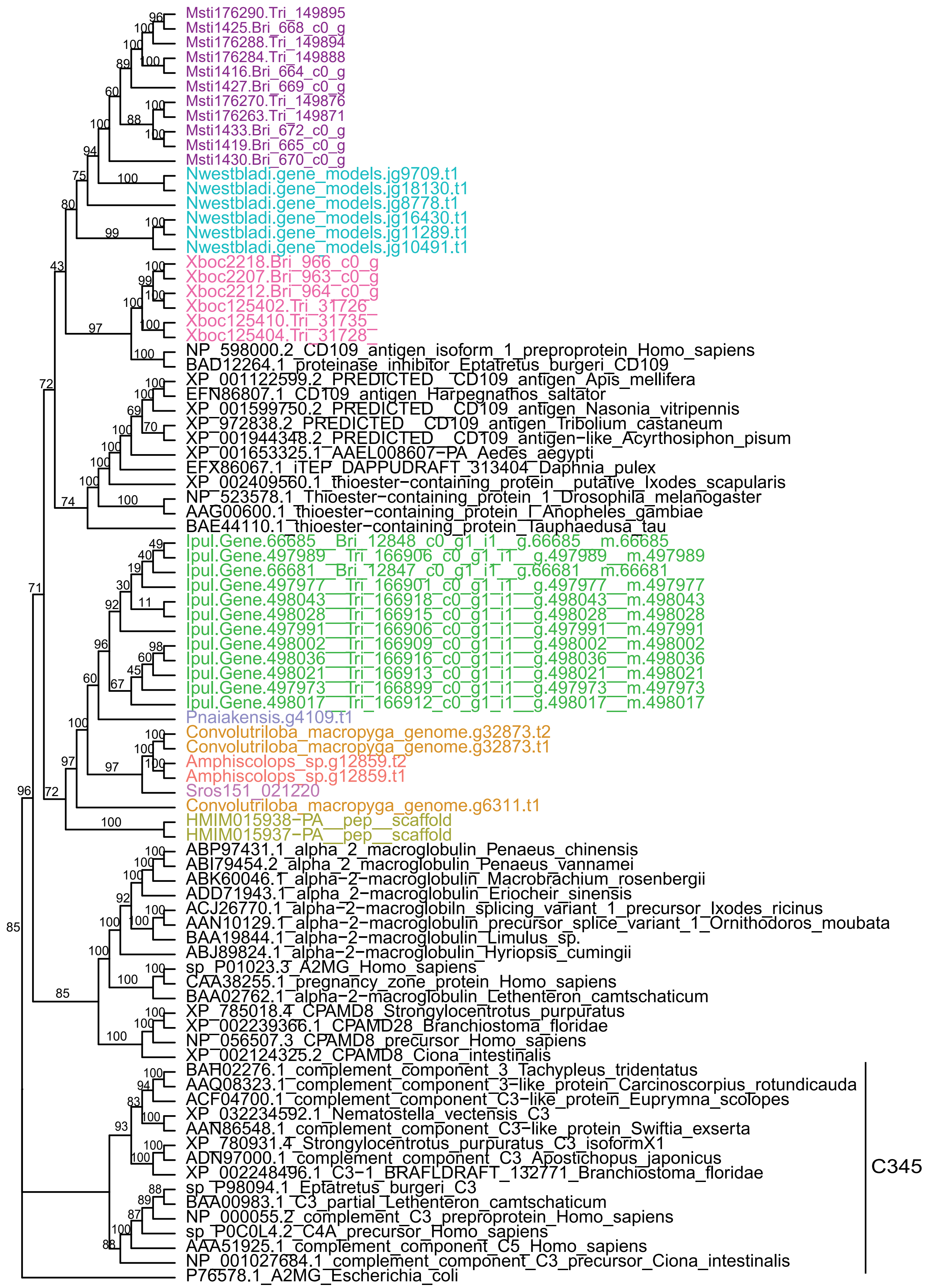

Fig. S2 – C3 phylogeny

Maximum likelihood phylogeny of domains in C3 and related proteins. UltraFast Bootstrap support values are shown.

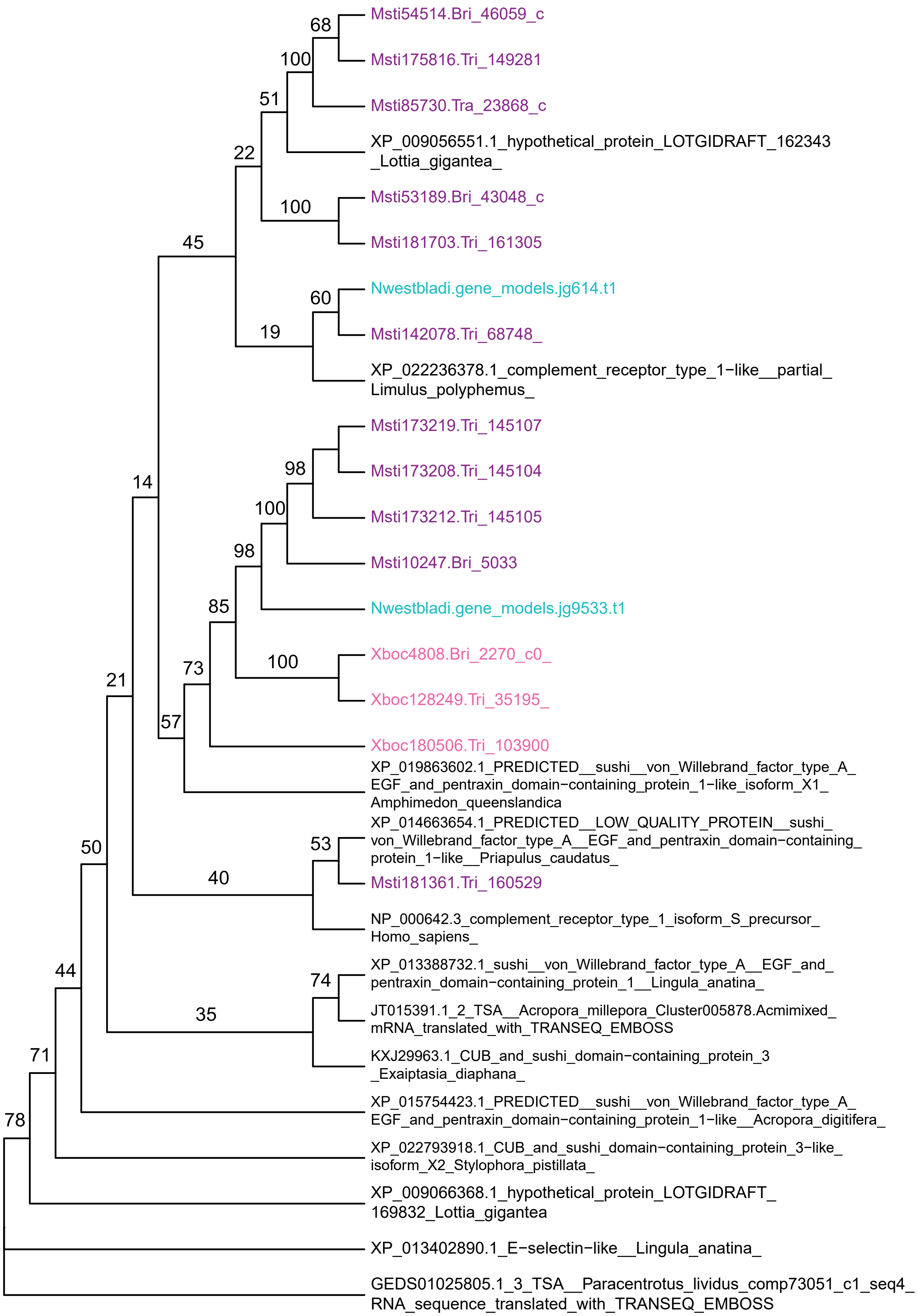

Fig. S3 – CR1/2 phylogeny
Maximum likelihood phylogeny of domains in Complement receptors 1/2 and related proteins. Ultra-Fast Bootstrap support values are shown.

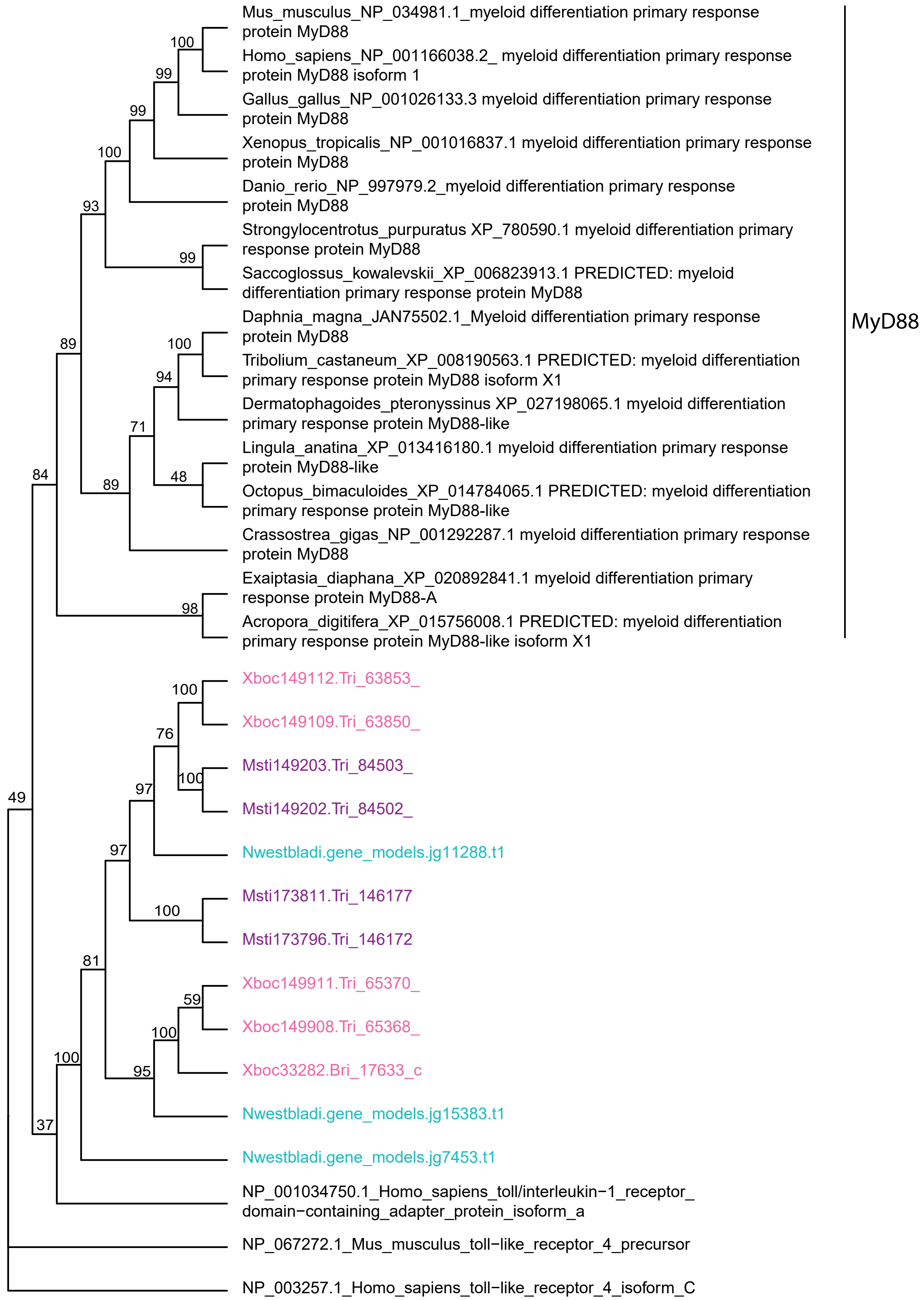

Fig. – S4 MyD88 phylogeny

Maximum likelihood phylogeny of domains in MyD88 and TIR-containing proteins as outgroup. UltraFast Bootstrap support values are shown.

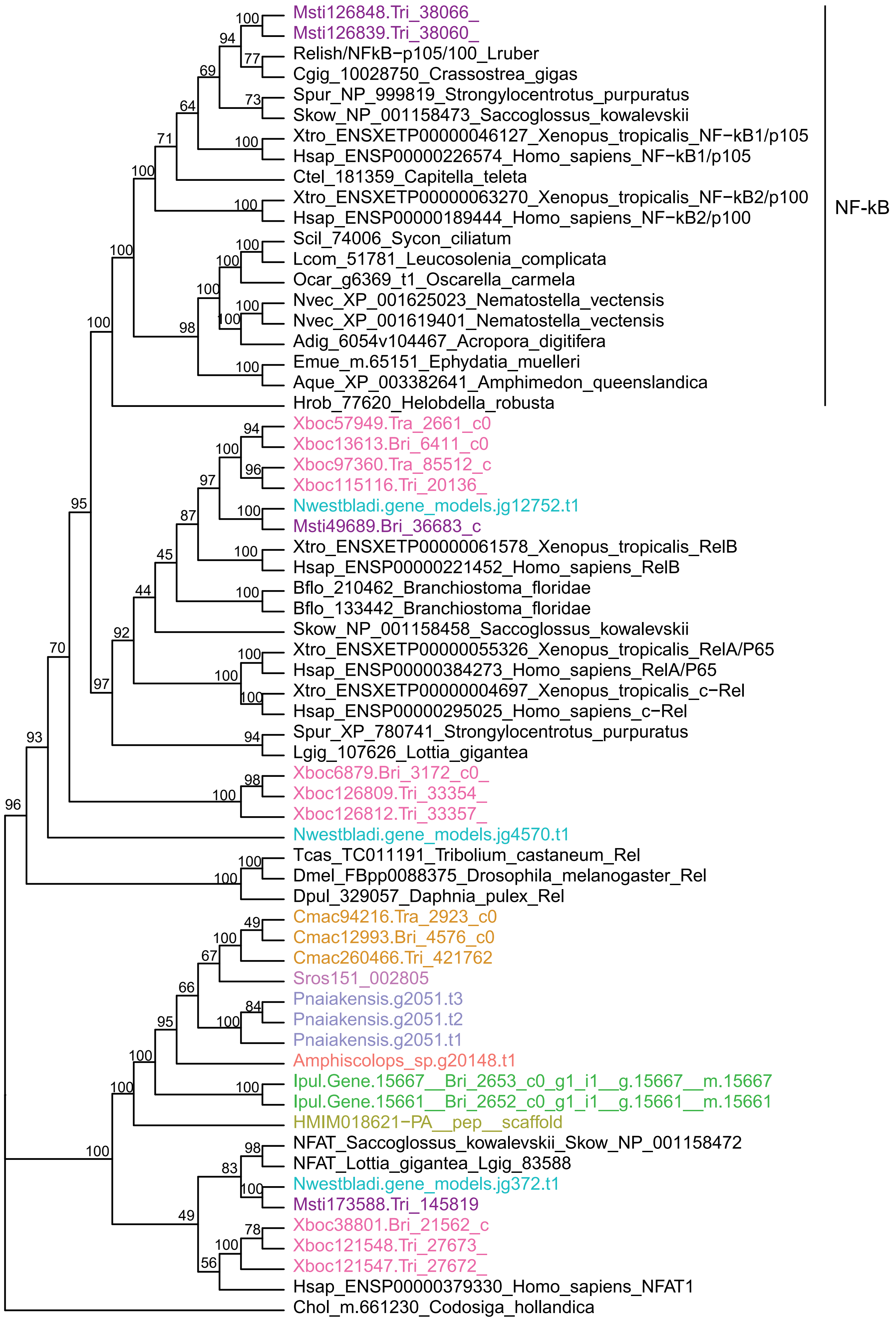
Msti126848.Tri_38066_
Msti126839.Tri_38060_
Relish/NFkB−p105/100_Lruber
Cgig_10028750_Crassostrea_gigas
Spur_NP_999819_Strongylocentrotus_purpuratus
Skow_NP_001158473_Saccoglossus_kowalevskii
Xtro_ENSXETP00000046127_Xenopus_tropicalis_NF−kB1/p105
Hsap_ENSP00000226574_Homo_sapiens_NF−kB1/p105
Ctel_181359_Capitella_teleta
Xtro_ENSXETP00000063270_Xenopus_tropicalis_NF−kB2/p100
Hsap_ENSP00000189444_Homo_sapiens_NF−kB2/p100
Scil_74006_Sycon_ciliatum
Lcom_51781_Leucosolenia_complicata
Ocar_g6369_t1_Oscarella_carmela
Nvec_XP_001625023_Nematostella_vectensis
Nvec_XP_001619401_Nematostella_vectensis
Adig_6054v104467_Acropora_digitifera
Emue_m.65151_Ephydatia_muelleri
Aque_XP_003382641_Amphimedon_queenslandica
Hrob_77620_Helobdella_robusta
NF-kB
Xboc57949.Tra_2661_c0
Xboc13613.Bri_6411_c0
Xboc97360.Tra_85512_c
Xboc115116.Tri_20136_
Nwestbladi.gene_models.jg12752.t1
Msti49689.Bri_36683_c
Xtro_ENSXETP00000061578_Xenopus_tropicalis_RelB
Hsap_ENSP00000221452_Homo_sapiens_RelB
Bflo_210462_Branchiostoma_floridae
Bflo_133442_Branchiostoma_floridae
Skow_NP_001158458_Saccoglossus_kowalevskii
Xtro_ENSXETP00000055326_Xenopus_tropicalis_RelA/P65
Hsap_ENSP00000384273_Homo_sapiens_RelA/P65
Xtro_ENSXETP00000004697_Xenopus_tropicalis_c−Rel
Hsap_ENSP00000295025_Homo_sapiens_c−Rel
Spur_XP_780741_Strongylocentrotus_purpuratus
Lgig_107626_Lottia_gigantea
Xboc6879.Bri_3172_c0_
Xboc126809.Tri_33354_
Xboc126812.Tri_33357_
Nwestbladi.gene_models.jg4570.t1
Tcas_TC011191_Tribolium_castaneum_Rel
Dmel_FBpp0088375_Drosophila_melanogaster_Rel
Dpul_329057_Daphnia_pulex_Rel
Cmac94216.Tra_2923_c0
Cmac12993.Bri_4576_c0
Cmac260466.Tri_421762
Sros151_002805
Pnaiakensis.g2051.t3
Pnaiakensis.g2051.t2
Pnaiakensis.g2051.t1
Amphiscolops_sp.g20148.t1
Ipul.Gene.15667__Bri_2653_c0_g1_i1__g.15667__m.15667
Ipul.Gene.15661__Bri_2652_c0_g1_i1__g.15661__m.15661
HMIM018621−PA__pep__scaffold
NFAT_Saccoglossus_kowalevskii_Skow_NP_001158472
NFAT_Lottia_gigantea_Lgig_83588
Nwestbladi.gene_models.jg372.t1
Msti173588.Tri_145819
Xboc38801.Bri_21562_c
Xboc121548.Tri_27673_
Xboc121547.Tri_27672_
Hsap_ENSP00000379330_Homo_sapiens_NFAT1
Chol_m.661230_Codosiga_hollandica

Fig. S5 – NF-κB phylogeny

Maximum likelihood phylogeny of domains in NFκB and related proteins. UltraFast Bootstrap support values are shown.

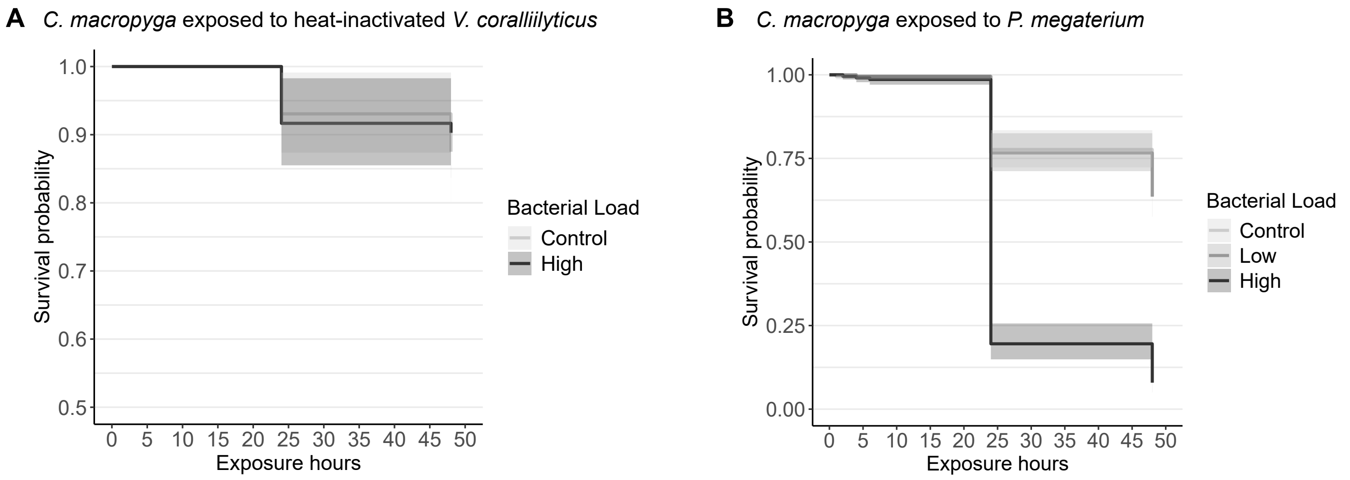

Fig. S6 – *C. macropyga* survival upon exposure to heat-inactivated *Vibrio coralliilyticus* and to *Priestia megaterium*

Survival curves with 95% confidence intervals for *C. macropyga* adults immune challenged with: **A** heat-inactivated *V. coralliilyticus* (minimal adequate Cox proportional hazards model Surv(last.obs,censored) ~ 1, n = 145) or **B** *P. megaterium* (minimal adequate Cox proportional hazards model Surv(last.obs,censored) ~ Bacterial.Load + (1 | batch), X^2=252.3, p = 2.2e-16, n= 644). Full statis-tics in Additional File 4: Table S2.

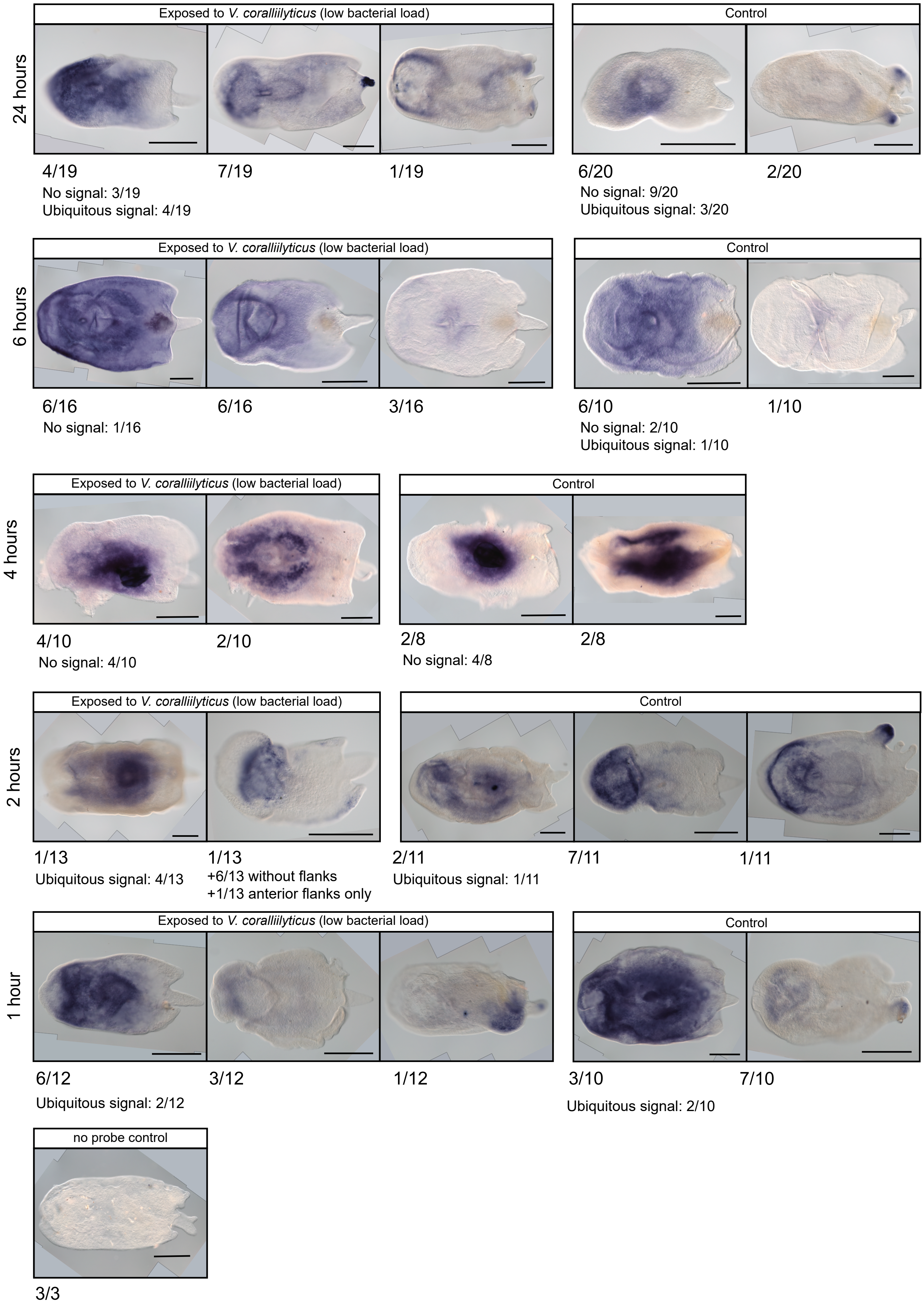
24 hours
Exposed to V. coralliilyticus (low bacterial load)
4/19
No signal: 3/19
Ubiquitous signal: 4/19
7/19
1/19
Control
6/20
No signal: 9/20
Ubiquitous signal: 3/20
2/20
6 hours
Exposed to V. coralliilyticus (low bacterial load)
6/16
No signal: 1/16
6/16
3/16
Control
6/10
No signal: 2/10
Ubiquitous signal: 1/10
1/10
4 hours
Exposed to V. coralliilyticus (low bacterial load)
4/10
No signal: 4/10
2/10
Control
2/8
No signal: 4/8
2/8
2 hours
Exposed to V. coralliilyticus (low bacterial load)
1/13
Ubiquitous signal: 4/13
1/13
+6/13 without flanks
+1/13 anterior flanks only
Control
2/11
Ubiquitous signal: 1/11
7/11
1/11
1 hour
Exposed to V. coralliilyticus (low bacterial load)
6/12
Ubiquitous signal: 2/12
3/12
1/12
Control
3/10
Ubiquitous signal: 2/10
7/10
no probe control
3/3

Fig. S7 – In situ hybridisation against *Vibrio coralliilyticus* 16S rRNA in *C. macropyga* at various times of bacterial exposure

RNA in situ hybridisation against *V. coralliilyticus* 16S in *C. macropyga* exposed to a low load of *V. coralliilyticus* or control medium for 1, 2, 4, 6, or 24 hours. Numbers indicate the ratios of individuals with the pattern shown above; dorsal view, anterior is facing left. Scale bars are 0.5 mm.

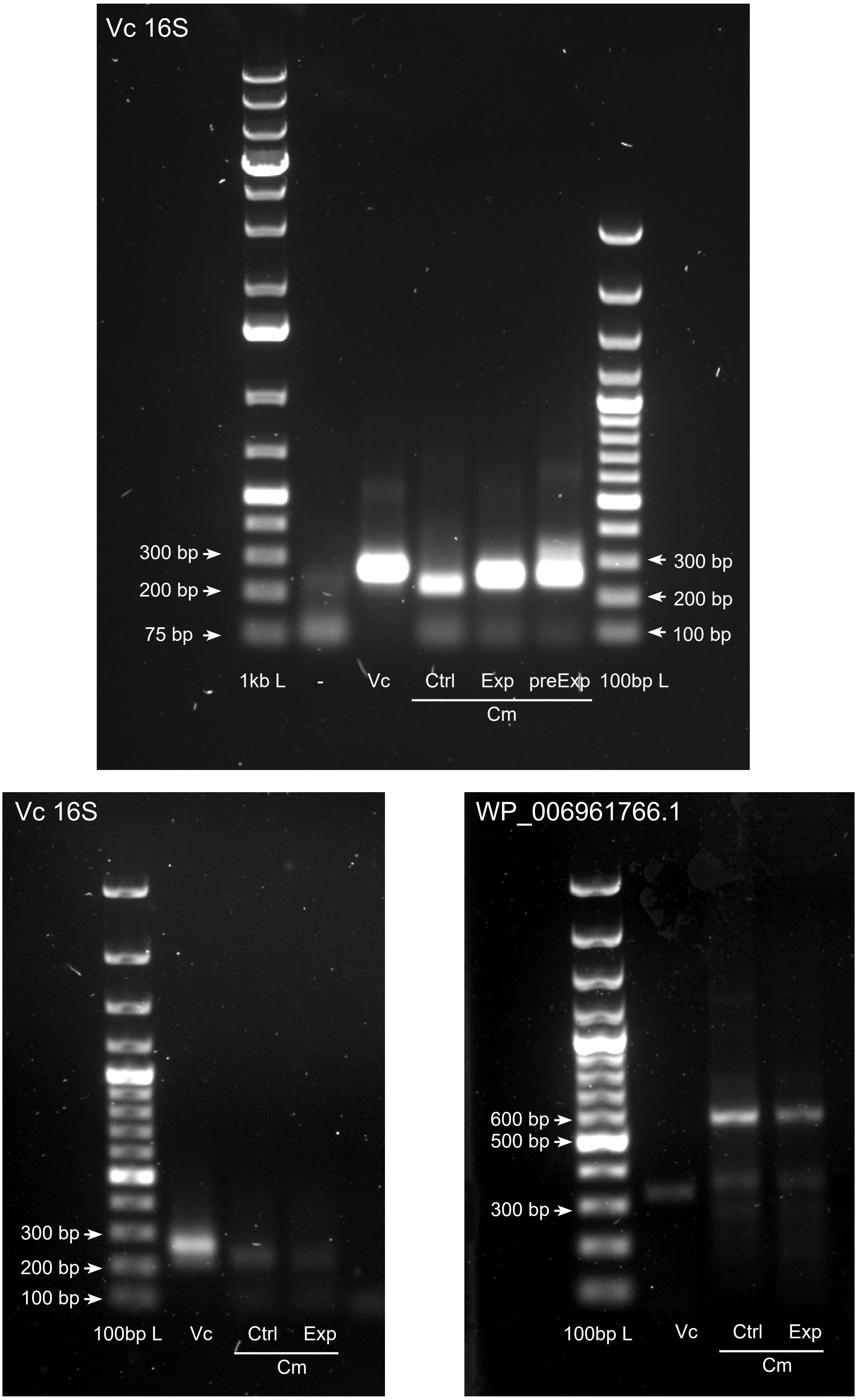

Fig. S8 – PCR for *V. coralliilyticus* 16S rRNA and virulence factor WP_006961766.1
Gel electrophoresis for PCR amplification with primers for *V. coralliilyticus* 16S and *V. coralliilyticus* virulence factor WP_006961766.1 from *V. coralliilyticus* and *C. macropyga* cDNA. As template: DNAse and RNAse free water as negative control (-), *V. coralliilyticus* cDNA as positive control (Vc), cDNA from unexposed *C. macropyga* (Ctrl), from *C. macropyga* exposed to a low dose of *V. coralliilyticus* (Exp), and *C. macropyga* cDNA synthesized before acquiring the bacterial cultures (preExp). The ladders are GeneRuler 1kb Plus DNA ladder (1kb L) and GeneRuler 100 bp Plus DNA ladder (100bp L) from Thermo Fisher Scientific (Waltham, MA, USA).

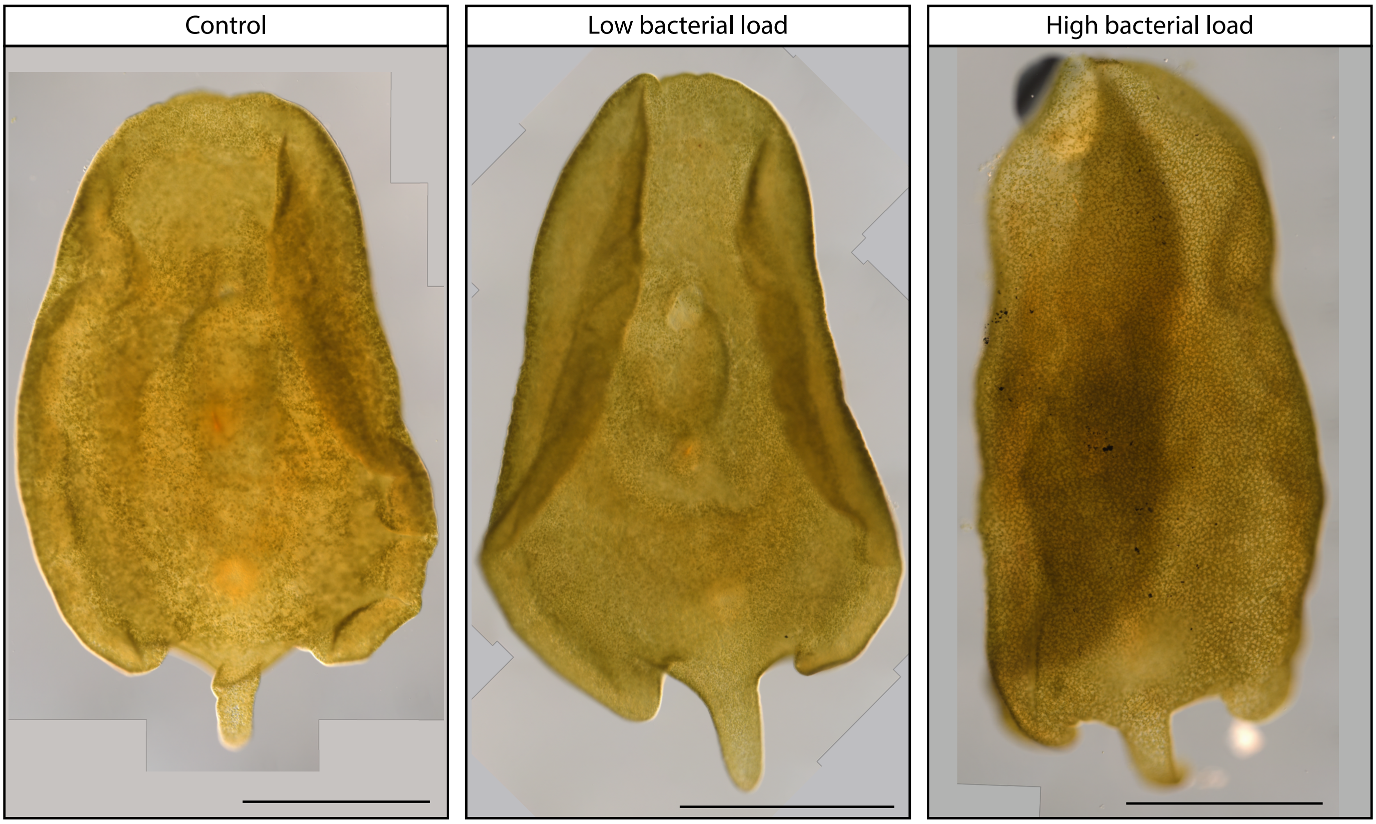

Fig. S9 – *Convolutriloba macropyga* exposed for 14 days to *Vibrio coralliilyticus*
C. macropyga after 14-days immune challenges with *V. coralliilyticus* (low dose, high dose, and control), imaged with DIC after fixation and mounting. Anterior facing upwards, ventral view.

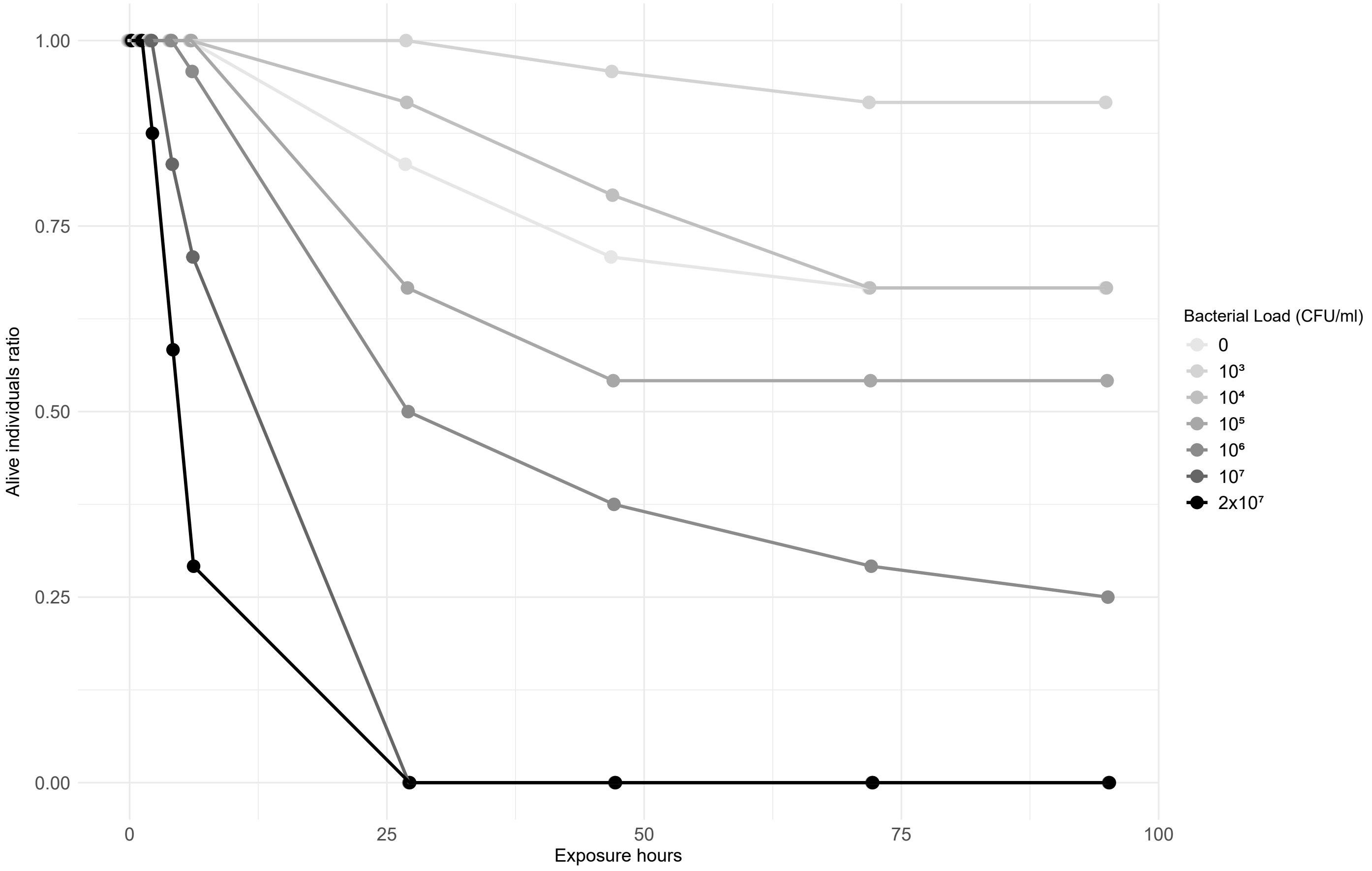

Fig. S10 – *Convolutriloba macropyga* exposed to different bacterial loads of *Vibrio coralliilyticus*

Survival curves of *C. macropyga* exposed to various loads of *V. coralliilyticus* for 96 hours. 24 individuals were immune challenged at each bacterial load.
