# Supplementary material for: Absence of conserved immune signalling pathways and increased pathogen susceptibility associated to photosymbiosis in acoels
Source: BMC Biol. 2026 Jan 23;24:25. doi: 10.1186/s12915-026-02506-w (PMC12849515; doi:10.1186/s12915-026-02506-w)
Supplement: Supplementary file 4 — Additional File 4: Table S2-S4. Survival, dysbiosis, and reproduction statistics. [file 12915_2026_2506_MOESM4_ESM.docx]

**Table S2 – Survival statistics**

Summary of statistical model characteristics and outputs used in the analysis of survival of *C. macropyga* adults upon immune challenge with *V. coralliilyticus* and *P. megaterium*. Model: Cox proportional hazards mixed-effects model (R *coxme* package). Post-hoc comparisons: pairwise comparisons of estimated marginal means, with Bonferroni correction for P value (R *emmeans* package). Degrees of freedom were infinite for all comparisons.

| Species, stage | *C. macropyga* adults | *C. macropyga* adults | |
| --- | --- | --- | --- |
| Immune challenge agent | *V. coralliilyticus* | *P. megaterium* | |
| Response variable | Surv(last.obs,censored) | Surv(last.obs,censored) | |
| Number of replicates | 4 | 3 | |
| Sample size | 863 | 644 | |
| Maximal model | ~ Bacterial.Load * initial.damage + (1 \| batch / id ) | ~ Bacterial.Load * initial.damage + (1 \| batch / id ) | |
| Minimal model | ~ Bacterial.Load + (1 \| batch) | ~ Bacterial.Load + (1 \| batch) | |
| Anova (Type II) | Χ^2^=59.742, p = 1.065e-13 | Χ^2^=252.3, p = 2.2e-16 | |
| Fixed effects | |  | |
| Bacterial load 10^5^ CFUs | HR ± SE = 2.2293 ± 0.2268, z = 3.53, p = 0.000409 | HR ± SE = 0.100 ± 0.160, z = 0.00, p = 1 |  |
| Bacterial load 10^6^ CFUs | HR ± SE = 4.6465 ± 0.2104, z = 7.30, p = 2.85e-13 | HR ± SE = 6.421 ± 0.142, z = 13.13, p = 0 |  |
| Random effects (Variable = intercept) | |  | |
| Batch | SD = 0.6924, Var = 0.4794598 | SD = 0.500, Var = 0.250 |  |
| Post hoc comparisons | |  | |
| Control /10^5^ CFUs | ratio = 0.449, SE = 0.1020, z ratio = -3.534, p = 0.0012 | ratio = 1.000, SE = 0.160, z ratio = 0.003, p = 1 |  |
| Control / 10^6^ CFUs | ratio = 0.215, SE = 0.0453, z ratio -7.301, p <.0001 | ratio = 0.156, SE = 0.0221, z ratio = -13.126, p <.0001 |  |
| 10^5^ CFUs / 10^6^ CFUs | ratio = 0.480, SE = 0.0783, z ratio = -4.500, p <.0001 | ratio = 0.156, SE = 0.0221, z ratio = -13.097, p <.0001 |  |

**Table S3 – Dysbiosis statistics**

Summary of statistical model characteristics and outputs used in the analysis of the ratio between algal cells and animal cells in *C. macropyga* adults upon immune challenge with *V. coralliilyticus*. Model: linear model (R *lm* function). Sample size represents the number of individuals imaged. Post hoc Tukey’s tests were carried out with the R function *glht* from the *multcomp* package.

| Species, stage | *C. macropyga* adults |
| --- | --- |
| Immune challenge agent | *V. coralliilyticus* |
| Exposure | 48 hours |
| Response variable | Ratio.algae.hoechst |
| Number of replicates | 1 |
| Sample size | 74 |
| Maximal model | ~ Bacterial.Load * Length * Orientation |
| Minimal model | ~ Bacterial.Load |
| Anova (Type II) | F = 5.5069, p = 0.006961 |
| Tukey HSD Contrasts | |
| Low bact. load - control | t = -1.348, p = 0.37601 |
| High bact. load - control | t = -3.298, p = 0.00519 |
| High bact. load – low bact. load | t = -1.861, p = 0.16080 |

**Table S4 – Reproduction statistics**

Summary of statistical model characteristics and outputs used in the analysis of asexual progeny release of *C. macropyga* adults upon immune challenge with *V. coralliilyticus*. Model: generalized linear mixed-effects model (R *glmTMB* package). Estimated marginal means (EMMs) obtained with R *emmeans* package (specs = ~ bud, type = “response”). Sample size corresponds to the number of alive individuals at the corresponding day.

| Species, stage | *C. macropyga* adults | *C. macropyga* adults | |
| --- | --- | --- | --- |
| Immune challenge agent | *V. coralliilyticus* | *V. coralliilyticus* | |
| Exposure day | Day 1 | Day 2 | |
| Response variable | progeny.released | progeny.released | |
| Distribution function | Poisson | Poisson | |
| Number of replicates | 4 | 4 | |
| Sample size | 813 | 669 | |
| Maximal model | ~ Bacterial.Load * bud + ( 1 \| batch) | ~ Bacterial.Load * bud + ( 1 \| batch) | |
| Minimal model | ~ bud + (1 \| batch) | ~ bud | |
| Anova (Type II) | Χ^2^ = 24.684, p = 6.752e-07 | Χ^2^ = 18.512, p = 1.688e-05 | |
| Fixed effects (EMMs) | |  | |
| Bud absent | rate ± SD = 0.171 ± 0.0266 | rate ± SD = 0.108 ± 0.0136 |  |
| Bud present | rate ± SD = 0.422 ± 0.0851 | rate ± SD = 0.304 ± 0.0620 |  |
| Random effects (Variable = intercept) | |  | |
| Batch | SD = 0.252, Var = 0.06351 | - |  |
